# Supplementary material for: Global patterns in price elasticities of sugar-sweetened beverage intake and potential effectiveness of tax policy: a cross-sectional study of 164 countries by sex, age and global-income decile
Source: BMJ Open. 2019 Aug 8;9(8):e026390. doi: 10.1136/bmjopen-2018-026390 (PMC6701823; doi:10.1136/bmjopen-2018-026390)
Supplement: Supplementary file 1 [file bmjopen-2018-026390supp001.pdf]

Global patterns in price elasticities of sugar-sweetened beverage intake and potential effectiveness of tax policy: a cross-sectional study of 164 countries by sex, age, and global-income decile

Andrew Muhammad,[1] Birgit Meade,[2] David R Marquardt,[2] and Dariush Mozaffarian[3]

[1]University of Tennessee Institute of Agriculture, Department of Agricultural and Resource Economics, Knoxville, Tennessee, USA
[2]United States Department of Agriculture, Economic Research Service, Market and Trade Economics Division, Washington, D.C., USA
[3]Tufts University, Friedman School of Nutrition Science and Policy, Boston, Massachusetts, USA

Supplemental Figure 1	Mean SSB intake across countries in adults, age ≥ 20
Supplemental Table 1	Description of ICP food-price categories
Supplemental Table 2	Countries included in study by region (aggregate regions used for estimation)
Supplemental Table 3	Demand model estimates for SSB intake
Supplemental Table 4	Own-price elasticities of SSB intake by age, sex, and global income decile
Technical Appendix	Demand model and methods
Supplemental Figure 2	Deflated sugar price index: unadjusted and adjusted

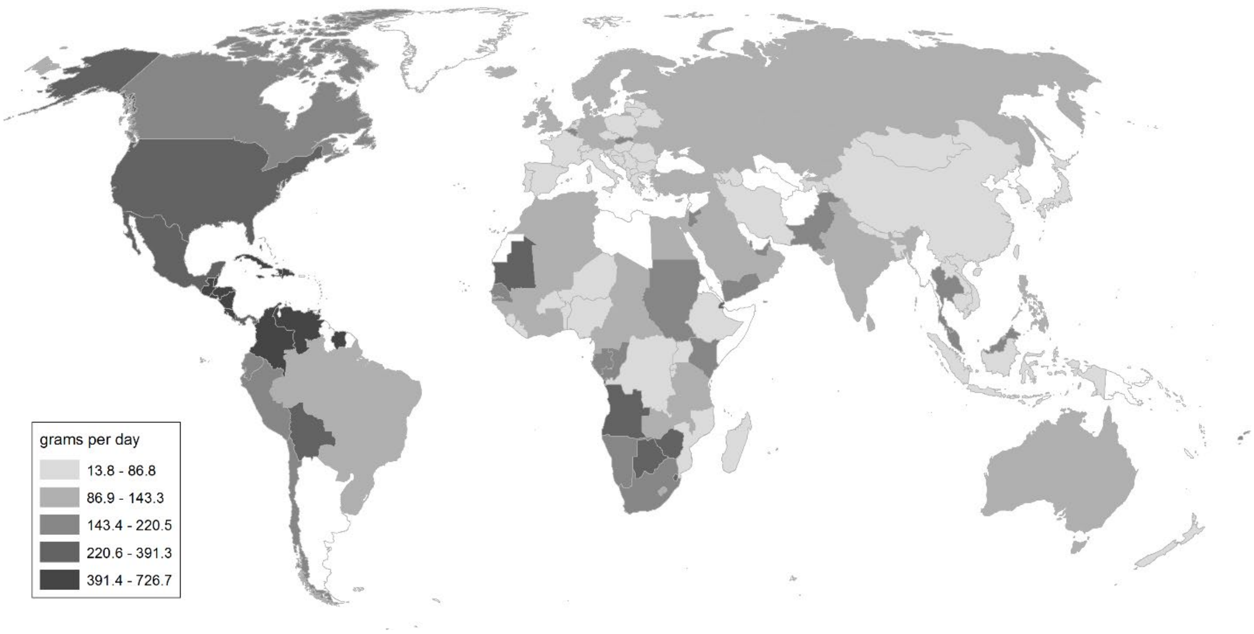

Supplemental Figure 1

Mean SSB intake across countries in adults, age ≥ 20

Map was created using ArcGIS® and ArcMap™ software by Esri. ArcGIS® and ArcMap™ are the intellectual property of Esri and are used herein under license. According to Esri citation guidelines, geodata is redistributable with value-added software applications developed by Esri on a royalty-free basis with proper metadata and copyright attribution to the respective data vendor/vendors. See Esri citation guide for more details: http://help.arcgis.com/en/arcgisdesktop/10.0/help/index.html#//001z00000003000000.htm.

Data source: Global Dietary Database, 2010

Supplemental Table 1

Description of ICP food-price categories

ICP food-price category
• *Fresh or chilled fruit* – All fresh or chilled fruit including melons and water melons; excludes vegetables grown for their fruit such as cucumbers and tomatoes.
• *Fresh milk* – Raw milk; pasteurised or sterilised milk; includes whole and low fat milk; recombined or reconstituted milk; soya milk.
• *Sugar* – Cane or beet sugar, unrefined or refined, powdered, crystallised or in lumps; includes artificial sugar substitutes.

Supplemental Table 2

Countries included in study by region (aggregate regions used for estimation)

Region	Countries
Southeast Asia, East Asia, and High Income Asia Pacific (Asia)<br>(13 countries)	Brunei Darussalam, Cambodia, China, Indonesia, Japan, South Korea, Laos, Malaysia, Maldives, Philippines, Singapore, Thailand, and Vietnam.
Central Europe, Eastern Europe, and Central Asia (CEE/C. Asia)<br>(27 countries)	Albania, Armenia, Azerbaijan, Belarus, Bosnia and Herzegovina, Bulgaria, Croatia, Czech Republic, Estonia, Georgia, Hungary, Kazakhstan, Kyrgyz Republic, Latvia, Lithuania, Macedonia, Moldova, Mongolia, Montenegro, Poland, Romania, Russia, Serbia, Slovak Republic, Slovenia, Tajikistan, and Ukraine.
Latin America and the Caribbean (LAC)<br>(30 countries)	Antigua and Barbuda, Bahamas, Barbados, Belize, Bolivia, Brazil, Chile, Colombia, Costa Rica, Cuba, Dominica, Dominican Republic, Ecuador, El Salvador, Grenada, Guatemala, Haiti, Honduras, Jamaica, Mexico, Nicaragua, Panama, Paraguay, Peru, St. Lucia, St. Vincent and Grenadines, Suriname, Trinidad and Tobago, Uruguay, and Venezuela.
Middle East, North Africa, and South Asia (MENA/S. Asia)<br>(23 countries)	Algeria, Bahrain, Bangladesh, Bhutan, Egypt, India, Iran, Iraq, Israel, Jordan, Kuwait, Morocco, Nepal, Oman, Pakistan, Qatar, Saudi Arabia, Sri Lanka, Tunisia, Turkey, United Arab Emirates, West Bank and Gaza, and Yemen.
Sub-Saharan Africa (SSA)<br>(45 countries)	Angola, Benin, Botswana, Burkina Faso, Burundi, Cameroon, Cape Verde, Central African Republic, Chad, Comoros, Congo, Côte d'Ivoire, Djibouti, DR Congo, Equatorial Guinea, Ethiopia, Gabon, Gambia, Ghana, Guinea, Guinea-Bissau, Kenya, Lesotho, Liberia, Madagascar, Malawi, Mali, Mauritania, Mauritius, Mozambique, Namibia, Niger, Nigeria, Rwanda, São Tomé and Principe, Senegal, Sierra Leone, South Africa, Sudan, Swaziland, Tanzania, Togo, Uganda, Zambia, and Zimbabwe.
High Income/Rest of World (HIC)<br>(26 countries)	Australia, Austria, Belgium, Canada, Cyprus, Denmark, Fiji, Finland, France, Germany, Greece, Iceland, Ireland, Italy, Luxembourg, Malta, Netherlands, New Zealand, Norway, Portugal, Seychelles, Spain, Sweden, Switzerland, United Kingdom, and United States.

Supplemental Table 3

Demand model estimates for SSB intake

Variable	model 1 estimate (SE)	model 2 estimate (SE)	model 3 (final model) estimate (SE)
constant	436.63 (25.47)***	-784.78 (378.46)**	-1,398.66 (535.82)***
female (F)	-13.36 (0.82)***	-13.36 (0.82)***	28.17 (15.92)*
age	-10.87 (0.74)***	-10.87 (0.74)***	14.06 (11.51)
age^2	0.08 (0.01)***	0.08 (0.01)***	-0.15 (0.09)*
SSA	1.83 (11.47)	35.51 (17.22)**	78.19 (37.85)**
LAC	258.41 (26.38)***	251.12 (28.01)***	546.89 (62.00)***
MENA/S. Asia	-10.05 (10.57)	7.45 (13.56)	21.20 (28.71)
CEE/C. Asia	-32.87 (9.99)***	-18.44 (12.59)	-28.38 (26.74)
Asia	-26.85 (14.66)*	-59.53 (19.50)***	-121.47 (41.21)***
age × SSA			-0.85 (0.42)**
age × LAC			-5.92 (0.69)***
age × MENA/S. Asia			-0.28 (0.32)
age × CEE/C. Asia			0.20 (0.30)
age × Asia			1.24 (0.45)***
$\log(P_s)$		-42.65 (15.23)***	-483.47 (116.99)***
$F \times \log(P_s)$			12.42 (2.33)***
$Age \times \log(P_s)$			9.89 (1.20)**
$Age^2 \times \log(P_s)$			-0.09 (0.01)***
$\log(P_f)$		85.90 (25.27)***	231.34 (83.87)***
$F \times \log(P_f)$			-1.38 (2.77)
$Age \times \log(P_f)$			-4.15 (2.22)*
$Age^2 \times \log(P_f)$			0.02 (0.02)
$\log(P_m)$		53.30 (20.73)***	107.80 (72.51)
$F \times \log(P_m)$			1.57 (2.63)
$Age \times \log(P_m)$			-0.97 (1.98)
$Age^2 \times \log(P_m)$			0.00 (0.02)
$\log(Y)$		145.85 (45.34)***	379.56 (109.52)***
$F \times \log(Y)$			-6.23 (2.17)***
$Age \times \log(Y)$			-3.49 (1.53)**
$Age^2 \times \log(Y)$			0.03 (0.01)***
$\log(Y)^2$		-8.77 (3.04)***	-18.97 (6.48)***
$\log(P_s) \times \log(Y)$			19.87 (10.73)*
Adjusted R^2	0.65	0.70	0.80

Note: Dependent variable is SSB intake in g/d. Standard errors are in (parentheses).
*p≤0.10; **p≤0.05; ***p≤0.01. SSA = Sub-Saharan Africa. LAC = Latin America and the Caribbean. MENA/S. Asia = Middle East, North Africa, and South Asia. CEE/C. Asia = Central Europe, Eastern Europe, and Central Asia. Asia = Asian Pacific, East Asia, and Southeast Asia. The reference region consist of high-income Western countries and a few small island states. P_s = SSB price, P_f = fruit juice price, P_m = milk price. All prices were deflated by a food price index. Y = real per capita income.

Supplemental Table 4

Own-price elasticities of SSB intake by age, sex, and global income decile

Income decile	Age 20	25	30	35	40	45	50	55	60	65	70	75	80
	Women												
Lowest	-0.90	-0.84	-0.80	-0.77	-0.78	-0.76	-0.70	-0.71	-0.78	-0.92	-1.11	-1.40	-1.84
10%	(0.21)	(0.23)	(0.25)	(0.29)	(0.35)	(0.40)	(0.42)	(0.45)	(0.49)	(0.52)	(0.53)	(0.55)	(0.60)
2nd	-0.83	-0.76	-0.71	-0.67	-0.65	-0.60	-0.54	-0.52	-0.58	-0.70	-0.88	-1.16	-1.59
	(0.18)	(0.19)	(0.21)	(0.24)	(0.29)	(0.33)	(0.34)	(0.36)	(0.38)	(0.41)	(0.41)	(0.43)	(0.46)
3rd	-0.76	-0.69	-0.62	-0.56	-0.51	-0.44	-0.36	-0.33	-0.37	-0.48	-0.65	-0.92	-1.33
	(0.16)	(0.17)	(0.18)	(0.20)	(0.23)	(0.26)	(0.27)	(0.28)	(0.29)	(0.30)	(0.30)	(0.31)	(0.34)
4th	-0.70	-0.62	-0.54	-0.47	-0.40	-0.31	-0.22	-0.17	-0.19	-0.28	-0.45	-0.71	-1.10
	(0.14)	(0.15)	(0.16)	(0.17)	(0.20)	(0.21)	(0.22)	(0.22)	(0.22)	(0.22)	(0.21)	(0.21)	(0.24)
5th	-0.67	-0.58	-0.49	-0.40	-0.32	-0.22	-0.12	-0.07	-0.07	-0.15	-0.32	-0.57	-0.96
	(0.14)	(0.14)	(0.15)	(0.16)	(0.18)	(0.20)	(0.19)	(0.19)	(0.19)	(0.18)	(0.17)	(0.16)	(0.18)
6th	-0.64	-0.54	-0.45	-0.35	-0.26	-0.14	-0.04	0.02	0.02	-0.05	-0.21	-0.46	-0.84
	(0.13)	(0.13)	(0.14)	(0.15)	(0.17)	(0.19)	(0.18)	(0.18)	(0.18)	(0.17)	(0.15)	(0.14)	(0.16)
7th	-0.60	-0.51	-0.41	-0.31	-0.20	-0.07	0.04	0.10	0.11	0.05	-0.11	-0.35	-0.72
	(0.13)	(0.13)	(0.14)	(0.15)	(0.17)	(0.19)	(0.19)	(0.18)	(0.18)	(0.17)	(0.15)	(0.14)	(0.16)
8th	-0.57	-0.46	-0.36	-0.25	-0.13	0.01	0.13	0.20	0.23	0.17	0.02	-0.22	-0.58
	(0.13)	(0.13)	(0.14)	(0.16)	(0.18)	(0.20)	(0.20)	(0.20)	(0.20)	(0.19)	(0.18)	(0.17)	(0.19)
9th	-0.53	-0.42	-0.31	-0.18	-0.05	0.11	0.23	0.32	0.35	0.31	0.16	-0.08	-0.43
	(0.14)	(0.14)	(0.15)	(0.17)	(0.20)	(0.22)	(0.22)	(0.23)	(0.24)	(0.24)	(0.23)	(0.23)	(0.24)
Highest	-0.47	-0.36	-0.23	-0.10	0.06	0.23	0.37	0.47	0.52	0.49	0.35	0.12	-0.22
10%	(0.15)	(0.16)	(0.17)	(0.19)	(0.23)	(0.26)	(0.27)	(0.29)	(0.30)	(0.32)	(0.31)	(0.32)	(0.34)
	Men												
Lowest	-0.87	-0.82	-0.79	-0.79	-0.83	-0.85	-0.81	-0.83	-0.91	-1.06	-1.24	-1.50	-1.91
10%	(0.19)	(0.21)	(0.23)	(0.26)	(0.32)	(0.37)	(0.39)	(0.42)	(0.45)	(0.48)	(0.50)	(0.51)	(0.55)
2nd	-0.81	-0.75	-0.71	-0.69	-0.71	-0.70	-0.66	-0.66	-0.73	-0.85	-1.03	-1.28	-1.68
	(0.17)	(0.18)	(0.19)	(0.22)	(0.27)	(0.31)	(0.32)	(0.34)	(0.36)	(0.38)	(0.39)	(0.4)	(0.43)
3rd	-0.75	-0.68	-0.63	-0.60	-0.59	-0.56	-0.50	-0.48	-0.53	-0.64	-0.81	-1.06	-1.44
	(0.15)	(0.15)	(0.16)	(0.18)	(0.22)	(0.25)	(0.25)	(0.26)	(0.27)	(0.28)	(0.28)	(0.29)	(0.32)
4th	-0.69	-0.62	-0.56	-0.51	-0.48	-0.43	-0.36	-0.33	-0.36	-0.46	-0.62	-0.87	-1.24
	(0.13)	(0.14)	(0.14)	(0.16)	(0.18)	(0.20)	(0.20)	(0.20)	(0.21)	(0.21)	(0.20)	(0.21)	(0.23)
5th	-0.66	-0.58	-0.51	-0.46	-0.41	-0.34	-0.27	-0.23	-0.25	-0.34	-0.50	-0.74	-1.10
	(0.13)	(0.13)	(0.13)	(0.15)	(0.17)	(0.18)	(0.18)	(0.18)	(0.18)	(0.17)	(0.16)	(0.16)	(0.19)
6th	-0.63	-0.55	-0.48	-0.41	-0.35	-0.28	-0.19	-0.15	-0.17	-0.25	-0.40	-0.64	-0.99
	(0.12)	(0.13)	(0.13)	(0.14)	(0.16)	(0.18)	(0.17)	(0.17)	(0.17)	(0.16)	(0.14)	(0.14)	(0.17)
7th	-0.60	-0.52	-0.44	-0.37	-0.30	-0.21	-0.12	-0.07	-0.08	-0.16	-0.31	-0.54	-0.89
	(0.12)	(0.12)	(0.13)	(0.14)	(0.16)	(0.18)	(0.17)	(0.17)	(0.17)	(0.16)	(0.14)	(0.14)	(0.16)
8th	-0.57	-0.48	-0.40	-0.32	-0.23	-0.13	-0.04	0.02	0.02	-0.04	-0.19	-0.42	-0.76
	(0.12)	(0.13)	(0.13)	(0.15)	(0.17)	(0.18)	(0.18)	(0.18)	(0.18)	(0.18)	(0.17)	(0.17)	(0.19)
9th	-0.53	-0.44	-0.35	-0.26	-0.16	-0.04	0.06	0.13	0.14	0.08	-0.06	-0.29	-0.62
	(0.13)	(0.13)	(0.14)	(0.16)	(0.18)	(0.20)	(0.21)	(0.21)	(0.22)	(0.22)	(0.21)	(0.21)	(0.23)
Highest	-0.49	-0.39	-0.28	-0.18	-0.06	0.07	0.19	0.27	0.29	0.25	0.11	-0.11	-0.43
10%	(0.14)	(0.14)	(0.15)	(0.18)	(0.21)	(0.24)	(0.25)	(0.27)	(0.28)	(0.29)	(0.29)	(0.29)	(0.31)

Note: Values are derived at median intake levels by demographic subgroup. Standard errors are in (parentheses). Price elasticities are based on 1% price changes. Income deciles are based on the national income of the 164 countries included in the study. Each decile is comprised of 16 countries except the 4 lowest deciles, which are each comprised of 17 countries. The per capita income range (PPP-adjusted in thousand $US) for each decile: (1st) $0.6-$1.5, (2nd) $1.5-$2.7, (3rd) $2.7-$5.3, (4th) $5.5-$8.0, (5th) $8.3-$10.8, (6th) $11.1-$15.2, (7th) $15.3-$20.3, (8th) $20.6-$29.4, (9th) $30.4-$40.9, and (10th) $41.3-$127.2.

Technical Appendix

Demand model and methods

To estimate SSB intake demand, we used a semi-logarithmic functional form that has been proven to be consistent with economic theory and rational consumer behavior.(1, 2) We applied a single-equation framework in this study. Prior studies have used a demand-system approach (multi-equation framework), primarily due to the adding-up property when using expenditure data (i.e., expenditures on all consumption categories "add up" to total expenditures), which results in the error terms being correlated across categories. Since this relationship does not exist with individual intakes, particularly when the correspondence between purchases and intakes is not one to one, the adopted approach is acceptable.

Many studies have used a double-log quadratic form.(3) However, a problem with the double-log form is that significant intake differences across subgroups can be lost in log conversions. A semi-log relationship allowed for a better assessment of subgroup effects on intake responsiveness. It has also been shown that semi-log models of demand are consistent with economic theory and contain the necessary information for obtaining, for instance, reliable measures of consumer welfare and the underlying preference structure of consumers.(1)

Let q_{ig} represent SSB intake by subgroup g (g: sex and age), p_i and p_j represent the price of SSBs and related good j, and Y and P represent real per capita income and overall food prices (all in country *C*). SSB intake demand by subgroup *g* in country *C* is specified as follows (C subscripts are omitted for convenience):

$$q_{ig} = \beta_0^* + \beta_1^* \ln(Y) + \beta_2^* \ln\left(\frac{p_i}{P}\right) + \beta_3^* \ln\left(\frac{p_j}{P}\right) + \beta_4^* \left[\ln(Y) \times \ln\left(\frac{p_i}{P}\right)\right] + \beta_5^* \ln(Y)^2 + u_{ig} \quad (1)$$

The β terms are coefficients to be estimated and u_{ig} is a random error term. The price terms (p_i and p_j) are deflated by P to discount price differences due to overall food prices and to implicitly account for the cross-price effects of intake categories other than i and j. Note that the structure of the model allows for the relationship between own-price (p_i) and intake to vary by national income level.

We accounted for age, sex, and regional differences by allowing these factors to have a direct effect on intake, as well as an additional effect through income and prices. Thus, the beta terms (β_k^*) were expanded to account for age, sex, and region interactions.

$$\beta_k^* = f(sex, age, region) \forall k \quad (2)$$

The variable *sex* is a binary (= 1 for women and 0 otherwise) and *age* is a variable ranging from 20 to 80 in 5-year intervals. We also considered age^2 to allow for nonlinear age effects and the possibility of optimal responsiveness between the youngest and oldest subgroup. We accounted for varying preferences across countries due to factors not related to income or prices by including six regional binary variables, including: Southeast Asia, East Asia, and High Income Asia Pacific (Asia) (13 countries); Central Europe, Eastern Europe, and Central Asia (CEE/C. Asia) (27); Latin America and the Caribbean (LAC) (30); Middle East, North Africa, and South Asia (MENA/S. Asia) (23); Sub-Saharan Africa (SSA) (45); and High Income/Rest of World (HIC) (26). HIC was comprised largely of Western, industrialized countries; while not geographically connected, these countries share other similarities. We included several small

island countries in this grouping because they were not sufficiently numerous to merit their own regional grouping.

We first estimated a model with all possible interactions and then utilized F-tests to compare that model to a series of restricted models and arrived at the final parsimonious model. All models were estimated assuming country clusters, that is, independent errors across countries but correlated errors within countries, as well as heteroskedastic-consistent errors.(4)

Given equation (1), the own-price elasticity is derived as follows:

$$\eta_{iig} = \frac{\%\Delta q_{ig}}{\%\Delta p_i} = \frac{1}{q_{ig}}[\beta_2^* + \beta_4^* \ln(Y)] \tag{3}$$

η_{iig} is the percentage change in intake (q_{ig}) (i: SSB) due to a 1% change in p_i, which should be negative since an increase in price usually results in a decrease in intake or quantity demanded. Note that if the β coefficients vary with sex, age, or region, equation (3) will vary accordingly.

Price index adjustment

We used sugar and fresh fruit price indexes as proxies for SSB and fruit juice prices, respectively. An issue with this approach is that sugar and/or fresh fruit may not account for a major share of the final product price, particularly in higher income countries. In view of this fact, we derived a multiplicative adjustment factor for the sugar and fresh fruit price indexes assuming the following quadratic relationship between the adjustment factor and real per-capita income.

$$adjustment = a + bY + cY^2 \tag{4}$$

We used a calibration method to derive values for a, b, and c. We considered the extreme case (zero income) $Y = 0$ and set $a = 1$. In this instance, equation (4) =1 and the price index value would remained unchanged:

$$adjusted\ price\ index = unadjusted\ price\ index \times 1.$$

Using information on the value-added share of farm products in the U.S. food and beverage sector, as well as qualitative information about food production costs in low-income countries, we obtained the following estimates: $b = 0.0003$ and $c = -0.0000000015$.

We adjusted the sugar and fresh fruit price indexes based on equation (4). Note that the adjustment factor starts at a value of 1 and then increases at a decreasing rate with per-capita income indicating a higher value added at higher income levels. For a country in the lowest income decile with per-capita income (Y) = \$1,000, the adjustment factor is 1.3. Assuming an *unadjusted* price index value of 0.70, the *adjusted* price index = 0.91 (0.70 ×1.3). For Switzerland, a high-income country with Y=\$50,963, the unadjusted sugar-price index = 0.63. At this income level, the adjustment factor = 12.39, and the adjusted sugar-price index = 7.81 (0.63 ×12.39). The unadjusted and adjusted sugar price indexes across countries are reported in supplemental figure 2.

References

1. LaFrance JT. Incomplete demand systems and semilogarithmic demand models. Australian J Agr Econ. 1990;34(2):118-31.
2. von Haefen RH. A complete characterization of the linear, log-linear, and semi-log incomplete demand system models. J Agr Resource Econ. 2002;27(2):281-319.
3. Sahn DE. The Effect of Price and Income Changes on Food-Energy Intake in Sri Lanka. Econ Devel Cult Change. 1988;36(2):315-40.
4. Cameron AC, Trivedi PK. Microeconometrics: Methods and Applications. Cambridge: Cambridge University Press; 2005.

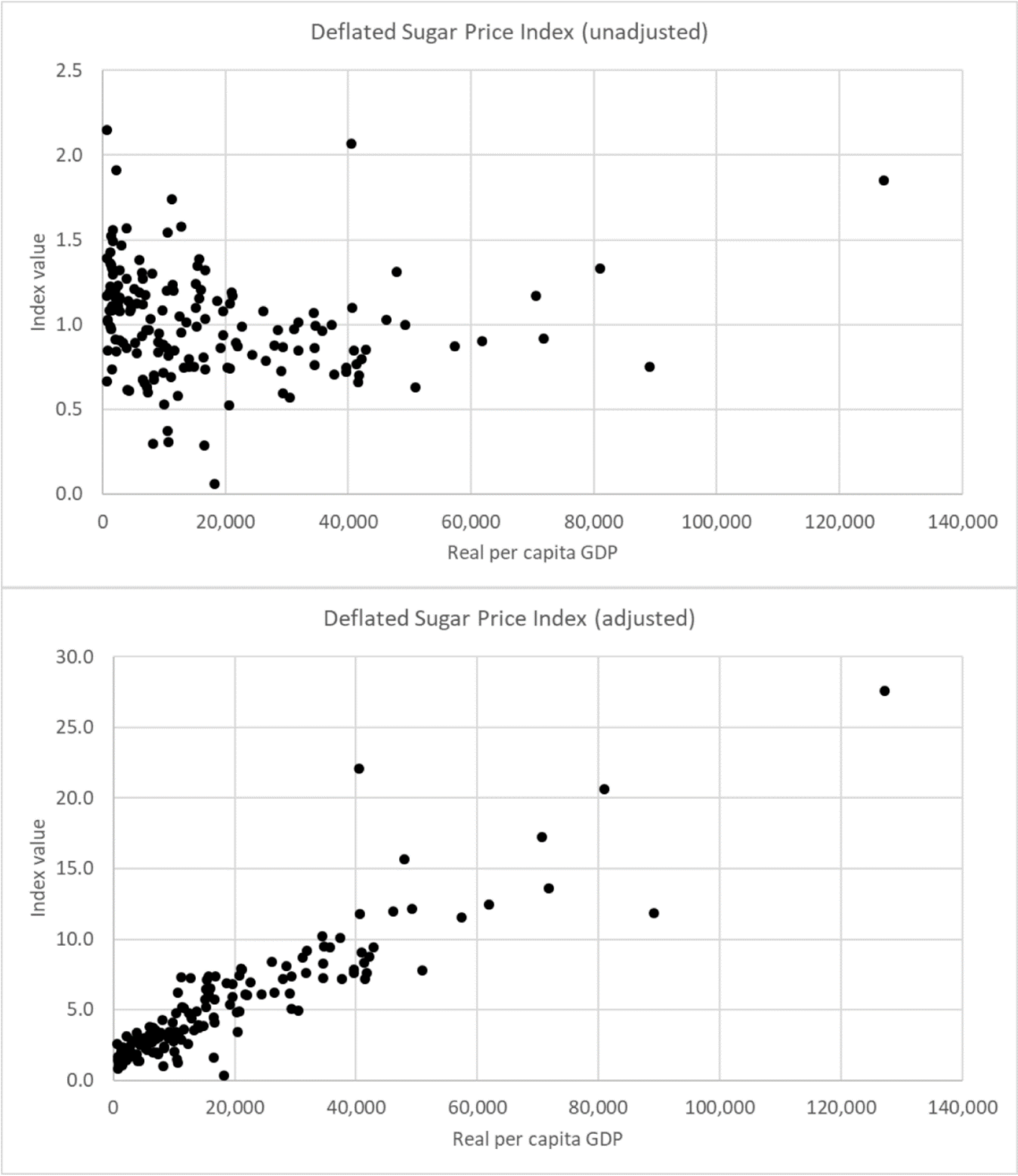

Supplemental Figure 2

Deflated sugar price index: unadjusted and adjusted

Note: prices are deflated by a total food price index to discount differences across countries due to overall food prices.

Source: World Bank International Comparison Program Data. Adjusted price index values are based on author's calculations.
