# Supplementary material for: Global patterns in price elasticities of sugar-sweetened beverage intake and potential effectiveness of tax policy: a cross-sectional study of 164 countries by sex, age and global-income decile
Source: BMJ Open. 2019 Aug 8;9(8):e026390. doi: 10.1136/bmjopen-2018-026390 (PMC6701823; doi:10.1136/bmjopen-2018-026390)
Supplement: Author's manuscript [file bmjopen-2018-026390.draft_revisions.pdf]

BMJ Open is committed to open peer review. As part of this commitment we make the peer review history of every article we publish publicly available.

When an article is published we post the peer reviewers' comments and the authors' responses online. We also post the versions of the paper that were used during peer review. These are the versions that the peer review comments apply to.

The versions of the paper that follow are the versions that were submitted during the peer review process. They are not the versions of record or the final published versions. They should not be cited or distributed as the published version of this manuscript.

BMJ Open is an open access journal and the full, final, typeset and author-corrected version of record of the manuscript is available on our site with no access controls, subscription charges or pay-per-view fees (http://bmjopen.bmj.com).

If you have any questions on BMJ Open's open peer review process please email info.bmjopen@bmj.com

BMJ Open

Global patterns in price elasticities of sugar-sweetened beverage intake and potential effectiveness of tax policy: results from 164 countries

Journal:	*BMJ Open*
Manuscript ID	bmjopen-2018-026390
Article Type:	Research
Date Submitted by the Author:	30-Aug-2018

Complete List of Authors:	Muhammad, Andrew; University of Tennessee Institute of Agriculture<br>Meade, Birgit; USDA Economic Research Service<br>Marquardt, David; USDA Economic Research Service<br>Mozaffarian, Dariush; Friedman School of Nutrition Science and Policy, Tufts University,

Keywords:	elasticity, intake, global, prices, sugar-sweetened beverages, taxes

SCHOLARONE™
Manuscripts

For peer review only

Global patterns in price elasticities of sugar-sweetened beverage intake and potential effectiveness of tax policy: results from 164 countries

Andrew Muhammad,[1] Birgit Meade,[2] David R Marquardt,[2] and Dariush Mozaffarian[3]

(Corresponding author) Andrew Muhammad, University of Tennessee Institute of Agriculture, Department of Agricultural and Resource Economics, 2621 Morgan Circle, 302 Morgan Hall Knoxville, Tennessee 37996-4518, USA

Email: amuhammad@utk.edu

[1]University of Tennessee Institute of Agriculture, Department of Agricultural and Resource Economics, Knoxville, Tennessee, USA

[2]United States Department of Agriculture, Economic Research Service, Market and Trade Economics Division, Washington, D.C., USA

[3]Tufts University, Friedman School of Nutrition Science and Policy, Boston, Massachusetts, USA

Keywords: demand, diet, elasticity, intake, global, prices, sugar-sweetened beverages, taxes

Disclaimer: The views expressed are those of the authors, and may not be attributed to the Economic Research Service or the U.S. Department of Agriculture.

Copyright: The Corresponding Author has the right to grant on behalf of all authors and does grant on behalf of all authors, a worldwide licence (or non-exclusive for government employees) to the Publishers and its licensees in perpetuity, in all forms, formats and media (whether known now or created in the future), to i) publish, reproduce, distribute, display and store the Contribution, ii) translate the Contribution into other languages, create adaptations, reprints, include within collections and create summaries, extracts and/or, abstracts of the Contribution, iii) create any other derivative work(s) based on the Contribution, iv) to exploit all subsidiary rights in the Contribution, v) the inclusion of electronic links from the Contribution to third party material where-ever it may be located; and, vi) licence any third party to do any or all of the above.

For peer review only - http://bmjopen.bmj.com/site/about/guidelines.xhtml

Competing interests: We have read and understood BMJ policy on declaration of interests and declare that we have no competing interests.

Contributors: AM conceptualized the study and was responsible for the study design, model estimations, and contributed to the interpretation of results. AM, DM, and BM contributed to the interpretation of results and discussion. DM wrote and edited sections describing the intake data. BM was primarily responsible for the literature review and facilitated the data agreement with the International Comparison Program, World Bank. DM provided the intake data and obtained the funding. DRM and AM were responsible for the visualizations and corresponding text. AM was the primary author, but all authors contributed to writing the manuscript. AM is the manuscript's guarantor.

Data sharing: No additional data available.

Ethical approval: Not required.

Clinical trial registration: Not required.

Transparency declaration: The lead author* affirms that this manuscript is an honest, accurate, and transparent account of the study being reported; that no important aspects of the study have been omitted; and that any discrepancies from the study as planned (and, if relevant, registered) have been explained. *The manuscript's guarantor.

Funding: The Bill & Melinda Gates Foundation (project: Global Dietary Habits among Women, Price and Income Elasticities, and Validity of Food Balance Sheets).

Role of the funding source: The funders had no role in the study design, study conduct, data analysis, data interpretation, or writing of the report. All authors had full access to all the data and take responsibility for the integrity of the data and the accuracy of the analysis. The corresponding author had final responsibility to submit the report for publication.

For peer review only

Abstract

Objective – To quantify global relationships between sugar-sweetened beverage (SSB) intake and prices and examine the potential effectiveness of tax policy by age, sex, and country.

Design – Cross-country intake demand model.

Setting – 164 countries.

Population – Full adult population in each country.

Main outcome measures – A consumer demand modeling framework was used to estimate the relationship between SSB intake and prices (own and substitute prices) and derived price elasticities of SSB intake (measures of the percentage change in intake from a 1% change in price) by age, sex, and country. We simulated how a 20% tax would impact SSB intake globally. Tax policy outcomes (projected intake reductions) were examined across countries by income decile for representative age and sex subgroups.

Results – Own-price responsiveness of SSB intake was highest in lowest income countries, ranging from -0.58 (95% CI: -1.01, -0.15) for women, age 20, to -1.10 (-2.10, -0.10) for men, age 80, compared to -0.29 (-0.58, -0.00) to -0.09 (-0.62, 0.44) in highest income countries. In lower income countries, elasticities were strongest (became more negative) at older ages; in middle-income countries, there was little influence of age on elasticities; in higher income countries, price elasticities were strongest at younger ages. Although women, overall, had a slightly lower response to price changes than men, sex differences were mostly negligible. Potential intake reductions from a 20% national tax in lowest income countries range from 12.0% (2.7%, 21.4%) in women, age < 35, to 19.6% (1.6%, 37.6%) in men, age ≥ 60. Potential intake reductions decreased with country income overall, and also decreased with older age groups in highest income countries.

Conclusions – These novel findings estimate the global price-responsiveness of SSB intake by country, age, and sex, informing ongoing policy discussions on potential effects of SSB taxes.

For peer review only

Strengths and limitations of this study

- First study to examine SSB intake and taxation in a global context, providing a better understanding of tax-policy effectiveness across the complete spectrum of countries.
- Results quantify the potential variability in influence of price on SSB intake across countries including by age and sex, suggesting that outcomes of SSB taxes may be significantly influenced by age and the income status of countries.
- Findings suggest that a sufficiently high SSB tax will significantly reduce intake across all ages, sexes, and national incomes except in the wealthiest nations.
- Being a modeling study, the projected outcomes can only inform how taxes could affect behavior.

For peer review only

Introduction

Taxation of sugar-sweetened beverages (SSBs) has received growing attention, given their links to excessive weight gain and increased risk of obesity, type-2 diabetes, and other non-communicable diseases (NCDs).(1-5) Arguably, taxation is not punitive but market normalizing, as the true costs of SSBs due to public health-care expenditures and other societal costs from excessive intake are not reflected in current market prices. Thus, by increasing SSB prices relative to other foods, taxes can play a role in decreasing consumption, lowering societal costs, and improving societal wellbeing.(6, 7) Based on these considerations, a rapidly growing number of countries have implemented or announced national SSB taxes, (8, 9) including Norway in 1981 and Samoa in 1984; Australia, French Polynesia, Fiji, and Nauru between 2000 and 2007; and Finland, Hungary, France, Chile, Mexico, Barbados, St. Helena, and Dominica since 2011. South Africa, Colombia, the UK, and Saudi Arabia have included such taxes in recent proposals; while India, Indonesia, and the Philippines are considering similar measures. In the U.S., more than 30 jurisdictions have implemented or attempted to pass SSB taxes, including six U.S. cities since the 2016 elections.(10) Despite their growing acceptance globally, the potential impact of SSB taxation on intake remains uncertain, particularly how it might vary across countries, and by age and sex within countries.

Most studies of SSB taxation have been limited to a small group of countries or focused on a specific country or jurisdiction where taxes have been implemented.(11-16) No study to date has examined SSB consumption and taxation in a global context. In addition, few studies have considered how SSB intake could vary depending on the price of substitute products, such as fruit juice or milk.(17) Because expert organizations are advocating and governments are considering SSB taxation across the globe,(18) examining demand in a global context can

For peer review only

For peer review only - http://bmjopen.bmj.com/site/about/guidelines.xhtml

provide a better understanding of potential tax-policy effectiveness across the complete spectrum of countries, from most to least developed.

To investigate this issue, we examined SSB intake across 164 countries and estimated how intake differences within and across countries are influenced by the price of SSBs and substitute caloric beverages (fruit juice and milk), as well as other factors such as national income, age, and sex. Based on WHO recommendations,(18) we further simulated how SSB intake would respond to a 20% tax (price increase). Tax-policy outcomes were examined across countries by income decile for representative age and sex subgroups.

Methods

Using globally representative intake and pricing data, we implemented a consumer demand modeling framework to examine determinants of SSB intake within and across countries. The modeling framework accounted for age and sex differences and economic determinants such as own price, price of substitutes (fruit juice and milk), and real per capita income at the national level. We also considered the potential for unmeasured region-specific differences, such as in taste or other preferences, by including regional binary variables. Model estimates were used to derive SSB price elasticities for detailed strata (age, sex, and country), as well as at more aggregate levels (countries by income decile), and to assess the potential impact of taxes on intake. Accounting for these factors, we report price elasticities of SSB intake (measures of the percentage change in intake from a 1% change in price), which have been a primary means of estimating potential tax-policy effectiveness.(19) We also evaluated the variability in tax-policy effectiveness and examined outcomes for select age and sex subgroups and countries by income decile.

For peer review only - http://bmjopen.bmj.com/site/about/guidelines.xhtml

Data and sources

Data on intakes of SSBs were derived from the 2010 Global Dietary Database (GDD), a database of global food and nutrient intakes by age (20-80 in 5-year intervals) and sex for 187 countries. The SSB category in the GDD includes intake of all sugar-sweetened beverages, including any beverage with added sugar and ≥ 50 kcal per 8 oz., such as carbonated beverages, sodas, energy drinks, fruit drinks, etc., excluding 100% juices. The GDD data collection, statistical methods, data validation, and findings have been described in detail (also see http://www.globaldietarydatabase.org/).(20-24) In brief, GDD data were derived based on national and subnational dietary surveys, informed by additional information from United Nations Food and Agricultural Organization (FAO) food balance sheets data, individual-level surveys from cohort studies, household expenditure surveys when dietary surveys were not available, as well as other data sources such as the World Health Organization (WHO) Global Infobase and the WHO STEPS database.(24)

For prices, we used global price level indices from the 2011 International Comparison Program (ICP) of the World Bank.(25, 26) The ICP is a worldwide statistical initiative that estimates purchasing power parities (PPPs), which are spatial indices comparing the price of a given basket of goods and services across countries relative to a base country. Price level indices are PPPs standardized to a common currency, the U.S. dollar in this case. Our choice of price variables is limited by inadequate data on a global scale. For instance, the ICP categories included milk but not SSBs and fruit juice; for SSBs and fruit juice, we used sugar and fresh-fruit price indices as proxies since these are primary inputs in their respective production . Since demand is influenced by relative prices, we divided each price series by an aggregate price level

For peer review only - http://bmjopen.bmj.com/site/about/guidelines.xhtml

index for *food and nonalcoholic beverages* to adjust for differences in overall food prices across countries behavior (see supplemental table 1). The current analysis included 164 countries (4,264 stratum observations) having both GDD intake and ICP price data.

For national income, we used 2010 gross domestic product (GDP) data expressed in U.S. dollars per capita from the World Bank Development Indicators Database.(27) To account for differences in currency and purchasing power across economies, we used PPP-adjusted GDP. Since PPP-adjusted GDP accounts for inflationary factors across countries, we refer to our income measure as *real* per capita GDP. Income deciles were based on real per capita GDP for the 164 countries in the study.

Model and analysis

To estimate SSB intake demand, we applied a single-equation framework and used a semi-logarithmic functional form that has been proven to be consistent with economic theory and rational consumer behavior (see supplementary information, technical appendix).(28, 29) Prior studies have used a demand-system approach (multi-equation framework), primarily due to the adding-up property when using expenditure data (i.e., expenditures on all consumption categories "add up" to total expenditures), which results in the error terms being correlated across categories. Since this relationship does not exist with individual intakes, particularly when the correspondence between purchases and intakes is not one to one, the adopted approach is acceptable.

We accounted for age, sex, and regional differences by allowing these factors to have a direct effect on intake, as well as an additional effect through income and prices, including a

For peer review only - http://bmjopen.bmj.com/site/about/guidelines.xhtml

quadratic age term to allow for nonlinear effects and the possibility of optimal responsiveness being between the youngest and oldest subgroups.

We accounted for preferences across countries due to factors not related to income or prices by including regional binary variables in the model: Southeast Asia, East Asia, and High Income Asia Pacific (Asia) (13 countries); Central Europe, Eastern Europe, and Central Asia (CEE/C. Asia) (27); Latin America and the Caribbean (LAC) (30); Middle East, North Africa, and South Asia (MENA/S. Asia) (23); Sub-Saharan Africa (SSA) (45); and High Income/Rest of World (HIC) (26). HIC was comprised largely of Western, industrialized countries; while not geographically connected, these countries share other similarities. We included several small island countries in this grouping because they were not sufficiently numerous to merit their own regional grouping (see supplemental table 2).

We utilized F-tests to compare a model including all explanatory variables and interaction terms to a series of restricted models and arrived at the final parsimonious model. Least-squares regression treats data independently and does not account for within-country correlations, resulting in biased and comparatively small standard errors. Consequently, all models were estimated assuming country clusters, that is, independent errors across countries but correlated errors within countries.(30) The elasticities reported in the following section were derived using the estimated coefficients from model 3 (final model) (see supplemental table 3).

Given WHO recommendations, we simulated how SSB intake would respond to a 20% tax (price increase).(18) Results were evaluated across countries by income decile for the following demographic subgroups: men and women, ages < 35, 35-59, ≥ 60 years. We used probabilistic sensitivity analyses (Monte Carlo simulations) to derive 95% confidence intervals of intake responsiveness to the tax. Confidence intervals were based on the covariance matrix of

For peer review only - http://bmjopen.bmj.com/site/about/guidelines.xhtml

the estimated coefficients, which accounted for the variability in the own-price relationship and the additional variability due to age, sex, and national income level.

Patient and public involvement

Patients and public were not involved.

Results

Global SSB intake

SSB intake levels varied significantly across countries (see supplemental figure 1) and by world region and age (figure 1). LAC had the highest median intake at 311 g/d (men) and 288 g/d (women) – almost four times the intake in SSA, and six times the lowest intake region (Asia). Across age/sex strata globally, the group with the highest median intake was young men, age 20 (209 g/d), followed closely by young women, age 20 (188 g/d). Compared to 20-year olds, median global intake in men and women, age 80, was about 75% lower. Across age and sex strata worldwide, the highest intake level was observed for men, age 20, in Trinidad and Tobago (1,239 g/d), and the lowest intake for women, age 80, in China (6 g/d). A more detailed discussion of global SSB intake by age, sex, and world region is available.(31)

SSB own-price elasticities

We derived SSB own-price elasticities at the most detailed level (age, sex, and country) and compared estimates across regions (figure 2). Globally, the median own-price elasticity of SSB intake was modestly lower for women (elasticity: -0.30) than for men (-0.41). Differences between regions were larger. Overall, LAC had the lowest median responsiveness at -0.09

For peer review only - http://bmjopen.bmj.com/site/about/guidelines.xhtml

and -0.12 (women and men, respectively) compared to, for instance, SSA (-0.68 and -0.76) and Central Asia (-0.74 and -0.87).

Interactions between the regional variables and prices and income were insignificant and excluded from the final model. Thus, observed elasticity differences across countries and regions are primarily influenced by country or regional intake levels. Given the variables in the final model, it was more appropriate to derive elasticities across country groups based on income level. We derived and compared SSB own-price elasticities across all strata jointly by age, sex, and global income decile (figure 3 and table 1; also see supplemental table 4). Note that reported values are derived at median intake levels by age and sex subgroup. Thus, observed differences across age, sex, and income decile are solely a function of model estimates. At any given age, SSB intake became less responsive to price changes with rising national income. For instance, in men, age 20, the median own-price elasticities ranged from -0.58 (95% CI: -0.97, -0.19) for the lowest income decile to -0.32 (-0.58, -0.06) for the highest income decile. The decline in responsiveness became more pronounced with age. For instance, in men, age 80, the median own-price elasticities ranged from -1.10 (-2.10, - 0.10) for the lowest income decile to -0.09 (-0.62, 0.44) for the highest income decile. The influence of age on SSB own-price elasticities varied depending on the income status of countries. In lower income countries, elasticities were strongest (became more negative) at older ages; in middle-income countries, there was little influence of age on elasticities; while in higher income countries, price elasticities were strongest at younger ages.

Potential impact of SSB taxes on intake

For peer review only - http://bmjopen.bmj.com/site/about/guidelines.xhtml

Potential reductions in median intake from a 20% tax (price increase) were largest in lowest income countries, ranging from 12% to nearly 20%, depending on age and sex (table 2). Across global income deciles, reductions varied less in younger adults (age < 35) – for example, ranging from 12.1% (95% CI: 3.7, 20.6) in young men in the lowest income decile to 6.1% (0.7, 11.5) in the highest income decile – than in older adults (e.g., men age 60+ years). This is consistent with the much higher baseline SSB intakes among younger adults globally (figure 1), suggesting that such intake will be significantly influenced by taxes regardless of country income status. Older men and women (age ≥ 60) in lowest income countries were estimated to be most influenced by SSB taxes, suggesting a high price-responsiveness to such a luxury in poor nations globally. In middle-income countries, older adults were as responsive as younger adults, perhaps related to relatively high SSB intakes among older adults in these nations, suggesting uniform price-sensitivity regardless of age in middle national incomes. Insignificant outcomes were mostly observed for age and sex subgroups in wealthy nations (8-10th income decile) and women, age ≥ 60, for whom intake is already quite low.

Discussion

In this global analysis of SSB intakes and prices, we identified significant price responsiveness of SSB intakes in nearly every age, sex, and country income subgroup worldwide. We also identified significant heterogeneity in these potential responses. By region, price responsiveness was highest in SSA and Central Asia and lowest in LAC. Men, overall, had a modestly stronger response to price changes than women. Price responsiveness was higher in lower income than in wealthier countries, consistent with expectations and the much higher relative share of income spent on food and other necessities in low-income countries.

For peer review only - http://bmjopen.bmj.com/site/about/guidelines.xhtml
For peer review only

Interestingly, the response by age varied by country income. In lower income countries, own-price responsiveness increased with age, in middle-income countries, own-price responsiveness was relatively constant across ages, while in the highest income countries, own-price responsiveness was higher in younger adults.

Finally, our estimates of effects of a 20% tax suggested significant SSB intake reductions across all ages, sexes, and national incomes except in oldest adults in wealthiest nations, and older women globally.

Since taxes raise the price of SSBs relative to other goods, it is important to note that consumers may choose to substitute other beverages for SSBs. We estimated how SSB intake responds to a change in fruit juice and milk prices; cross-price elasticity estimates for both goods indicated a strong substitute relationship, particularly for fruit juice. Based on economic theory, we can infer the relationship between fruit juice or milk intake and SSB prices from our cross-price estimates (see supplemental table 5); however, this would require knowledge about SSB expenditures relative to fruit juice and milk (see supplementary information, technical appendix).(32) Based on our results, SSB taxes would lead to an increase in both fruit juice and milk intake, but fruit juice intake would increase by a relatively larger percent.

Strengths and limitations

This study has several strengths, the first being the extensive country coverage. We provide a global snapshot of SSB intake behavior allowing for comparisons within and across most countries. Since past studies have been limited to a single country or a select group of countries, the results of this study informs policy and decision-making beyond the current state of knowledge. Problems associated with poor diets and NCDs occur in both developing and

For peer review only

developed countries.(33) A comparative analysis across the complete spectrum of countries can assist international organizations in developing heterogeneous strategies for specific subgroups and countries. Our use of individual intakes by age, sex, and country provides for more accurate representation of dietary behavior. Previous findings based on expenditure data may be limited by differences in expenditures and actual consumption.

Potential limitations should also be considered. First, being a modeling study, the projected outcomes can only inform how taxes could affect behavior. While an intervention study would be more fitting, interventions across 164 countries would not be feasible. Secondly, our analysis was limited by the use of price and income data at the national level. Ideally, our explanatory variables would also be at the subgroup level, reflecting that incomes typically vary with age and sex, and different subgroups could face a different set of prices within a country. For instance, in countries where urban populations are relatively young, young adults could face different prices depending on market conditions in urban and rural areas. This limitation is due to the number of countries in our study. Such detailed data is not available for many countries.

We did not have price data on SSBs; using sugar prices as a proxy might underestimate the magnitude of own-price elasticities. For instance, according to the U.S. Bureau of Labor Statistics data, the producer price index for sugar has increased by 61% since 2000, while the consumer price index for carbonated beverages increased by 30% over the same period. In this instance, it could be argued that the own-price elasticities for the U.S. and comparable countries should be increased by a factor of two. In preliminary analysis, we found an almost perfect correlation between the U.S. sugar and carbonated beverage price indices, providing some evidence that the sugar price index is an acceptable proxy.

For peer review only - http://bmjopen.bmj.com/site/about/guidelines.xhtml

The regional variation in own-price elasticities (see figure 2) is due, in part, to differences in intake levels across countries. For instance, the lower own-price responsiveness for LAC is due to high intake in that region. Note that elasticities derived from a semi-log functional form decrease (in absolute value) with rising intake (equation 3, supplementary information, technical appendix). While this is plausible within a certain intake range, particularly high intake could result in unusually low own-price responsiveness. Consequently, the small own-price elasticities for LAC may not be representative of individual behavior in the region. For this reason, the elasticities reported across countries by income decile, which are based on median intake levels for each demographic subgroup, are likely more representative of actual global behavior.

Comparison with other studies

Since previous research has mostly focused on higher income countries, primarily the U.S., it is difficult to compare all of our results with earlier findings. Several U.S. based studies have considered how SSB consumption would respond to a tax. Given a 10% tax, the projected decrease in SSB intake ranged from 6.7% to 18.2%.(14) These results are greater than our findings for countries in the highest income decile (0%, women, age ≥ 60 to 6.1%, men, age < 35) due to our relatively smaller own-price elasticities. Whereas our own-price elasticity estimates for the highest income countries range from -0.3 to -0.0, meta-analyses of U.S. studies give estimates of -0.8 (-3.2 to -0.13) and -1.1 (-1.3 to -0.9).(15, 34) In a study of Mexico using data before and after implementation of a national soda tax (10%), SSB purchases decreased by an average of 6% during the first year of implementation, (11) which is closer, but still larger than our findings for middle-income countries.

For peer review only - http://bmjopen.bmj.com/site/about/guidelines.xhtml

The fact that our estimates are relative smaller does not necessarily make them less accurate. Note that past studies have mostly used expenditure data. Since SSBs are storable goods, individuals can take advantage of price discounts, increasing expenditures when prices are low, stock piling for future consumption. Ignoring this fact can result in overestimates of own-price elasticities, and hence, tax policy outcomes.(35)

Conclusion

This is the first study to examine SSB consumption and taxation in a global context. Our findings provide a better understanding of the potential effectiveness of taxes across the full spectrum of countries. Overall, we found that the influence of SSB prices on intake significantly depends on the income status of countries, from which we could also infer the behavior of lower income individuals. Our results suggest that intake reductions (in percent) could be small or negligible for certain demographics in higher income countries. Although small in percentage terms, actual intake reductions could still be sizeable enough for high-consuming subgroups for taxes to be worth pursuing. For higher income countries, a larger tax or a tax combined with other approaches might be needed to significantly change behavior. For instance, taxes could be combined with media and education campaigns, food labeling, and other interventions.(36) For lower income countries, our findings indicate that taxes would be particularly effective, which is to be expected since food expenditures account for a greater share of income and low-income individuals are expected to be more sensitive to prices.

For peer review only

For peer review only - http://bmjopen.bmj.com/site/about/guidelines.xhtml

References

1. de Koning L, Malik VS, Kellogg MD, Rimm EB, Willett WC, Hu FB. Sweetened beverage consumption, incident coronary heart disease, and biomarkers of risk in men. Circulation. 2012;125(14):1735-41.

2. Fung TT, Malik V, Rexrode KM, Manson JE, Willett WC, Hu FB. Sweetened beverage consumption and risk of coronary heart disease in women. The American Journal of Clinical Nutrition. 2009;89(4):1037-42.

3. Ludwig DS, Peterson KE, Gortmaker SL. Relation between consumption of sugar-sweetened drinks and childhood obesity: a prospective, observational analysis. Lancet. 2001;357(9255):505-8.

4. Malik VS, Popkin BM, Bray GA, Després J-P, Willett WC, Hu FB. Sugar-sweetened beverages and risk of metabolic syndrome and type 2 diabetes. Diabetes Care. 2010;33(11):2477-83.

5. Mozaffarian D, Hao T, Rimm EB, Willett WC, Hu FB. Changes in diet and lifestyle and long-term weight gain in women and men. N Engl J Med. 2011;364(25):2392-404.

6. Eyles H, Ni Mhurchu C, Nghiem N, Blakely T. Food pricing strategies, population diets, and non-communicable disease: a systematic review of simulation studies. PLoS Med. 2012;9(12):e1001353.

7. World Health Organization. Using Price Policies to Promote Healthier Diets. Copenhagen, Denmark: WHO Regional Office for Europe; 2015.

8. Gustin G. Latest Soda Tax Puts Pressure on Waistlines and the World. National Geographic. 2016 March 17, 2016.

9. Grogger J. Soda Taxes and the Prices of Sodas and Other Drinks: Evidence from Mexico. National Bureau of Economic Research; 2015. Contract No.: NBER Working Paper No. 21197.

10. Paarlberg R, Mozaffarian D, Micha R. Viewpoint: Can U.S. local soda taxes continue to spread? Food Pol. 2017;71:1-7.

11. Colchero MA, Popkin BM, Rivera JA, Ng SW. Beverage Purchases from Stores in Mexico Under the Excise Tax on Sugar Sweetened Beverages: Observational Study. BMJ. 2016;352:h6704.

12. Brownell KD, Farley T, Willett WC, Popkin BM, Chaloupka FJ, Thompson JW, et al. The public health and economic benefits of taxing sugar-sweetened beverages. N Engl J Med. 2009;361(16):1599-605.

13. Nakhimovsky SS, Feigl AB, Avila C, O'Sullivan G, Macgregor-Skinner E, Spranca M. Taxes on sugar-sweetened beverages to reduce overweight and obesity in middle-income countries: a systematic review. PLoS One. 2016;11(9):e0163358.

For peer review only

For peer review only - http://bmjopen.bmj.com/site/about/guidelines.xhtml

14. Afshin A, Peñalvo JL, Del Gobbo L, Silva J, Michaelson M, O'Flaherty M, et al. The prospective impact of food pricing on improving dietary consumption: A systematic review and meta-analysis. PLoS One. 2017;12(3):e0172277.

15. Powell LM, Chriqui JF, Khan T, Wada R, Chaloupka FJ. Assessing the potential effectiveness of food and beverage taxes and subsidies for improving public health: a systematic review of prices, demand and body weight outcomes. Obes Rev. 2013;14(2):110-28.

16. Briggs ADM, Mytton OT, Kehlbacher A, Tiffin R, Rayner M, Scarborough P. Overall and income specific effect on prevalence of overweight and obesity of 20% sugar sweetened drink tax in UK: econometric and comparative risk assessment modelling study. BMJ. 2013;347:f6189.

17. Grogger J. Soda taxes and the prices of sodas and other drinks: evidence from Mexico. Amer J Agr Econ. 2017;99(2):481-98.

18. World Health Organization. Fiscal Policies for Diet and the Prevention of Noncommunicable Diseases. Technical Meeting Report. Geneva, Switzerland: World Health Organization; 2016.

19. Mytton OT, Clarke D, Rayner M. Taxing unhealthy food and drinks to improve health. BMJ. 2012;344(7857):e2931.

20. Del Gobbo LC, Khatibzadeh S, Imamura F, Micha R, Shi P, Smith M, et al. Assessing global dietary habits: a comparison of national estimates from the FAO and the Global Dietary Database. Am J Clin Nutr. 2015;101(5):1038-46.

21. Imamura F, Micha R, Khatibzadeh S, Fahimi S, Shi P, Powles J, et al. Dietary quality among men and women in 187 countries in 1990 and 2010: a systematic assessment. Lancet Global Health. 2015;3(3):e132-e42.

22. Micha R, Kalantarian S, Wirojratana P, Byers T, Danaei G, Elmadfa I, et al. Estimating the global and regional burden of suboptimal nutrition on chronic disease: methods and inputs to the analysis. Eur J Clin Nutr. 2012;66(1):119-29.

23. Micha R, Khatibzadeh S, Shi P, Fahimi S, Lim S, Andrews KG, et al. Global, regional, and national consumption levels of dietary fats and oils in 1990 and 2010: a systematic analysis including 266 country-specific nutrition surveys. BMJ. 2014;348:g2272.

24. Khatibzadeh S, Kashaf MS, Micha R, Fahimi S, Shi P, Elmadfa I, et al. A global database of food and nutrient consumption. Bull World Health Organ. 2016;94(12):931-4E.

25. World Bank. Purchasing Power Parities and Real Expenditures of World Economies Washington, D.C.: World Bank Publications; 2014.

26. World Bank. Purchasing Power Parities and the Real Size of World Economies Washington, D.C.: World Bank Publications; 2015.

For peer review only

27. World Bank. World Development Indicators 2015 [Available from: http://data.worldbank.org/data-catalog/world-development-indicators.

28. LaFrance JT. Incomplete demand systems and semilogarithmic demand models. Australian J Agr Econ. 1990;34(2):118-31.

29. von Haefen RH. A complete characterization of the linear, log-linear, and semi-log incomplete demand system models. J Agr Resource Econ. 2002;27(2):281-319.

30. Cameron AC, Trivedi PK. Microeconometrics: Methods and Applications. Cambridge: Cambridge University Press; 2005.

31. Singh GM, Micha R, Khatibzadeh S, Shi P, Lim S, Andrews KG, et al. Global, regional, and national consumption of sugar-sweetened beverages, fruit juices, and milk: a systematic assessment of beverage intake in 187 countries. PLoS One. 2015;10(8):e0124845.

32. Tomek WG, Robinson KL. Agricultural Product Prices. Ithaca, New York: Cornell University Press; 1990.

33. United Nations. Note by the Secretary-General transmitting the report of the Director-General of the World Health Organization on the prevention and control of non-communicable diseases. New York: United Nations; 2013. Report No.: A/68/650 Contract No.: A/68/650.

34. Andreyeva T, Long MW, Brownell KD. The impact of food prices on consumption: a systematic review of research on the price elasticity of demand for food. Am J Public Health. 2010;100(2):216-22.

35. Hartmann WR. Intertemporal effects of consumption and their implications for demand elasticity estimates. Quantitative Marketing and Economics. 2006;4(4):325-49.

36. Afshin A, Micha R, Khatibzadeh S, Schmidt LA, Mozaffarian D. Dietary Policies to Reduce Noncommunicable Diseases. In: Brown GW, Yamey G, Wamala S, editors. The Handbook of Global Health Policy. West Sussex, UK: Wiley-Blackwell; 2014. p. 175-93.

For peer review only

For peer review only - http://bmjopen.bmj.com/site/about/guidelines.xhtml

Figure 1 Comparison of mean SSB intakes among adults in age (5-year interval), sex, and country-specific strata across world regions and globally by select age groups. The n's represent the number of age, sex, and country-specific subgroups in each stratum. Boxes represent the median intake value and interquartile range; error bars represent the minimum and maximum values. Source: Global Dietary Database, 2010

Figure 2 Comparison of SSB own-price elasticities across regions. The n's represent the number of age, sex, and country-specific subgroups in each stratum. Points are elasticity values for each subgroup. Boxes represent the median value and interquartile range; error bars represent the minimum and maximum values. Own-price elasticities are based on 1% price changes. For instance, given a 1% SSB price increase in Central Asia, intake by women falls by 0.74% (median response).

Figure 3 Median global SSB own-price elasticities by age, sex, and global income decile. Values are derived at median intake levels by demographic subgroup. Own-price elasticities are based on 1% price changes. For instance, given a 1% SSB price increase in the lowest income countries, intake by women, age 20 falls by 0.58%. Income deciles are based on the national income of the 164 countries included in the study. Each decile is composed of 16 countries (except the 4 lowest deciles, which are each composed of 17 countries). The per capita income range (PPP-adjusted in thousand $US) for each decile: (1st) $0.6-$1.5, (2nd) $1.5-$2.7, (3rd) $2.7-$5.3, (4th) $5.5-$8.0, (5th) $8.3-$10.8, (6th) $11.1-$15.2, (7th) $15.3-$20.3, (8th) $20.6-$29.4, (9th) $30.4-$40.9, and (10th) $41.3-$127.2.

For peer review only

For peer review only - http://bmjopen.bmj.com/site/about/guidelines.xhtml

Table 1 Own-price elasticities of SSB intake by age, sex, and global income decile†

Income decile‡	Age 20	Age 30	Age 40	Age 50	Age 60	Age 70	Age 80
	Women						
Lowest 10%	-0.58 (0.22)***	-0.62 (0.25)**	-0.73 (0.33)**	-0.76 (0.39)**	-0.80 (0.44)*	-0.84 (0.49)*	-0.96 (0.55)*
2nd	-0.53 (0.20)***	-0.56 (0.22)**	-0.64 (0.28)**	-0.65 (0.31)**	-0.66 (0.35)*	-0.68 (0.38)*	-0.79 (0.43)*
3rd	-0.49 (0.18)***	-0.49 (0.19)***	-0.55 (0.22)**	-0.53 (0.24)**	-0.52 (0.26)**	-0.52 (0.28)*	-0.61 (0.32)*
4th	-0.45 (0.16)***	-0.44 (0.17)***	-0.47 (0.19)**	-0.43 (0.19)**	-0.39 (0.19)**	-0.39 (0.20)*	-0.46 (0.23)**
5th	-0.42 (0.15)***	-0.41 (0.15)***	-0.42 (0.17)**	-0.37 (0.16)**	-0.31 (0.16)**	-0.30 (0.16)*	-0.36 (0.19)*
6th	-0.40 (0.15)***	-0.38 (0.15)***	-0.38 (0.16)**	-0.31 (0.15)**	-0.25 (0.14)*	-0.22 (0.14)	-0.28 (0.16)*
7th	-0.38 (0.15)***	-0.35 (0.14)**	-0.34 (0.15)**	-0.26 (0.14)*	-0.19 (0.13)	-0.15 (0.13)	-0.20 (0.16)
8th	-0.36 (0.14)**	-0.32 (0.14)**	-0.29 (0.15)*	-0.20 (0.15)	-0.11 (0.15)	-0.07 (0.15)	-0.11 (0.17)
9th	-0.33 (0.14)**	-0.28 (0.14)**	-0.23 (0.16)	-0.13 (0.17)	-0.03 (0.18)	0.03 (0.19)	0.00 (0.22)
Highest 10%	-0.29 (0.15)**	-0.23 (0.15)	-0.16 (0.19)	-0.03 (0.21)	0.09 (0.24)	0.16 (0.26)	0.14 (0.29)
	Men						
Lowest 10%	-0.58 (0.20)***	-0.62 (0.23)***	-0.78 (0.31)**	-0.87 (0.37)**	-0.92 (0.42)**	-0.98 (0.46)**	-1.10 (0.51)**
2nd	-0.54 (0.18)***	-0.57 (0.20)***	-0.70 (0.26)***	-0.76 (0.30)**	-0.79 (0.33)**	-0.83 (0.36)**	-0.94 (0.40)**
3rd	-0.50 (0.16)***	-0.51 (0.17)***	-0.62 (0.21)***	-0.65 (0.23)***	-0.66 (0.25)***	-0.69 (0.27)***	-0.78 (0.30)***
4th	-0.46 (0.15)***	-0.47 (0.15)***	-0.54 (0.18)***	-0.55 (0.19)***	-0.55 (0.19)***	-0.56 (0.19)***	-0.64 (0.22)***
5th	-0.44 (0.14)***	-0.44 (0.14)***	-0.50 (0.16)***	-0.49 (0.16)***	-0.47 (0.16)***	-0.48 (0.16)***	-0.55 (0.18)***
6th	-0.42 (0.14)***	-0.41 (0.13)***	-0.46 (0.15)***	-0.44 (0.15)***	-0.41 (0.14)***	-0.41 (0.14)***	-0.48 (0.16)***
7th	-0.40 (0.13)***	-0.39 (0.13)***	-0.42 (0.15)***	-0.39 (0.14)***	-0.36 (0.13)***	-0.34 (0.13)***	-0.41 (0.15)***
8th	-0.38 (0.13)***	-0.36 (0.13)***	-0.38 (0.15)***	-0.34 (0.15)**	-0.29 (0.14)**	-0.26 (0.15)*	-0.32 (0.16)*
9th	-0.35 (0.13)***	-0.32 (0.13)**	-0.33 (0.15)**	-0.27 (0.16)*	-0.21 (0.17)	-0.18 (0.18)	-0.22 (0.20)
Highest 10%	-0.32 (0.14)**	-0.28 (0.14)**	-0.26 (0.17)	-0.18 (0.20)	-0.10 (0.23)	-0.06 (0.25)	-0.09 (0.27)

Values are derived at median intake levels by demographic subgroup (standard errors). *p≤0.10; **p≤0.05; ***p≤0.01.
†Price elasticities are based on 1% price changes. For instance, given a 1% SSB price increase in the lowest income countries, intake by women, age 20 falls by 0.58%.
‡Income deciles are based on the national income of the 164 countries included in the study. Each decile is composed of 16 countries (except the 4 lowest deciles, which are each composed of 17 countries). The per capita income range (PPP-adjusted in thousand $US) for each decile: (1st) $0.6-$1.5, (2nd) $1.5-$2.7, (3rd) $2.7-$5.3, (4th) $5.5-$8.0, (5th) $8.3-$10.8, (6th) $11.1-$15.2, (7th) $15.3-$20.3, (8th) $20.6-$29.4, (9th) $30.4-$40.9, and (10th) $41.3-$127.2.

For peer review only - http://bmjopen.bmj.com/site/about/guidelines.xhtml

Table 2 Potential impact of a 20% tax (price increase) on SSB intake by age, sex, and global income decile.

Income decile†	Women age < 35	Men age < 35	Women 35 ≤ age < 60	Men 35 ≤ age < 60	Women age ≥ 60	Men age ≥ 60
	% reduction in intake (95% CI)					
Lowest 10%	12.0 (2.7 to 21.4)	12.1 (3.7 to 20.6)	14.9 (1.0 to 28.9)	16.1 (3.2 to 29.0)	16.8 (-2.5 to 35.9)	19.6 (1.6 to 37.6)
2nd	11.0 (2.8 to 19.2)	11.2 (3.7 to 18.7)	12.9 (1.4 to 24.4)	14.3 (3.7 to 24.9)	13.7 (-1.4 to 28.6)	16.8 (2.5 to 30.9)
3rd	9.9 (2.7 to 17.1)	10.2 (3.6 to 16.9)	10.8 (1.7 to 20.0)	12.4 (3.9 to 20.9)	10.5 (-0.6 to 21.5)	13.8 (3.3 to 24.3)
4th	9.0 (2.5 to 15.5)	9.4 (3.4 to 15.4)	9.0 (1.7 to 16.4)	10.8 (3.8 to 17.7)	7.7 (-0.2 to 15.6)	11.2 (3.6 to 18.9)
5th	8.4 (2.3 to 14.5)	8.8 (3.2 to 14.5)	7.8 (1.4 to 14.3)	9.7 (3.5 to 15.9)	5.9 (-0.3 to 12.2)	9.6 (3.5 to 15.7)
6th	7.9 (2.0 to 13.7)	8.4 (3.0 to 13.8)	6.9 (1.0 to 12.9)	8.9 (3.1 to 14.6)	4.5 (-0.9 to 9.9)	8.2 (2.9 to 13.6)
7th	7.4 (1.7 to 13.1)	8.0 (2.7 to 13.3)	6.0 (0.3 to 11.7)	8.0 (2.5 to 13.6)	3.1 (-2.1 to 8.2)	6.9 (1.8 to 12.1)
8th	6.8 (1.2 to 12.4)	7.5 (2.3 to 12.6)	4.9 (-1.0 to 10.7)	7.0 (1.4 to 12.6)	1.3 (-4.6 to 7.3)	5.3 (-0.4 to 11.0)
9th	6.2 (0.5 to 11.8)	6.9 (1.7 to 12.1)	3.6 (-2.8 to 10.0)	5.9 (-0.2 to 11.9)	-0.6 (-8.1 to 7.0)	3.6 (-3.6 to 10.7)
Highest 10%	5.3 (-0.6 to 11.2)	6.1 (0.7 to 11.5)	2.0 (-5.7 to 9.6)	4.4 (-2.8 to 11.5)	-3.1 (-13.5 to 7.3)	1.2 (-8.5 to 10.9)

Values are reductions from median intake reduction for each demographic subgroup.
†Income deciles are based on the national income of the 164 countries included in the study. Each decile is composed of 16 countries except the 4 lowest deciles, which are each composed of 17 countries. The per capita income range (PPP-adjusted in thousand $US) for each decile: (1st) $0.6-$1.5, (2nd) $1.5-$2.7, (3rd) $2.7-$5.3, (4th) $5.5-$8.0, (5th) $8.3-$10.8, (6th) $11.1-$15.2, (7th) $15.3-$20.3, (8th) $20.6-$29.4, (9th) $30.4-$40.9, and (10th) $41.3-$127.2.

For peer review only

<Insert Figure1.png file here>

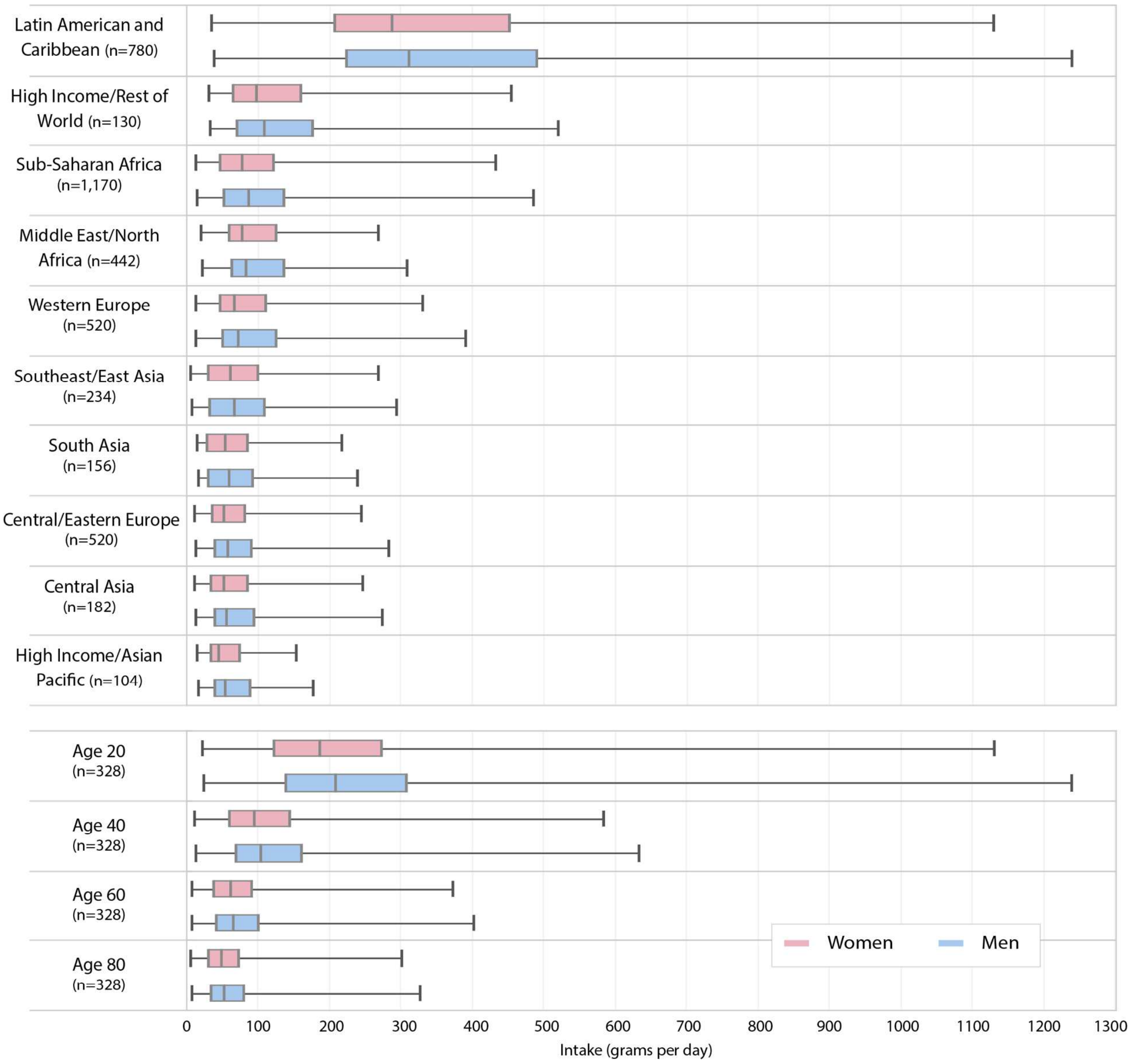

Figure 1 Comparison of mean SSB intakes among adults in age (5-year interval), sex, and country-specific strata across world regions and globally by select age groups. The n's represent the number of age, sex, and country-specific subgroups in each stratum. Boxes represent the median intake value and interquartile range; error bars represent the minimum and maximum values. Source: Global Dietary Database, 2010

For peer review only - http://bmjopen.bmj.com/site/about/guidelines.xhtml

<Insert Figure2.png file here>

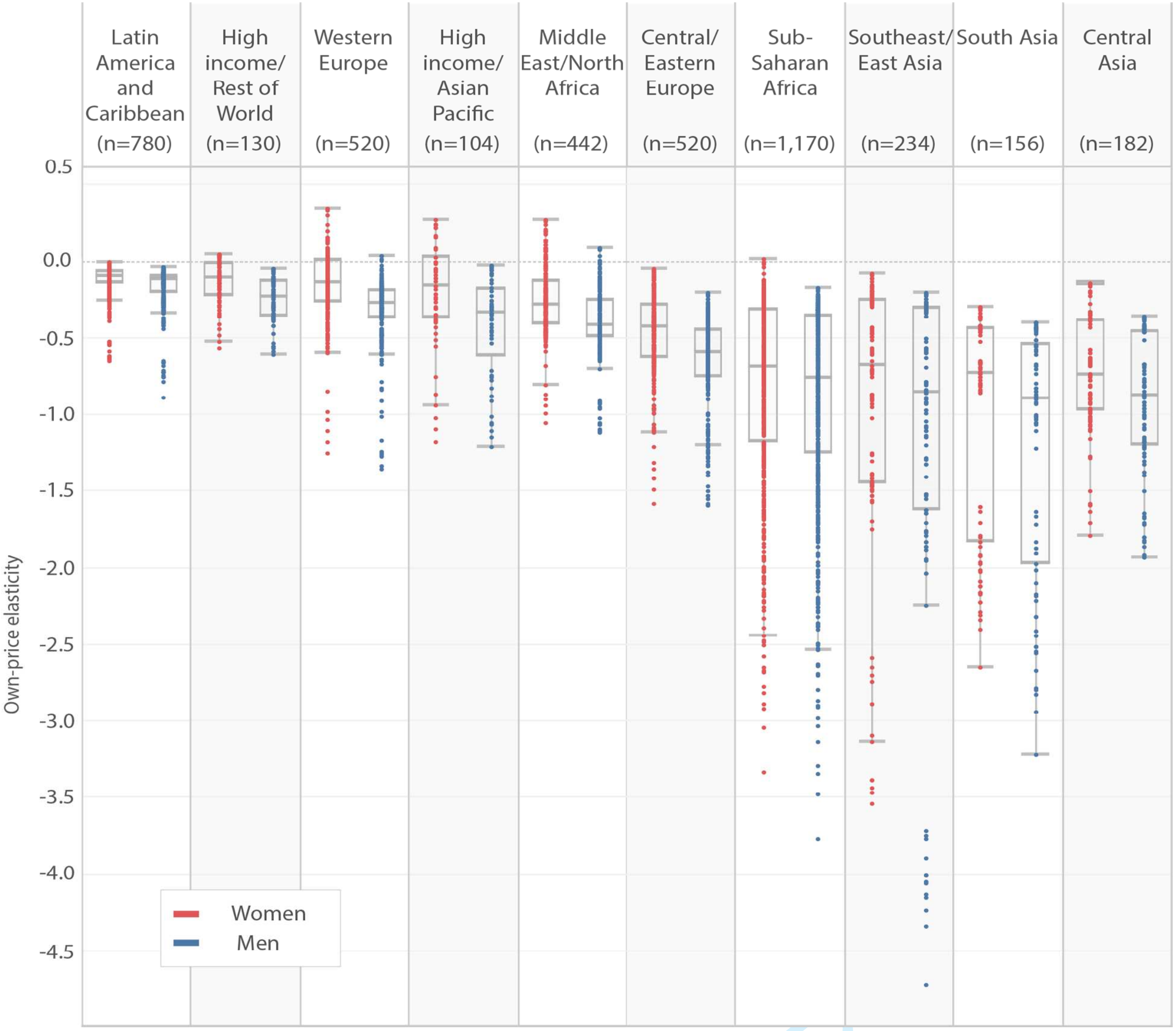

Figure 2 Comparison of SSB own-price elasticities across regions. The n's represent the number of age, sex, and country-specific subgroups in each stratum. Points are elasticity values for each subgroup. Boxes represent the median value and interquartile range; error bars represent the minimum and maximum values. Own-price elasticities are based on 1% price changes. For instance, given a 1% SSB price increase in Central Asia, intake by women falls by 0.74% (median response).

<Insert Figure3.png file here>

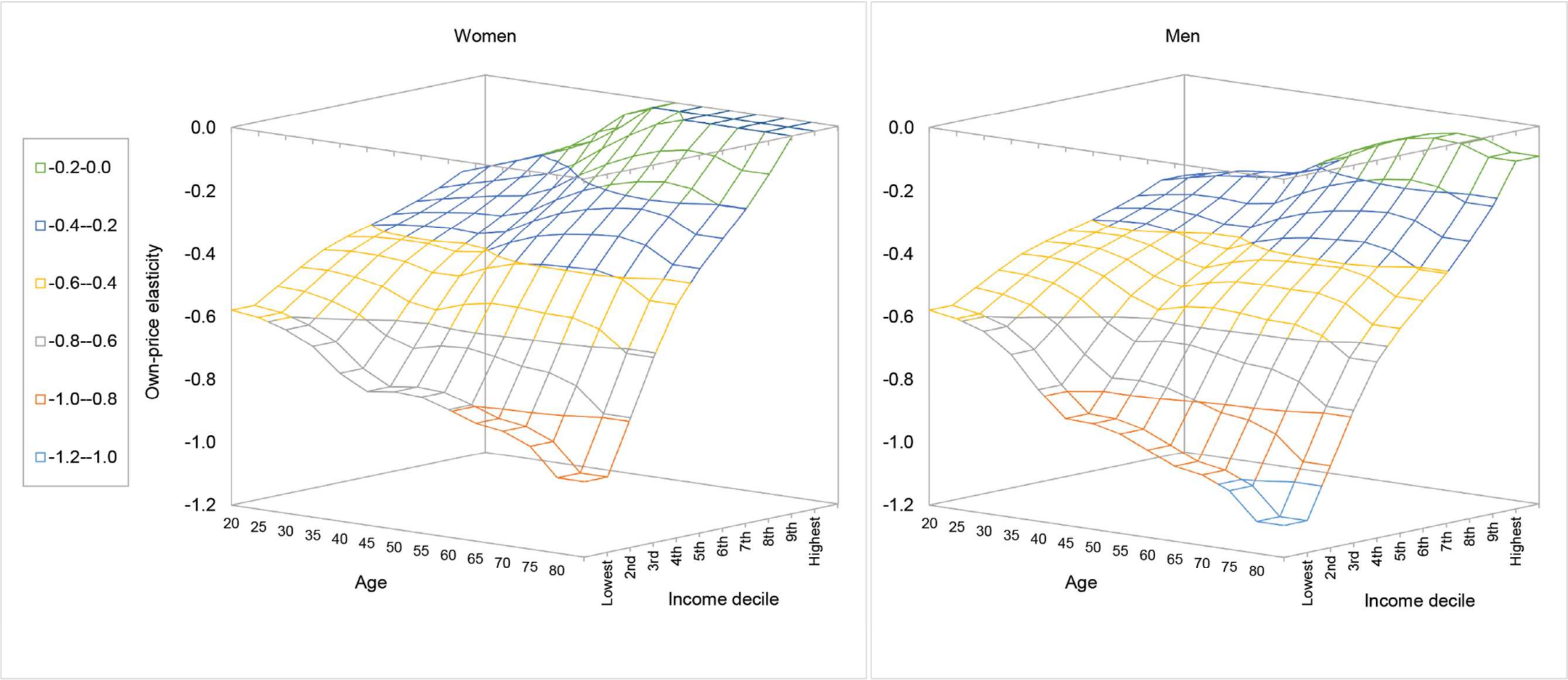

Figure 3 Median global SSB own-price elasticities by age, sex, and global income decile. Values are derived at median intake levels by demographic subgroup. Own-price elasticities are based on 1% price changes. For instance, given a 1% SSB price increase in the lowest income countries, intake by women, age 20 falls by 0.58%. Income deciles are based on the national income of the 164 countries included in the study. Each decile is composed of 16 countries (except the 4 lowest deciles, which are each composed of 17 countries). The per capita income range (PPP-adjusted in thousand $US) for each decile: (1st) $0.6-$1.5, (2nd) $1.5-$2.7, (3rd) $2.7-$5.3, (4th) $5.5-$8.0, (5th) $8.3-$10.8, (6th) $11.1-$15.2, (7th) $15.3-$20.3, (8th) $20.6-$29.4, (9th) $30.4-$40.9, and (10th) $41.3-$127.2.

For peer review only - http://bmjopen.bmj.com/site/about/guidelines.xhtml

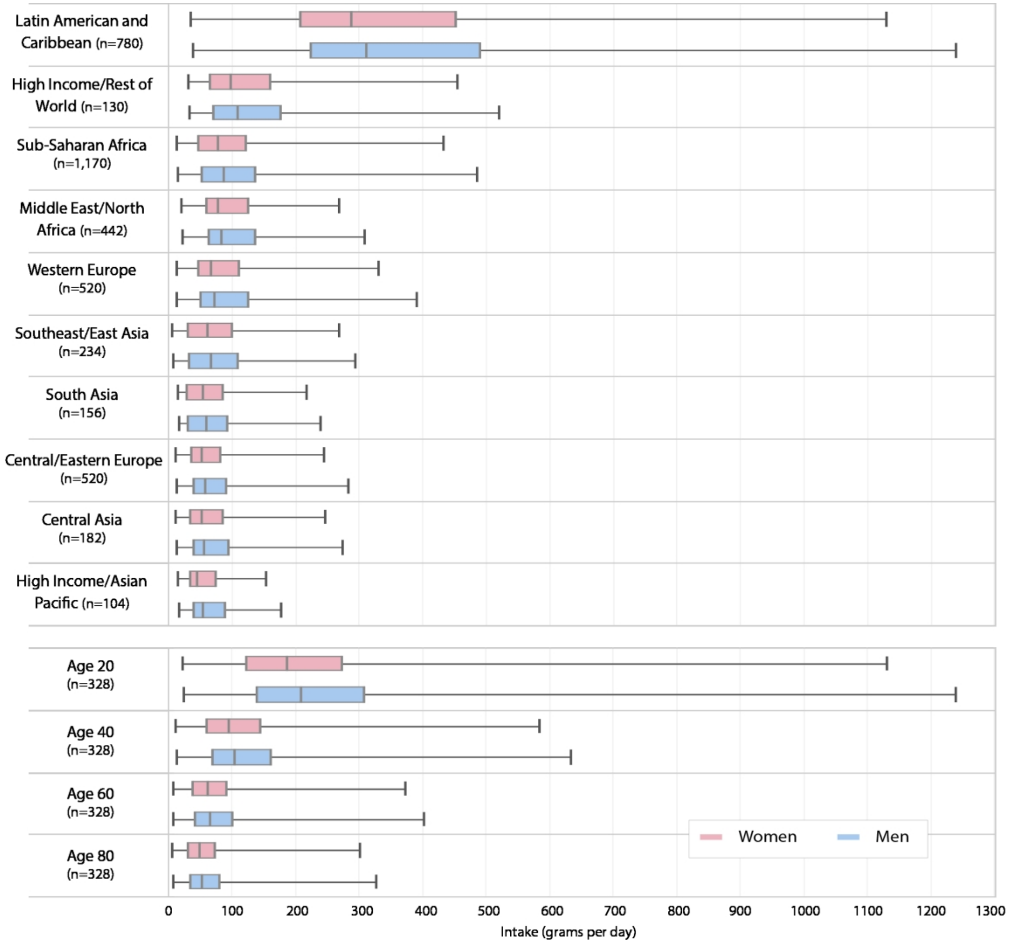

Figure 1 Comparison of mean SSB intakes among adults in age (5-year interval), sex, and country-specific strata across world regions and globally by select age groups. The n's represent the number of age, sex, and country-specific subgroups in each stratum. Boxes represent the median intake value and interquartile range; error bars represent the minimum and maximum values. Source: Global Dietary Database, 2010

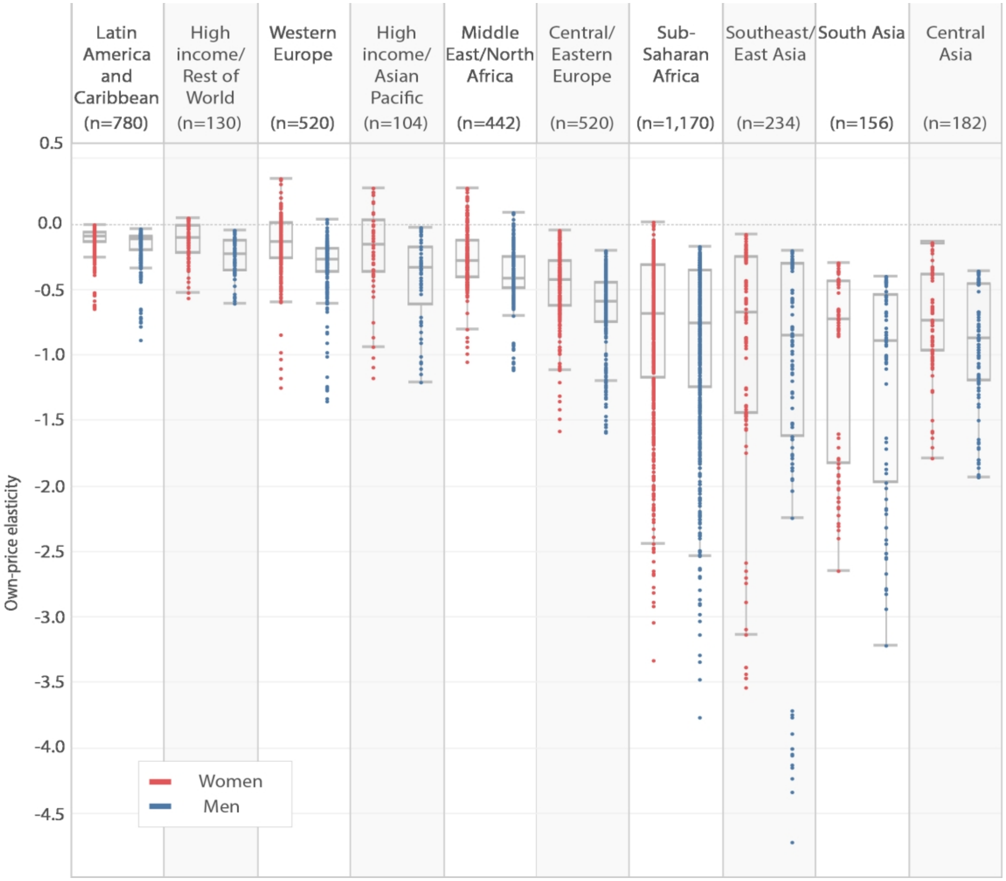

Figure 2 Comparison of SSB own-price elasticities across regions. The n's represent the number of age, sex, and country-specific subgroups in each stratum. Points are elasticity values for each subgroup. Boxes represent the median value and interquartile range; error bars represent the minimum and maximum values. Own-price elasticities are based on 1% price changes. For instance, given a 1% SSB price increase in Central Asia, intake by women falls by 0.74% (median response).

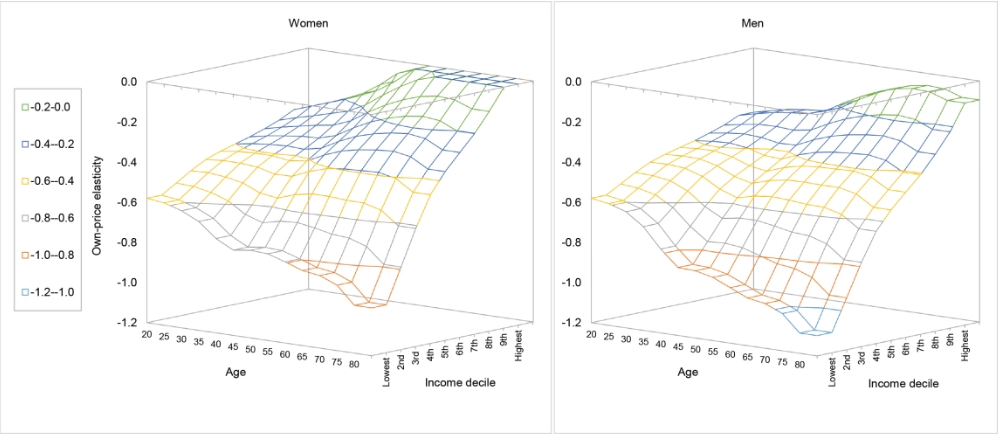

Figure 3 Median global SSB own-price elasticities by age, sex, and global income decile. Values are derived at median intake levels by demographic subgroup. Own-price elasticities are based on 1% price changes. For instance, given a 1% SSB price increase in the lowest income countries, intake by women, age 20 falls by 0.58%. Income deciles are based on the national income of the 164 countries included in the study. Each decile is composed of 16 countries (except the 4 lowest deciles, which are each composed of 17 countries). The per capita income range (PPP-adjusted in thousand $US) for each decile: (1st) $0.6-$1.5, (2nd) $1.5-$2.7, (3rd) $2.7-$5.3, (4th) $5.5-$8.0, (5th) $8.3-$10.8, (6th) $11.1-$15.2, (7th) $15.3-$20.3, (8th) $20.6-$29.4, (9th) $30.4-$40.9, and (10th) $41.3-$127.2.

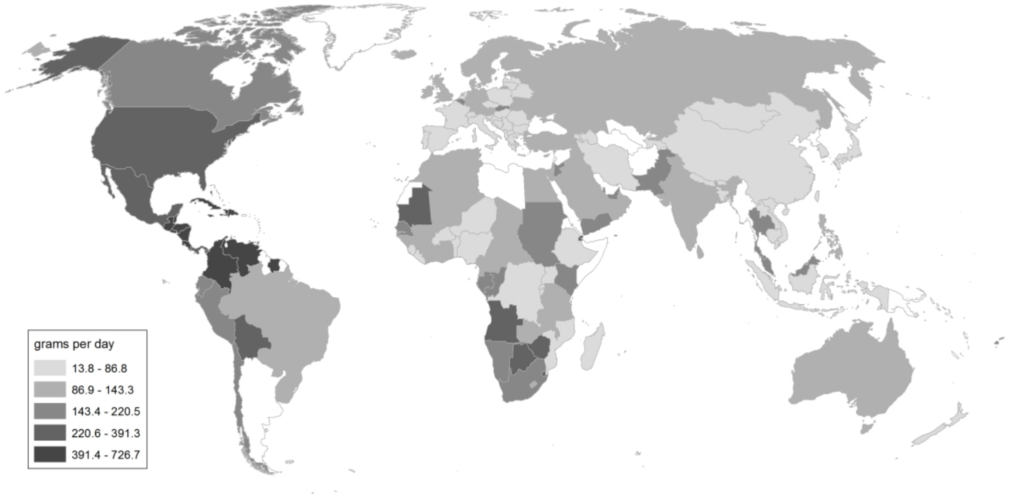

Supplemental Figure 1
Mean SSB intake across countries in adults, age ≥ 20
Source: Global Dietary Database, 2010

Global patterns in price elasticities of sugar-sweetened beverage intake and potential effectiveness of tax policy: results from 164 countries

Andrew Muhammad,[1] Birgit Meade,[2] David R Marquardt,[2] and Dariush Mozaffarian[3]

[1]University of Tennessee Institute of Agriculture, Department of Agricultural and Resource Economics, Knoxville, Tennessee, USA

[2]United States Department of Agriculture, Economic Research Service, Market and Trade

Economics Division, Washington, D.C., USA

[3]Tufts University, Friedman School of Nutrition Science and Policy, Boston, Massachusetts,

USA

Supplemental Figure 1	Mean SSB intake across countries in adults, age ≥ 20
Supplemental Table 1	Description of ICP food price categories
Supplemental Table 2	Countries included in the study by region (aggregate regions used for estimation)
Supplemental Table 3	Demand model estimates for SSB intake
Supplemental Table 4	Own-price elasticities of SSB intake by age, sex, and global income decile
Supplemental Table 5	Cross-price elasticities of SSB intake with respect to changes in fruit juice and milk prices, by age and sex
Technical Appendix	Cross-country intake demand model and elasticities

For peer review only - http://bmjopen.bmj.com/site/about/guidelines.xhtml

Supplementary Information

<Insert sup_fig1.png file here.>

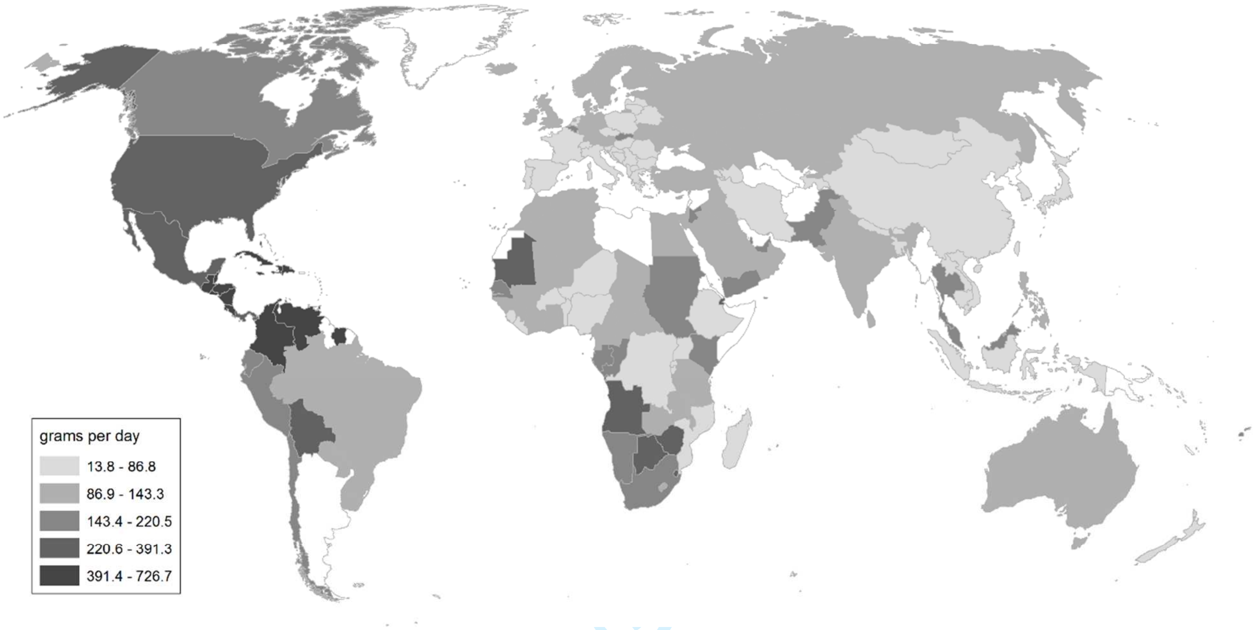

Supplemental Figure 1

Mean SSB intake across countries in adults, age ≥ 20

Source: Global Dietary Database, 2010

For peer review only - http://bmjopen.bmj.com/site/about/guidelines.xhtml

Supplemental Table 1

Description of ICP food price categories

ICP food price category
• *Fresh or chilled fruit* – All fresh or chilled fruit including melons and water melons; excludes vegetables grown for their fruit such as cucumbers and tomatoes.
• *Fresh milk* – Raw milk; pasteurised or sterilised milk; includes whole and low fat milk; recombined or reconstituted milk; soya milk.
• *Sugar* – Cane or beet sugar, unrefined or refined, powdered, crystallised or in lumps; includes artificial sugar substitutes.

For peer review only

Supplemental Table 2

Countries included in the study by region (aggregate regions used for estimation)

Region	Countries
Southeast Asia, East Asia, and High Income Asia Pacific (Asia) (13 countries)	Brunei Darussalam, Cambodia, China, Indonesia, Japan, South Korea, Laos, Malaysia, Maldives, Philippines, Singapore, Thailand, and Vietnam.
Central Europe, Eastern Europe, and Central Asia (CEE/C. Asia) (27 countries)	Albania, Armenia, Azerbaijan, Belarus, Bosnia and Herzegovina, Bulgaria, Croatia, Czech Republic, Estonia, Georgia, Hungary, Kazakhstan, Kyrgyz Republic, Latvia, Lithuania, Macedonia, Moldova, Mongolia, Montenegro, Poland, Romania, Russia, Serbia, Slovak Republic, Slovenia, Tajikistan, and Ukraine.
Latin America and the Caribbean (LAC) (30 countries)	Antigua and Barbuda, Bahamas, Barbados, Belize, Bolivia, Brazil, Chile, Colombia, Costa Rica, Cuba, Dominica, Dominican Republic, Ecuador, El Salvador, Grenada, Guatemala, Haiti, Honduras, Jamaica, Mexico, Nicaragua, Panama, Paraguay, Peru, St. Lucia, St. Vincent and Grenadines, Suriname, Trinidad and Tobago, Uruguay, and Venezuela.
Middle East, North Africa, and South Asia (MENA/S. Asia) (23 countries)	Algeria, Bahrain, Bangladesh, Bhutan, Egypt, India, Iran, Iraq, Israel, Jordan, Kuwait, Morocco, Nepal, Oman, Pakistan, Qatar, Saudi Arabia, Sri Lanka, Tunisia, Turkey, United Arab Emirates, West Bank and Gaza, and Yemen.
Sub-Saharan Africa (SSA) (45 countries)	Angola, Benin, Botswana, Burkina Faso, Burundi, Cameroon, Cape Verde, Central African Republic, Chad, Comoros, Congo, Côte d'Ivoire, Djibouti, DR Congo, Equatorial Guinea, Ethiopia, Gabon, Gambia, Ghana, Guinea, Guinea-Bissau, Kenya, Lesotho, Liberia, Madagascar, Malawi, Mali, Mauritania, Mauritius, Mozambique, Namibia, Niger, Nigeria, Rwanda, São Tomé and Principe, Senegal, Sierra Leone, South Africa, Sudan, Swaziland, Tanzania, Togo, Uganda, Zambia, and Zimbabwe.
High Income/Rest of World (HIC) (26 countries)	Australia, Austria, Belgium, Canada, Cyprus, Denmark, Fiji, Finland, France, Germany, Greece, Iceland, Ireland, Italy, Luxembourg, Malta, Netherlands, New Zealand, Norway, Portugal, Seychelles, Spain, Sweden, Switzerland, United Kingdom, and United States.

For peer review only

Supplemental Table 3

Demand model estimates for SSB intake

Variable	model 1 estimate	model 1 (SE)	model 2 estimate	model 2 (SE)	model 3 (final model) estimate	model 3 (final model) (SE)
constant	436.63	(25.47)***	312.26	(57.27)***	-544.53	(261.08)**
female (F)	-13.36	(0.82)***	-13.36	(0.82)***	-17.10	(7.15)**
age	-10.87	(0.74)***	-10.87	(0.74)***	0.66	(4.39)
age^2	0.08	(0.01)***	0.08	(0.01)***	0.00	(0.03)
SSA	1.83	(11.47)	40.89	(17.16)**	104.71	(57.98)*
LAC	258.41	(26.38)***	265.62	(27.07)***	812.10	(91.78)***
MENA/S. Asia	-10.05	(10.57)	17.79	(13.33)	4.63	(45.53)
CEE/C. Asia	-32.87	(9.99)***	-5.36	(11.96)	-61.56	(41.38)
Asia	-26.85	(14.66)*	-48.02	(19.45)**	-218.72	(64.55)***
age × SSA					-2.17	(1.38)
age × LAC					-18.30	(2.15)***
age × MENA/S. Asia					0.19	(1.10)
age × CEE/C. Asia					1.48	(0.98)
age × Asia					5.49	(1.51)***
age^2 × SSA					0.01	(0.01)
age^2 × LAC					0.12	(0.02)***
age^2 × MENA/S. Asia					0.00	(0.01)
age^2 × CEE/C. Asia					-0.01	(0.01)*
age^2 × Asia					-0.04	(0.01)***
$\log(P_s)$			-43.06	(14.73)***	-273.97	(114.56)**
F × $\log(P_s)$					12.03	(2.42)***
Age × $\log(P_s)$					3.39	(1.17)***
Age^2 × $\log(P_s)$					-0.02	(0.01)***
$\log(P_f)$			94.54	(27.39)***	344.00	(93.37)***
F × $\log(P_f)$					-2.64	(3.42)
Age × $\log(P_f)$					-8.42	(2.20)***
Age^2 × $\log(P_f)$					0.06	(0.02)***
$\log(P_m)$			47.57	(19.43)**	170.34	(67.60)**
F × $\log(P_m)$					1.65	(2.58)
Age × $\log(P_m)$					-3.95	(1.59)**
Age^2 × $\log(P_m)$					0.03	(0.01)**
log(Y)			12.31	(4.92)**	159.23	(45.01)***
F × log(Y)					0.37	(0.70)
Age × log(Y)					-0.98	(0.42)**
Age^2 × log(Y)					0.01	(0.00)**
$\log(Y)^2$					-6.56	(2.24)***
$\log(P_s)$ × log(Y)					13.53	(9.53)
Adjusted R^2	0.65		0.70		0.81	

Note: Dependent variable is SSB intake in g/d (standard errors). $^{*}p \leq 0.10$; $^{**}p \leq 0.05$; $^{***}p \leq 0.01$. SSA = Sub-Saharan Africa. LAC = Latin America and the Caribbean. MENA/S. Asia = Middle East, North Africa, and South Asia. CEE/C. Asia = Central Europe, Eastern Europe, and Central Asia. Asia = Asian Pacific, East Asia, and Southeast Asia. The reference region consist of high-income Western countries and a few small island states. P_s = SSB price, P_f = 100% fruit juice price, P_m = milk price. All prices were deflated by a food price index. Y = real per-capita income.

For peer review only - http://bmjopen.bmj.com/site/about/guidelines.xhtml

For peer review only

Supplemental Table 4

Own-price elasticities of SSB intake by age, sex, and global income decile

Income decile	Age 20	25	30	35	40	45	50	55	60	65	70	75	80
	Women												
Lowest 10%	-0.58	-0.59	-0.62	-0.66	-0.73	-0.78	-0.76	-0.77	-0.80	-0.83	-0.84	-0.87	-0.96
	(0.22)	(0.23)	(0.25)	(0.28)	(0.33)	(0.37)	(0.39)	(0.41)	(0.44)	(0.47)	(0.49)	(0.51)	(0.55)
2nd	-0.53	-0.54	-0.56	-0.59	-0.64	-0.67	-0.65	-0.64	-0.66	-0.68	-0.68	-0.71	-0.79
	(0.20)	(0.21)	(0.22)	(0.24)	(0.28)	(0.30)	(0.31)	(0.33)	(0.35)	(0.37)	(0.38)	(0.4)	(0.43)
3rd	-0.49	-0.49	-0.49	-0.51	-0.55	-0.56	-0.53	-0.52	-0.52	-0.52	-0.52	-0.55	-0.61
	(0.18)	(0.18)	(0.19)	(0.2)	(0.22)	(0.24)	(0.24)	(0.25)	(0.26)	(0.28)	(0.28)	(0.29)	(0.32)
4th	-0.45	-0.44	-0.44	-0.45	-0.47	-0.47	-0.43	-0.41	-0.39	-0.39	-0.39	-0.40	-0.46
	(0.16)	(0.16)	(0.17)	(0.17)	(0.19)	(0.19)	(0.19)	(0.19)	(0.19)	(0.20)	(0.20)	(0.21)	(0.23)
5th	-0.42	-0.41	-0.41	-0.41	-0.42	-0.41	-0.37	-0.33	-0.31	-0.30	-0.30	-0.31	-0.36
	(0.15)	(0.15)	(0.15)	(0.16)	(0.17)	(0.17)	(0.16)	(0.16)	(0.16)	(0.16)	(0.16)	(0.17)	(0.19)
6th	-0.40	-0.39	-0.38	-0.37	-0.38	-0.36	-0.31	-0.28	-0.25	-0.23	-0.22	-0.24	-0.28
	(0.15)	(0.15)	(0.15)	(0.15)	(0.16)	(0.16)	(0.15)	(0.14)	(0.14)	(0.14)	(0.14)	(0.14)	(0.16)
7th	-0.38	-0.36	-0.35	-0.34	-0.34	-0.31	-0.26	-0.22	-0.19	-0.16	-0.15	-0.16	-0.20
	(0.15)	(0.14)	(0.14)	(0.14)	(0.15)	(0.15)	(0.14)	(0.13)	(0.13)	(0.13)	(0.13)	(0.14)	(0.16)
8th	-0.36	-0.33	-0.32	-0.30	-0.29	-0.25	-0.20	-0.15	-0.11	-0.08	-0.07	-0.07	-0.11
	(0.14)	(0.14)	(0.14)	(0.14)	(0.15)	(0.15)	(0.15)	(0.14)	(0.15)	(0.15)	(0.15)	(0.16)	(0.17)
9th	-0.33	-0.30	-0.28	-0.26	-0.23	-0.19	-0.13	-0.07	-0.03	0.01	0.03	0.03	0.00
	(0.14)	(0.14)	(0.14)	(0.15)	(0.16)	(0.17)	(0.17)	(0.17)	(0.18)	(0.19)	(0.19)	(0.20)	(0.22)
Highest 10%	-0.29	-0.26	-0.23	-0.20	-0.16	-0.10	-0.03	0.03	0.09	0.13	0.16	0.16	0.14
	(0.15)	(0.15)	(0.15)	(0.16)	(0.19)	(0.20)	(0.21)	(0.22)	(0.24)	(0.26)	(0.26)	(0.27)	(0.29)
	Men												
Lowest 10%	-0.58	-0.59	-0.62	-0.68	-0.78	-0.86	-0.87	-0.89	-0.92	-0.96	-0.98	-1.01	-1.10
	(0.20)	(0.21)	(0.23)	(0.26)	(0.31)	(0.35)	(0.37)	(0.39)	(0.42)	(0.45)	(0.46)	(0.47)	(0.51)
2nd	-0.54	-0.55	-0.57	-0.62	-0.70	-0.76	-0.76	-0.77	-0.79	-0.82	-0.83	-0.87	-0.94
	(0.18)	(0.19)	(0.20)	(0.22)	(0.26)	(0.29)	(0.3)	(0.31)	(0.33)	(0.35)	(0.36)	(0.38)	(0.40)
3rd	-0.50	-0.50	-0.51	-0.55	-0.62	-0.66	-0.65	-0.65	-0.66	-0.68	-0.69	-0.71	-0.78
	(0.16)	(0.17)	(0.17)	(0.19)	(0.21)	(0.23)	(0.23)	(0.24)	(0.25)	(0.26)	(0.27)	(0.28)	(0.30)
4th	-0.46	-0.46	-0.47	-0.49	-0.54	-0.58	-0.55	-0.55	-0.55	-0.56	-0.56	-0.58	-0.64
	(0.15)	(0.15)	(0.15)	(0.16)	(0.18)	(0.19)	(0.19)	(0.18)	(0.19)	(0.19)	(0.19)	(0.20)	(0.22)
5th	-0.44	-0.43	-0.44	-0.46	-0.50	-0.52	-0.49	-0.48	-0.47	-0.48	-0.48	-0.50	-0.55
	(0.14)	(0.14)	(0.14)	(0.15)	(0.16)	(0.17)	(0.16)	(0.16)	(0.16)	(0.16)	(0.16)	(0.16)	(0.18)
6th	-0.42	-0.41	-0.41	-0.43	-0.46	-0.47	-0.44	-0.42	-0.41	-0.41	-0.41	-0.43	-0.48
	(0.14)	(0.14)	(0.13)	(0.14)	(0.15)	(0.16)	(0.15)	(0.14)	(0.14)	(0.14)	(0.14)	(0.14)	(0.16)
7th	-0.40	-0.39	-0.39	-0.40	-0.42	-0.43	-0.39	-0.37	-0.36	-0.35	-0.34	-0.36	-0.41
	(0.13)	(0.13)	(0.13)	(0.14)	(0.15)	(0.15)	(0.14)	(0.14)	(0.13)	(0.13)	(0.13)	(0.14)	(0.15)
8th	-0.38	-0.36	-0.36	-0.36	-0.38	-0.37	-0.34	-0.31	-0.29	-0.27	-0.26	-0.28	-0.32
	(0.13)	(0.13)	(0.13)	(0.13)	(0.15)	(0.15)	(0.15)	(0.14)	(0.14)	(0.15)	(0.15)	(0.15)	(0.16)
9th	-0.35	-0.34	-0.32	-0.32	-0.33	-0.31	-0.27	-0.23	-0.21	-0.19	-0.18	-0.19	-0.22
	(0.13)	(0.13)	(0.13)	(0.14)	(0.15)	(0.16)	(0.16)	(0.17)	(0.17)	(0.18)	(0.18)	(0.19)	(0.20)
Highest 10%	-0.32	-0.30	-0.28	-0.27	-0.26	-0.23	-0.18	-0.14	-0.10	-0.07	-0.06	-0.07	-0.09
	(0.14)	(0.14)	(0.14)	(0.15)	(0.17)	(0.19)	(0.20)	(0.21)	(0.23)	(0.24)	(0.25)	(0.25)	(0.27)

Note: Values are derived at median intake levels by demographic subgroup (standard errors). Price elasticities are based on 1% price changes. For instance, given a 1% SSB price increase in the lowest income countries, intake by women, age 20 falls by 0.58%. Income deciles are based on the national income of the 164 countries included in the study. Each decile is composed of 16 countries except the 4 lowest deciles, which are each composed of 17 countries. The per capita income range (PPP-adjusted in thousand $US) for each decile: (1st) $0.6-$1.5, (2nd) $1.5-$2.7, (3rd) $2.7-$5.3, (4th) $5.5-$8.0, (5th) $8.3-$10.8, (6th) $11.1-$15.2, (7th) $15.3-$20.3, (8th) $20.6-$29.4, (9th) $30.4-$40.9, and (10th) $41.3-$127.2.

For peer review only - http://bmjopen.bmj.com/site/about/guidelines.xhtml

Supplemental Table 5

Cross-price elasticities of SSB intake with respect to changes in fruit juice and milk prices, by age and sex

	100% fruit juice		Milk	
	Women	Men	Women	Men
Age 20	1.05 (0.29)	0.96 (0.27)	0.56 (0.21)	0.49 (0.19)**
Age 30	1.02 (0.28)	0.92 (0.26)	0.56 (0.20)	0.49 (0.19)**
Age 40	1.07 (0.30)	1.00 (0.30)	0.62 (0.22)	0.55 (0.22)
Age 50	0.97 (0.28)	0.94 (0.29)	0.61 (0.21)	0.55 (0.21)
Age 60	0.90 (0.26)	0.88 (0.28)	0.60 (0.20)	0.53 (0.21)
Age 70	0.93 (0.26)	0.90 (0.27)	0.62 (0.20)	0.55 (0.21)
Age 80	1.17 (0.31)	1.12 (0.32)	0.75 (0.24)	0.65 (0.25)

Values represent the median elasticity (standard error). **$p \leq 0.05$; $p < 0.01$ for all others. Price elasticities are based on 1% price changes in these substitutes. For instance, given a 1% increase in fruit juice prices, SSB intake was estimated to increase by 1.05% in women, age 20 years.

For peer review only

Technical Appendix

Cross-country intake demand model and elasticities

To estimate SSB intake demand, we used a semi-logarithmic functional form that has been proven to be consistent with economic theory and rational consumer behavior.(1, 2) We applied a single-equation framework in this study. Prior studies have used a demand-system approach (multi-equation framework), primarily due to the adding-up property when using expenditure data (i.e., expenditures on all consumption categories "add up" to total expenditures), which results in the error terms being correlated across categories. Since this relationship does not exist with individual intakes, particularly when the correspondence between purchases and intakes is not one to one, the adopted approach is acceptable.

Let q_{ig} represent SSB intake by subgroup g (g: sex and age), p_i and p_j represent the price of SSBs and related good *j*, and Y and P represent real per capita income and overall food prices (all in country *C*). SSB intake demand by subgroup *g* in country *C* is specified as follows (*C* subscripts are omitted for convenience):

$$q_{ig} = \beta_0^* + \beta_1^* \ln(Y) + \beta_2^* \ln\left(\frac{p_i}{P}\right) + \beta_3^* \ln\left(\frac{p_j}{P}\right) + \beta_4^* \left[\ln(Y) \times \ln\left(\frac{p_i}{P}\right)\right] + \beta_5^* \ln(Y)^2 + u_{ig} \quad (1)$$

The β terms are coefficients to be estimated and u_{ig} is a random error term. The price terms (p_i and p_j) are deflated by P to discount differences due to overall food prices and to implicitly account for the cross-price effects of intake categories other than *i* and *j*. Note that the structure of the model allows for the relationship between own-price (p_i) and intake to vary by national income level.

We accounted for age, sex, and regional differences by allowing these factors to have a direct effect on intake, as well as an additional effect through income and prices.

$$\beta_i^* = \beta_{i0} + \beta_{i1} sex + \beta_{i2} age + \beta_{i3} age^2 + \sum_k \beta_{ik}\, region_k\,; i = 0,1,\ldots,5 \quad (2)$$

The variable *sex* is a binary (= 1 for women and 0 otherwise) and *age* is a continuous variable ranging from 20 to 80 in 5-year intervals. The age^2 term was added to allow for nonlinear age effects and the possibility of optimal responsiveness between

For peer review only - http://bmjopen.bmj.com/site/about/guidelines.xhtml

the youngest and oldest subgroup. We accounted for preferences across countries due to factors not related to income or prices by including 6 regional binary variables, including: Southeast Asia, East Asia, and High Income Asia Pacific (Asia) (13 countries); Central Europe, Eastern Europe, and Central Asia (CEE/C. Asia) (27); Latin America and the Caribbean (LAC) (30); Middle East, North Africa, and South Asia (MENA/S. Asia) (23); Sub-Saharan Africa (SSA) (45); and High Income/Rest of World (HIC) (26). HIC was comprised largely of Western, industrialized countries; while not geographically connected, these countries share other similarities. We included several small island countries in this grouping because they were not sufficiently numerous to merit their own regional grouping.

Given equation (1), the own-price elasticity is derived as follows:

$$\eta_{iig} = \frac{\%\Delta q_{ig}}{\%\Delta p_i} = \frac{1}{q_{ig}}[\beta_2^* + \beta_4^* \ln(Y)] \quad (3)$$

η_{iig} is the percentage change in intake (q_{ig}) (i: SSB) due to a 1% change in p_i, which should be negative since an increase in price usually results in a decrease in intake or quantity demanded. Note that if the β coefficients vary with sex, age, or region, equation (3) will vary accordingly. Price elasticities derived using the semi-logarithmic functional form depend on the intake level. As a result, elasticity values can be derived for each observation and at more aggregate levels.

The cross-price elasticity is similarly derived:

$$\eta_{ijg} = \frac{\%\Delta q_{ig}}{\%\Delta p_j} = \frac{\beta_3^*}{q_{ig}} \quad (4)$$

η_{ijg} is the percentage change in intake (q_{ig}) due to a 1% change in p_j (j: fruit juice, milk). Unlike the own-price elasticity, however, the cross-price elasticity does not depend on national income.

Relevant to this study would be the effect of a change in SSB prices on intake of fruit juice and milk (η_{jig}). According to economic theory, η_{jig} can be derived from η_{ijg} based on the following relationship (g subscripts are omitted for convenience):(3)

For peer review only - http://bmjopen.bmj.com/site/about/guidelines.xhtml

$$\eta_{ji} = \frac{s_i}{s_j}\eta_{ij} + s_i(\eta_{iY} - \eta_{jY}) \qquad (5)$$

s_i and s_j are the share of income spent on the *i*th and *j*th good, and η_{iY} and η_{jY} are the income elasticities for the *i*th and *j*th good, respectively. In this context, the second term can be assumed negligible because the share of SSB expenditures in total income (s_i) is likely to be small. Thus, the following approximation can be used to infer how a change in SSB prices would impact intake of fruit juice and milk:

$$\eta_{ji} \approx \frac{s_i}{s_j}\eta_{ij} \qquad (6)$$

References

1. LaFrance JT. Incomplete demand systems and semilogarithmic demand models. Australian J Agr Econ. 1990;34(2):118-31.
2. von Haefen RH. A complete characterization of the linear, log-linear, and semi-log incomplete demand system models. J Agr Resource Econ. 2002;27(2):281-319.
3. Tomek WG, Robinson KL. Agricultural Product Prices. Ithaca, New York: Cornell University Press; 1990.

For peer review only

BMJ Open

Global patterns in price elasticities of sugar-sweetened beverage intake and potential effectiveness of tax policy: results from 164 countries

Journal:	*BMJ Open*
Manuscript ID	bmjopen-2018-026390.R1
Article Type:	Research
Date Submitted by the Author:	09-Jan-2019
Complete List of Authors:	Muhammad, Andrew; University of Tennessee Institute of Agriculture<br>Meade, Birgit; USDA Economic Research Service<br>Marquardt, David; USDA Economic Research Service<br>Mozaffarian, Dariush; Friedman School of Nutrition Science and Policy, Tufts University,
**Primary Subject Heading**:	Global health
Secondary Subject Heading:	Health economics, Health policy
Keywords:	elasticity, intake, global, prices, sugar-sweetened beverages, taxes

SCHOLARONE™
Manuscripts

Global patterns in price elasticities of sugar-sweetened beverage intake and potential effectiveness of tax policy: results from 164 countries

Andrew Muhammad,[1] Birgit Meade,[2] David R Marquardt,[2] and Dariush Mozaffarian[3]

(Corresponding author) Andrew Muhammad, University of Tennessee Institute of Agriculture, Department of Agricultural and Resource Economics, 2621 Morgan Circle, 302 Morgan Hall Knoxville, Tennessee 37996-4518, USA

Email: amuhammad@utk.edu

[1]University of Tennessee Institute of Agriculture, Department of Agricultural and Resource Economics, Knoxville, Tennessee, USA

[2]United States Department of Agriculture, Economic Research Service, Market and Trade Economics Division, Washington, D.C., USA

[3]Tufts University, Friedman School of Nutrition Science and Policy, Boston, Massachusetts, USA

Keywords: demand, diet, elasticity, intake, global, prices, sugar-sweetened beverages, taxes

Disclaimer: The views expressed are those of the authors, and may not be attributed to the Economic Research Service or the U.S. Department of Agriculture.

Copyright: The Corresponding Author has the right to grant on behalf of all authors and does grant on behalf of all authors, a worldwide licence (or non-exclusive for government employees) to the Publishers and its licensees in perpetuity, in all forms, formats and media (whether known now or created in the future), to i) publish, reproduce, distribute, display and store the Contribution, ii) translate the Contribution into other languages, create adaptations, reprints, include within collections and create summaries, extracts and/or, abstracts of the Contribution, iii) create any other derivative work(s) based on the Contribution, iv) to exploit all subsidiary rights in the Contribution, v) the inclusion of electronic links from the Contribution to third party material where-ever it may be located; and, vi) licence any third party to do any or all of the above.

For peer review only - http://bmjopen.bmj.com/site/about/guidelines.xhtml

Competing interests: We have read and understood BMJ policy on declaration of interests and declare that we have no competing interests.

Contributors: AM conceptualized the study and was responsible for the study design, model estimations, and contributed to the interpretation of results. AM, DM, and BM contributed to the interpretation of results and discussion. DM wrote and edited sections describing the intake data. BM was primarily responsible for the literature review and facilitated the data agreement with the International Comparison Program, World Bank. DM provided the intake data and obtained the funding. DRM and AM were responsible for the visualizations and corresponding text. AM was the primary author, but all authors contributed to writing the manuscript. AM is the manuscript's guarantor.

Data sharing: Real per capita income data were obtained from the World Development Indicators Data Bank. https://databank.worldbank.org/data/reports.aspx?source=world-development-indicators. SSB Intake data are available upon request from the Global Dietary Database. http://www.globaldietarydatabase.org/. Price indexes for select food categories are available upon request from the World Bank, International Comparison Program. icp@worldbank.org.

Ethical approval: Not required.

Clinical trial registration: Not required.

Transparency declaration: The lead author* affirms that this manuscript is an honest, accurate, and transparent account of the study being reported; that no important aspects of the study have been omitted; and that any discrepancies from the study as planned (and, if relevant, registered) have been explained. *The manuscript's guarantor.

Funding: The Bill & Melinda Gates Foundation (project: Global Dietary Habits among Women, Price and Income Elasticities, and Validity of Food Balance Sheets).

For peer review only - http://bmjopen.bmj.com/site/about/guidelines.xhtml

Role of the funding source: The funders had no role in the study design, study conduct, data analysis, data interpretation, or writing of the report. All authors had full access to all the data and take responsibility for the integrity of the data and the accuracy of the analysis. The corresponding author had final responsibility to submit the report for publication.

For peer review only

For peer review only - http://bmjopen.bmj.com/site/about/guidelines.xhtml

Abstract

Objective – To quantify global relationships between sugar-sweetened beverage (SSB) intake and prices and examine the potential effectiveness of tax policy by age, sex, and country.

Design – SSB intake data by country, age, and sex from the Global Dietary Database were combined with national GDP and food price data from the World Bank International Comparison program. Intake responsiveness to income and prices was estimated accounting for national income, age, and sex differences.

Setting – 164 countries.

Population – Full adult population in each country.

Main outcome measures – A consumer demand modeling framework was used to estimate the relationship between SSB intake and prices (own and substitute prices) and derived price elasticities of SSB intake (measures of the percentage change in intake from a 1% change in price) by age, sex, and country. We simulated how a 20% tax would impact SSB intake globally. Tax policy outcomes (projected intake reductions) were examined across countries by income decile for representative age and sex subgroups.

Results – Own-price responsiveness of SSB intake was highest in lowest income countries, ranging from -0.70 (p<0.100) for women, age 50, to -1.91 (p<0.001) for men, age 80. In the highest income countries, responsiveness was as high as -0.49 (p<0.001) (men, age 20), but was mostly insignificant for older adults. Overall, elasticities were strongest (more negative) at the youngest and oldest age groups, and were mostly insignificant for middle-aged adults, particularly in middle-income and high-income countries. Sex differences were mostly negligible. Potential intake reductions from a 20% national tax in lowest income countries ranged from 14.5% (95% CI: 29.5%, -0.4%) in women, 35 ≤ age < 60, to 24.9% (44.4%, 5.3%) in men, age ≥ 60. Potential intake reductions decreased with country income overall, and were mostly insignificant for middle-aged adults.

Conclusions – These findings estimate the global price-responsiveness of SSB intake by country, age, and sex, informing ongoing policy discussions on potential effects of SSB taxes.

For peer review only - http://bmjopen.bmj.com/site/about/guidelines.xhtml

Strengths and limitations of this study

- First study to examine SSB intake and taxation in a global context, providing a better understanding of tax-policy effectiveness across the complete spectrum of countries.
- Results quantify the potential variability in influence of price on SSB intake across countries including by age and sex, suggesting that outcomes of SSB taxes may be significantly influenced by age and the income status of countries.
- Being a modeling study, the projected outcomes can only inform how taxes could affect behavior.
- Cross-country analysis of this scope rely on specific data collection initiatives that often do not occur on an annual basis and/or do not provide specific variables; proxy variables are needed when data are not available.

For peer review only

Introduction

Taxation of sugar-sweetened beverages (SSBs) has received growing attention, given their links to excessive weight gain and increased risk of obesity, type-2 diabetes, and other non-communicable diseases (NCDs).(1-5) Arguably, taxation is not punitive but market normalizing, as the true costs of SSBs due to public health-care expenditures and other societal costs from excessive intake are not reflected in current market prices. Thus, by increasing SSB prices relative to other foods, taxes can play a role in decreasing consumption, lowering societal costs, and improving societal wellbeing.(6, 7) Based on these considerations, a rapidly growing number of countries have implemented or announced national SSB taxes, (8, 9) including Norway in 1981 and Samoa in 1984; Australia, French Polynesia, Fiji, and Nauru between 2000 and 2007; and Finland, Hungary, France, Chile, Mexico, Barbados, St. Helena, and Dominica since 2011. In 2018, Estonia, the Philippines, the UK, South Africa, the Republic of Ireland, Peru, and Norway implemented SSB taxes. Colombia and Saudi Arabia have included such taxes in recent proposals, while Bermuda, India and Indonesia are considering similar measures. In the U.S., more than 30 jurisdictions have implemented or attempted to pass SSB taxes since 2016, including San Francisco and Seattle in 2018.(10, 11) Despite their growing acceptance globally, the potential impact of SSB taxation on intake remains uncertain, particularly how it might vary across countries, and by age and sex within countries.

Most studies of SSB taxation have been limited to a small group of countries or focused on a specific country or jurisdiction where taxes have been implemented.(12-17) No study to date has examined SSB consumption and taxation in a global context. In addition, few studies have considered how SSB intake could vary depending on the price of substitute products.(18) Because expert organizations are advocating and governments are considering SSB taxation

For peer review only

For peer review only - http://bmjopen.bmj.com/site/about/guidelines.xhtml

across the globe,(19) examining demand in a global context can provide a better understanding of potential tax-policy effectiveness across the complete spectrum of countries, from most to least developed.

To investigate this issue, we examined SSB intake across 164 countries and estimated how intake differences within and across countries are influenced by the price of SSBs and substitute caloric beverages (fruit juice and milk), as well as other factors such as national income, age, and sex. Based on WHO recommendations,(19) we further simulated how SSB intake would respond to a 20% tax (price increase). Tax-policy outcomes were examined across countries by income decile for representative age and sex subgroups.

Methods

Using globally representative intake and pricing data, we implemented a consumer demand modeling framework to examine determinants of SSB intake within and across countries. The modeling framework accounted for age and sex differences and economic determinants such as own price, price of substitutes (fruit juice and milk), and real per capita income at the national level. We also considered the potential for unmeasured region-specific differences, such as taste or other preferences, by including regional binary variables. Model estimates were used to derive SSB own-price elasticities for detailed strata (age, sex, and countries by income decile), and to assess the potential impact of taxes on intake. Accounting for these factors, we report price elasticities of SSB intake (measures of the percentage change in intake from a 1% change in price), which have been a primary means of estimating potential tax-policy effectiveness.(20) We also evaluated the variability in tax-policy effectiveness and examined outcomes for select age and sex subgroups and countries by income decile.

For peer review only - http://bmjopen.bmj.com/site/about/guidelines.xhtml

Data and sources

Data on SSB intake were derived from the 2010 Global Dietary Database (GDD), a database of global food and nutrient intakes by age (20-80 in 5-year intervals) and sex for 187 countries. The SSB category in the GDD includes intake of all sugar-sweetened beverages, including any beverage with added sugar and ≥ 50 kcal per 8 oz., such as carbonated beverages, sodas, energy drinks, fruit drinks, etc., excluding 100% juices. GDD data collection, statistical methods, data validation, and findings have been described in detail (also see http://www.globaldietarydatabase.org/).(21-25) In brief, GDD data were derived based on national and subnational dietary surveys, informed by additional information from United Nations Food and Agricultural Organization (FAO) food balance sheets data, individual-level surveys from cohort studies, household expenditure surveys when dietary surveys were not available, as well as other data sources such as the World Health Organization (WHO) Global Infobase and the WHO STEPS database.(25)

For prices, we used global price indices from the 2011 International Comparison Program (ICP) of the World Bank (see supplemental table 1).(26, 27) The ICP is a worldwide statistical initiative that produces price and expenditure data on consumer goods, services, and capital goods. The price indices used in this study are standardized to a common currency, the U.S. dollar in this case. Our choice of price variables was limited by inadequate data on a global scale. For instance, the ICP categories included milk but not SSBs and fruit juice. For SSBs, we used the ICP price index for sugar, which is justified, in part, due to sugar being a defining input. Similarly, we used the ICP fresh or chilled fruit price index as a proxy for fruit juice prices. Since sugar or fresh fruit may not be a major share of the final product price, particularly in rich

For peer review only - http://bmjopen.bmj.com/site/about/guidelines.xhtml

countries, there are limitations to these proxies. In view of this, we adjusted the sugar and fresh fruit price indexes according to national income level using information on the value-added share of farm products in U.S. food and beverage production (https://www.ers.usda.gov/data-products/food-dollar-series.aspx). This procedure resulted in relatively higher prices at higher income levels. Details are in the supplement (see supplementary information, technical appendix).

We divided each price series by an aggregate price level index for *food and nonalcoholic beverages* to adjust for differences in overall food prices across countries. This discounts any price differences across countries due to differences in overall food costs and implicitly accounts for the cross-price effects of food products not in the model.

The current analysis included 164 countries (4,264 stratum observations) having both GDD intake and ICP price data.

For national income, we used 2010 gross domestic product (GDP) data expressed in U.S. dollars per capita from the World Bank Development Indicators Database.(28) To account for differences in currency and purchasing power across economies, we used purchasing power parity (PPP) adjusted GDP. Since PPP-adjusted GDP accounts for inflationary factors across countries, we refer to our income measure as *real* per capita GDP. Income deciles were based on real per capita GDP for the 164 countries in the study.

Model and analysis

To estimate SSB intake demand, we applied a single-equation framework and used a semi-logarithmic functional form (see supplementary information, technical appendix).(29, 30) Many studies have used a double-log quadratic form.(31) However, a problem with the double-

For peer review only - http://bmjopen.bmj.com/site/about/guidelines.xhtml

log form is that significant intake differences across subgroups can be lost in log conversions. A semi-log relationship allowed for a better assessment of subgroup effects on intake responsiveness. It has also been shown that semi-log models of demand are consistent with economic theory and contain the necessary information for obtaining, for instance, reliable measures of consumer welfare and the underlying preference structure of consumers.(29) Prior studies have also used a demand-system approach (multi-equation framework), primarily due to the need to account for the adding-up property when using expenditure data (i.e., expenditures on all consumption categories "add up" to total expenditures), which results in the error terms being correlated across categories. Since we are not estimating demand using an expenditure or allocation framework, the adopted approach is acceptable.

We accounted for age, sex, and regional differences by allowing these factors to have a direct effect on intake, as well as an additional effect through income and prices, including a quadratic age term to allow for nonlinear effects and the possibility of optimal responsiveness being between the youngest and oldest subgroups.

For peer review only

We accounted for varying preferences across countries due to factors not related to income or prices by including regional binary variables in the model: Southeast Asia, East Asia, and High Income Asia Pacific (Asia) (13 countries); Central Europe, Eastern Europe, and Central Asia (CEE/C. Asia) (27); Latin America and the Caribbean (LAC) (30); Middle East, North Africa, and South Asia (MENA/S. Asia) (23); Sub-Saharan Africa (SSA) (45); and High Income/Rest of World (HIC) (26). HIC was comprised largely of Western, industrialized countries; while not geographically connected, these countries share other similarities. We included several small island countries in this grouping because they were not sufficiently numerous to merit their own regional grouping (see supplemental table 2).

We utilized F-tests to compare a model including all explanatory variables and interaction terms to a series of restricted models and arrived at the final parsimonious model. Least-squares regression treats data independently and does not account for within-country correlations resulting in biased and comparatively small standard errors. Correcting for this, all models were estimated assuming country clusters, that is, independent errors across countries but correlated errors within countries, as well as heteroskedastic-consistent errors.(32) The elasticities reported in the following section were derived using the estimated coefficients from model 3 (final model) (see supplemental table 3).

Given WHO recommendations, we simulated how SSB intake would respond to a 20% tax (price increase).(19) Results were evaluated across countries by income decile for the following demographic subgroups: men and women, age < 35, 35-59, ≥ 60 years. We used probabilistic sensitivity analyses (Monte Carlo simulations) to derive 95% confidence intervals of intake responsiveness to the tax. Confidence intervals were based on the covariance matrix of

For peer review only - http://bmjopen.bmj.com/site/about/guidelines.xhtml

the estimated coefficients, which accounted for the variability in the own-price relationship and the additional variability due to age, sex, and national income level.

Patient and public involvement

Patients and public were not involved.

Results

Global SSB intake

SSB intake levels varied significantly across countries (see supplemental figure 1) and by world region and age (figure 1). LAC had the highest median intake at 311 g/d (men) and 288 g/d (women) – almost four times the intake in SSA, and six times the lowest intake region (Asia). Across age/sex strata globally, the group with the highest median intake was young men, age 20 (209 g/d), followed closely by young women, age 20 (188 g/d). Compared to 20-year olds, median global intake in men and women, age 80, was about 75% lower. Across age and sex strata worldwide, the highest intake level was observed for men, age 20, in Trinidad and Tobago (1,239 g/d), and the lowest intake for women, age 80, in China (6 g/d). A more detailed discussion of global SSB intake by age, sex, and world region is available.(33)

SSB own-price elasticities

Given the variables in the final model, it was more appropriate to derive elasticities across country groups based on income level. We derived and compared SSB own-price elasticities across all strata jointly by age, sex, and global income decile (figure 2 and table 1; also see supplemental table 4). Note that reported values are derived at median intake levels by age and sex subgroup. Thus, observed differences across age, sex, and income decile are solely a

For peer review only - http://bmjopen.bmj.com/site/about/guidelines.xhtml
For peer review only

function of own-price interactions with sex, age, and income. At any given age, SSB intake became less responsive to price changes with rising income. For instance, in women, age 20, the own-price elasticities ranged from -0.90 ($p<0.001$) for the lowest income decile to -0.47 ($p<0.001$) for the highest income decile. The decline in responsiveness became more pronounced with age. For instance, in men, age 80, the own-price elasticities ranged from -1.91 ($p<0.001$) for the lowest income decile to -0.43 ($p>0.100$) for the highest income decile. The influence of age on SSB own-price elasticities varied depending on income status. At lower income levels, elasticities were strongest (became more negative) at older ages; but at middle and higher income levels, there was less influence of age on elasticities. The least responsive group were middle-aged adults, particularly in upper-middle and higher income deciles.

Potential impact of SSB taxes on intake

Potential reductions in median intake from a 20% tax (price increase) were largest for the lowest income decile, ranging from 14.5% (95% CI: -0.4, 29.5) to 24.1% (5.3, 44.4), depending on age and sex (table 2). Across income deciles, reductions varied less in younger adults (age < 35) – for example, ranging from 16.8% (8.6, 25.0) in young men in the lowest income decile to 7.9% (2.2, 13.6) in the highest income decile – than in older adults (e.g., men, age ≥ 60). This is consistent with the much higher baseline SSB intakes among younger adults globally (figure 1), suggesting that such intake will be significantly influenced by taxes regardless of income status. Older men and women (age ≥ 60) in the lowest income decile were estimated to be most influenced by SSB taxes, suggesting a high price-responsiveness to such a luxury in poor nations globally. Insignificant outcomes were mostly observed for middle-aged and older adults in middle and higher income deciles.

For peer review only - http://bmjopen.bmj.com/site/about/guidelines.xhtml

Discussion

In this global analysis of SSB intakes and prices, we identified significant price responsiveness in nearly every age, sex, and country income subgroup worldwide. We also identified significant heterogeneity in these potential responses. Price responsiveness was higher in lower income than in wealthier countries, consistent with expectations and the much higher relative share of income spent on food and other necessities in low-income countries. Interestingly, the response by age varied by national income. In lower income countries, own-price responsiveness increased with age, but less so in middle and higher income countries.

Finally, our estimates of effects of a 20% tax suggested significant SSB intake reductions across income levels, particularly for young adults. Outcomes for middle-aged adults, and older adults at higher income levels, were not significant.

Strengths and limitations

This study has several strengths, the first being the extensive country coverage. We provide a global snapshot of SSB intake behavior allowing for comparisons within and across most countries. Since past studies have been limited to a single country or a select group of countries, the results of this study inform policy and decision-making beyond the current state of knowledge. Problems associated with poor diets and NCDs occur in both developing and developed countries.(34) A comparative analysis across the complete spectrum of countries can assist international organizations in developing heterogeneous strategies for specific subgroups and countries. Our use of individual intakes by age, sex, and country provides for more accurate

For peer review only - http://bmjopen.bmj.com/site/about/guidelines.xhtml

representation of dietary behavior. Previous findings based on expenditure data may be limited by differences in expenditures and actual consumption.

Potential limitations should also be considered. First, being a modeling study, the projected outcomes can only inform how taxes could affect behavior. While an intervention study would be more fitting, interventions across 164 countries would not be feasible. Secondly, our analysis was limited by the use of price and income data at the national level. Ideally, our explanatory variables would also be at the subgroup level, reflecting that incomes typically vary with age and sex, and different subgroups could face a different set of prices within a country. For instance, in countries where urban populations are relatively young, young adults could face different prices depending on market conditions in urban and rural areas. This limitation is due to the number of countries in our study. Such detailed data is not available for many countries.

While it would be ideal to have a time series of global SSB intake data, unfortunately these data do not exist. However, there is value in examining data at a point-in-time and intake in one demographic group compared to other groups, as well as comparing intake patterns across countries. Our purpose is to inform how demographic subgroups across countries might respond to price signals in form of taxes. There is value in understanding the relative responsiveness which can be gleaned from a cross-country snapshot.

The use of the global sugar prices as a proxy for SSB prices raises questions about the primary relationship of interest (SSB own-price elasticity). For higher income countries where farm production costs are a small share of the final product price, the proxy is less suitable and could result is lower "own-price" responsiveness. Accordingly, we adjusted the price index to account for higher SSB prices relative to sugar prices at higher income levels. The adjustment resulted in a 10- to 15-fold increase in the index value for higher income countries similar to the

For peer review only - http://bmjopen.bmj.com/site/about/guidelines.xhtml

For peer review only

U.S. For low-income countries, adjusted and unadjusted prices were not that dissimilar (see supplemental figure 2). Using adjusted prices, we found significantly higher own-price responsiveness compared to estimates using unadjusted prices.

Comparison with other studies

Since previous research has mostly focused on higher income countries, primarily the U.S., it is difficult to compare all of our results with earlier findings. Several U.S. based studies have considered how SSB consumption would respond to a tax. Given a 10% tax, the projected decrease in SSB sales ranged from 6.7% to 18.2%.(15) These results are greater than our findings for middle-aged and older adults in the highest income decile, but are closer to our findings for young adults (7.3%, women, age < 35, and 7.9%, men, age < 35), albeit we are considering a 20% tax.

Our tax outcomes are due to comparably smaller own-price elasticities. Whereas our own-price elasticity estimates for the highest income countries range from -0.5 to -0.0, meta-analyses of U.S. studies give estimates of -0.8 (-3.2 to -0.13) and -1.1 (-1.3 to -0.9).(16, 35) In a study of Mexico using data before and after implementation of a national soda tax (10%) in 2014, SSB purchases decreased by an average of 6% during the first year of implementation,(12) which is actually comparable to our findings for young adults in middle-income countries. Other studies of Latin American countries using household survey data reported estimates more comparable to our results for lower income countries.(36-38)

The fact that our estimates are relative smaller does not necessarily make them less accurate. Note that past studies have mostly used expenditure data. It has been documented that significant changes in expenditures do not always result in changes in the quantity or quality of

For peer review only - http://bmjopen.bmj.com/site/about/guidelines.xhtml

food consumption.(39) In fact, studies have found the association between food expenditures and intake to be particularly weak and insufficient for diet and nutrition research.(40) For instance, a recent study of the SSB tax in Berkeley, California, U.S. found significant reductions in consumer spending on SSBs, increased spending on substitute beverages, but insignificant reductions in reported SSB intake.(41) Another issue is that SSBs are less perishable that other foods. When goods have an extended shelf life, individuals can take advantage of price discounts, increasing expenditures when prices are low, stock piling for future consumption. Ignoring this fact can result in overestimates of own-price elasticities.(42)

Conclusion

This is the first study to examine SSB consumption and taxation in a global context. Our findings provide a better understanding of the potential effectiveness of taxes across the full spectrum of countries. Overall, we found that the influence of SSB prices on intake significantly depends on the income status of countries. Our results suggest that intake reductions (in percent) could be small or negligible for certain demographics in higher income countries. Although small in percentage terms, actual intake reductions could still be sizeable enough for high-consuming subgroups for taxes to be worth pursuing. For higher income countries, a larger tax or a tax combined with other approaches might be needed to significantly change behavior. For instance, taxes could be combined with media and education campaigns, food labeling, and other interventions.(43) For all adults in lower income countries and young adults globally, our findings indicate that taxes would be particularly effective, which is to be expected since food expenditures account for a greater share of income for these groups making them more sensitive to prices.

For peer review only - http://bmjopen.bmj.com/site/about/guidelines.xhtml

For peer review only

For peer review only

For peer review only - http://bmjopen.bmj.com/site/about/guidelines.xhtml

References

1. de Koning L, Malik VS, Kellogg MD, Rimm EB, Willett WC, Hu FB. Sweetened beverage consumption, incident coronary heart disease, and biomarkers of risk in men. Circulation. 2012;125(14):1735-41.

2. Fung TT, Malik V, Rexrode KM, Manson JE, Willett WC, Hu FB. Sweetened beverage consumption and risk of coronary heart disease in women. The American Journal of Clinical Nutrition. 2009;89(4):1037-42.

3. Ludwig DS, Peterson KE, Gortmaker SL. Relation between consumption of sugar-sweetened drinks and childhood obesity: a prospective, observational analysis. Lancet. 2001;357(9255):505-8.

4. Malik VS, Popkin BM, Bray GA, Després J-P, Willett WC, Hu FB. Sugar-sweetened beverages and risk of metabolic syndrome and type 2 diabetes. Diabetes Care. 2010;33(11):2477-83.

5. Mozaffarian D, Hao T, Rimm EB, Willett WC, Hu FB. Changes in diet and lifestyle and long-term weight gain in women and men. N Engl J Med. 2011;364(25):2392-404.

6. Eyles H, Ni Mhurchu C, Nghiem N, Blakely T. Food pricing strategies, population diets, and non-communicable disease: a systematic review of simulation studies. PLoS Med. 2012;9(12):e1001353.

7. World Health Organization. Using Price Policies to Promote Healthier Diets. Copenhagen, Denmark: WHO Regional Office for Europe; 2015.

8. Gustin G. Latest Soda Tax Puts Pressure on Waistlines and the World. National Geographic. 2016 March 17, 2016.

9. Grogger J. Soda Taxes and the Prices of Sodas and Other Drinks: Evidence from Mexico. National Bureau of Economic Research; 2015. Contract No.: NBER Working Paper No. 21197.

10. Paarlberg R, Mozaffarian D, Micha R. Viewpoint: Can U.S. local soda taxes continue to spread? Food Pol. 2017;71:1-7.

11. Backholer K, Vandevijvere S, Blake M, Tseng M. Sugar-sweetened beverage taxes in 2018: a year of reflections and consolidation. Public Health Nutr. 2018;21(18):3291-5.

12. Colchero MA, Popkin BM, Rivera JA, Ng SW. Beverage Purchases from Stores in Mexico Under the Excise Tax on Sugar Sweetened Beverages: Observational Study. BMJ. 2016;352:h6704.

13. Brownell KD, Farley T, Willett WC, Popkin BM, Chaloupka FJ, Thompson JW, et al. The public health and economic benefits of taxing sugar-sweetened beverages. N Engl J Med. 2009;361(16):1599-605.

For peer review only - http://bmjopen.bmj.com/site/about/guidelines.xhtml

14. Nakhimovsky SS, Feigl AB, Avila C, O'Sullivan G, Macgregor-Skinner E, Spranca M. Taxes on sugar-sweetened beverages to reduce overweight and obesity in middle-income countries: a systematic review. PLoS One. 2016;11(9):e0163358.

15. Afshin A, Peñalvo JL, Del Gobbo L, Silva J, Michaelson M, O'Flaherty M, et al. The prospective impact of food pricing on improving dietary consumption: A systematic review and meta-analysis. PLoS One. 2017;12(3):e0172277.

16. Powell LM, Chriqui JF, Khan T, Wada R, Chaloupka FJ. Assessing the potential effectiveness of food and beverage taxes and subsidies for improving public health: a systematic review of prices, demand and body weight outcomes. Obes Rev. 2013;14(2):110-28.

17. Briggs ADM, Mytton OT, Kehlbacher A, Tiffin R, Rayner M, Scarborough P. Overall and income specific effect on prevalence of overweight and obesity of 20% sugar sweetened drink tax in UK: econometric and comparative risk assessment modelling study. BMJ. 2013;347:f6189.

18. Grogger J. Soda taxes and the prices of sodas and other drinks: evidence from Mexico. Amer J Agr Econ. 2017;99(2):481-98.

19. World Health Organization. Fiscal Policies for Diet and the Prevention of Noncommunicable Diseases. Technical Meeting Report. Geneva, Switzerland: World Health Organization; 2016.

20. Mytton OT, Clarke D, Rayner M. Taxing unhealthy food and drinks to improve health. BMJ. 2012;344(7857):e2931.

21. Del Gobbo LC, Khatibzadeh S, Imamura F, Micha R, Shi P, Smith M, et al. Assessing global dietary habits: a comparison of national estimates from the FAO and the Global Dietary Database. Am J Clin Nutr. 2015;101(5):1038-46.

22. Imamura F, Micha R, Khatibzadeh S, Fahimi S, Shi P, Powles J, et al. Dietary quality among men and women in 187 countries in 1990 and 2010: a systematic assessment. Lancet Global Health. 2015;3(3):e132-e42.

23. Micha R, Kalantarian S, Wirojratana P, Byers T, Danaei G, Elmadfa I, et al. Estimating the global and regional burden of suboptimal nutrition on chronic disease: methods and inputs to the analysis. Eur J Clin Nutr. 2012;66(1):119-29.

24. Micha R, Khatibzadeh S, Shi P, Fahimi S, Lim S, Andrews KG, et al. Global, regional, and national consumption levels of dietary fats and oils in 1990 and 2010: a systematic analysis including 266 country-specific nutrition surveys. BMJ. 2014;348:g2272.

25. Khatibzadeh S, Kashaf MS, Micha R, Fahimi S, Shi P, Elmadfa I, et al. A global database of food and nutrient consumption. Bull World Health Organ. 2016;94(12):931-4E.

26. World Bank. Purchasing Power Parities and Real Expenditures of World Economies Washington, D.C.: World Bank Publications; 2014.

For peer review only

For peer review only - http://bmjopen.bmj.com/site/about/guidelines.xhtml

27. World Bank. Purchasing Power Parities and the Real Size of World Economies Washington, D.C.: World Bank Publications; 2015.

28. World Bank. World Development Indicators 2015 [Available from: http://data.worldbank.org/data-catalog/world-development-indicators.

29. LaFrance JT. Incomplete demand systems and semilogarithmic demand models. Australian J Agr Econ. 1990;34(2):118-31.

30. von Haefen RH. A complete characterization of the linear, log-linear, and semi-log incomplete demand system models. J Agr Resource Econ. 2002;27(2):281-319.

31. Sahn DE. The Effect of Price and Income Changes on Food-Energy Intake in Sri Lanka. Econ Devel Cult Change. 1988;36(2):315-40.

32. Cameron AC, Trivedi PK. Microeconometrics: Methods and Applications. Cambridge: Cambridge University Press; 2005.

33. Singh GM, Micha R, Khatibzadeh S, Shi P, Lim S, Andrews KG, et al. Global, regional, and national consumption of sugar-sweetened beverages, fruit juices, and milk: a systematic assessment of beverage intake in 187 countries. PLoS One. 2015;10(8):e0124845.

34. United Nations. Note by the Secretary-General transmitting the report of the Director-General of the World Health Organization on the prevention and control of non-communicable diseases. New York: United Nations; 2013. Report No.: A/68/650 Contract No.: A/68/650.

35. Andreyeva T, Long MW, Brownell KD. The impact of food prices on consumption: a systematic review of research on the price elasticity of demand for food. Am J Public Health. 2010;100(2):216-22.

36. Colchero MA, Salgado JC, Unar-Munguía M, Hernández-Ávila M, Rivera-Dommarco JA. Price elasticity of the demand for sugar sweetened beverages and soft drinks in Mexico. Econ Hum Biol. 2015;19:129-37.

37. Guerrero-López CM, Unar-Munguía M, Colchero MA. Price elasticity of the demand for soft drinks, other sugar-sweetened beverages and energy dense food in Chile. BMC Public Health. 2017;17(1):180.

38. Paraje G. The Effect of Price and Socio-Economic Level on the Consumption of Sugar-Sweetened Beverages (SSB): The Case of Ecuador. PLoS One. 2016;11(3):e0152260.

39. Mark Aguiar, Erik Hurst. Consumption versus Expenditure. J Polit Economy. 2005;113(5):919-48.

40. Monsivais P, Perrigue MM, Adams SL, Drewnowski A. Measuring diet cost at the individual level: a comparison of three methods. Eur J Clin Nutr. 2013;67:1220.

For peer review only - http://bmjopen.bmj.com/site/about/guidelines.xhtml

41. Silver LD, Ng SW, Ryan-Ibarra S, Taillie LS, Induni M, Miles DR, et al. Changes in prices, sales, consumer spending, and beverage consumption one year after a tax on sugar-sweetened beverages in Berkeley, California, US: A before-and-after study. PLoS Med. 2017;14(4):e1002283.

42. Hartmann WR. Intertemporal effects of consumption and their implications for demand elasticity estimates. Quantitative Marketing and Economics. 2006;4(4):325-49.

43. Afshin A, Micha R, Khatibzadeh S, Schmidt LA, Mozaffarian D. Dietary Policies to Reduce Noncommunicable Diseases. In: Brown GW, Yamey G, Wamala S, editors. The Handbook of Global Health Policy. West Sussex, UK: Wiley-Blackwell; 2014. p. 175-93.

For peer review only

Figure 1 Comparison of mean SSB intakes among adults in age, sex, and country-specific strata across world regions and globally by select age groups. *n* represents the number of age, sex, and country-specific subgroups in each stratum. Boxes represent the median intake Value and interquartile range; error bars represent the minimum and maximum values. Source: Global Dietary Database, 2010

Figure 2 Global SSB own-price elasticities by age, sex, and global income decile. Values are derived at median intake levels by demographic subgroup. Own-price elasticities are based on 1% price changes. Income deciles are based on the national income of the 164 countries included in the study. Each decile is comprised of 16 countries (except the 4 lowest deciles, which are each comprised of 17 countries). The per capita income range (PPP-adjusted in thousand $US) for each decile: (1st) $0.6-$1.5, (2nd) $1.5-$2.7, (3rd) $2.7-$5.3, (4th) $5.5-$8.0, (5th) $8.3-$10.8, (6th) $11.1-$15.2, (7th) $15.3-$20.3, (8th) $20.6-$29.4, (9th) $30.4-$40.9, and (10th) $41.3-$127.2.

For peer review only

For peer review only - http://bmjopen.bmj.com/site/about/guidelines.xhtml

Table 1 Own-price elasticities of SSB intake by age, sex, and global income decile†

Income decile‡	Age 20	Age 30	Age 40	Age 50	Age 60	Age 70	Age 80	Population-weighted average
	Women							
Lowest 10%	-0.90 (0.21)***	-0.80 (0.25)***	-0.78 (0.35)**	-0.70 (0.42)*	-0.78 (0.49)	-1.11 (0.53)**	-1.84 (0.60)***	-0.82 (0.30)***
2nd	-0.83 (0.18)***	-0.71 (0.21)***	-0.65 (0.29)**	-0.54 (0.34)	-0.58 (0.38)	-0.88 (0.41)**	-1.59 (0.46)***	-0.71 (0.25)***
3rd	-0.76 (0.16)***	-0.62 (0.18)***	-0.51 (0.23)**	-0.36 (0.27)	-0.37 (0.29)	-0.65 (0.30)**	-1.33 (0.34)***	-0.59 (0.21)***
4th	-0.70 (0.14)***	-0.54 (0.16)***	-0.40 (0.20)**	-0.22 (0.22)	-0.19 (0.22)	-0.45 (0.21)**	-1.10 (0.24)***	-0.49 (0.17)***
5th	-0.67 (0.14)***	-0.49 (0.15)***	-0.32 (0.18)*	-0.12 (0.19)	-0.07 (0.19)	-0.32 (0.17)*	-0.96 (0.18)***	-0.40 (0.16)**
6th	-0.64 (0.13)***	-0.45 (0.14)***	-0.26 (0.17)	-0.04 (0.18)	0.02 (0.18)	-0.21 (0.15)	-0.84 (0.16)***	-0.33 (0.15)**
7th	-0.60 (0.13)***	-0.41 (0.14)***	-0.20 (0.17)	0.04 (0.19)	0.11 (0.18)	-0.11 (0.15)	-0.72 (0.16)***	-0.27 (0.11)**
8th	-0.57 (0.13)***	-0.36 (0.14)**	-0.13 (0.18)	0.13 (0.20)	0.23 (0.20)	0.02 (0.18)	-0.58 (0.19)***	-0.22 (0.11)**
9th	-0.53 (0.14)***	-0.31 (0.15)**	-0.05 (0.20)	0.23 (0.22)	0.35 (0.24)	0.16 (0.23)	-0.43 (0.24)*	-0.15 (0.06)**
Highest 10%	-0.47 (0.15)***	-0.23 (0.17)	0.06 (0.23)	0.37 (0.27)	0.52 (0.30)	0.35 (0.31)	-0.22 (0.34)	-0.11 (0.07)
	Men							
Lowest 10%	-0.87 (0.19)***	-0.79 (0.23)***	-0.83 (0.32)***	-0.81 (0.39)**	-0.91 (0.45)**	-1.24 (0.50)**	-1.91 (0.55)***	-0.84 (0.30)***
2nd	-0.81 (0.17)***	-0.71 (0.19)***	-0.71 (0.27)***	-0.66 (0.32)**	-0.73 (0.36)**	-1.03 (0.39)***	-1.68 (0.43)***	-0.76 (0.23)***
3rd	-0.75 (0.15)***	-0.63 (0.16)***	-0.59 (0.22)***	-0.50 (0.25)**	-0.53 (0.27)*	-0.81 (0.28)***	-1.44 (0.32)***	-0.59 (0.21)***
4th	-0.69 (0.13)***	-0.56 (0.14)***	-0.48 (0.18)***	-0.36 (0.20)*	-0.36 (0.21)*	-0.62 (0.20)***	-1.24 (0.23)***	-0.54 (0.16)***
5th	-0.66 (0.13)***	-0.51 (0.13)***	-0.41 (0.17)**	-0.27 (0.18)	-0.25 (0.18)	-0.50 (0.16)***	-1.10 (0.19)***	-0.40 (0.16)**
6th	-0.63 (0.12)***	-0.48 (0.13)***	-0.35 (0.16)**	-0.19 (0.17)	-0.17 (0.17)	-0.40 (0.14)***	-0.99 (0.17)***	-0.42 (0.14)***
7th	-0.60 (0.12)***	-0.44 (0.13)***	-0.30 (0.16)*	-0.12 (0.17)	-0.08 (0.17)	-0.31 (0.14)**	-0.89 (0.16)***	-0.28 (0.13)**
8th	-0.57 (0.12)***	-0.40 (0.13)***	-0.23 (0.17)	-0.04 (0.18)	0.02 (0.18)	-0.19 (0.17)	-0.76 (0.19)***	-0.29 (0.12)**
9th	-0.53 (0.13)***	-0.35 (0.14)**	-0.16 (0.18)	0.06 (0.21)	0.14 (0.22)	-0.06 (0.21)	-0.62 (0.23)***	-0.16 (0.09)*
Highest 10%	-0.49 (0.14)***	-0.28 (0.15)*	-0.06 (0.21)	0.19 (0.25)	0.29 (0.28)	0.11 (0.29)	-0.43 (0.31)	-0.16 (0.10)

Values are derived at median intake levels by demographic subgroup. Standard errors are in (parenthesis). Population weights by sex, age, and income status were obtained from the World Development Indicators Data Bank: https://databank.worldbank.org/data/reports.aspx?source=world-development-indicators#.

*p≤0.10; **p≤0.05; ***p≤0.01.

†Price elasticities are based on 1% price changes. For instance, given a 1% SSB price increase in the lowest income countries, intake by women, age 20 falls by 0.90%. ‡Income deciles are based on the national income of the 164 countries included in the study. Each decile is comprised of 16 countries (except the 4 lowest deciles, which are each comprised of 17 countries). The per capita income range (PPP-adjusted in thousand $US) for each decile: (1st) $0.6-$1.5, (2nd) $1.5-$2.7, (3rd) $2.7-$5.3, (4th) $5.5-$8.0, (5th) $8.3-$10.8, (6th) $11.1-$15.2, (7th) $15.3-$20.3, (8th) $20.6-$29.4, (9th) $30.4-$40.9, and (10th) $41.3-$127.2.

For peer review only - http://bmjopen.bmj.com/site/about/guidelines.xhtml

Table 2 Potential impact of a 20% tax (price increase) on SSB intake by age, sex, and global income decile.

Income decile†	Women age < 35	Men age < 35	Women 35 ≤ age < 60	Men 35 ≤ age < 60	Women age ≥ 60	Men age ≥ 60
	Percentage change in intake (95% CI)					
Lowest 10%	-17.1 (-26.1 to -8.1)	-16.8 (-25.0 to -8.6)	-14.5 (-29.5 to 0.4)	-15.9 (-29.5 to -2.2)	-22.3 (-43.2 to -1.4)	-24.9 (-44.4 to -5.3)
2nd	-15.6 (-23.3 to -7.9)	-15.4 (-22.5 to -8.3)	-11.6 (-23.8 to 0.7)	-13.2 (-24.3 to -1.9)	-17.7 (-33.9 to -1.4)	-20.6 (-35.9 to -5.3)
3rd	-14.0 (-20.6 to -7.4)	-14.0 (-20.1 to -7.8)	-8.5 (-18.3 to 1.3)	-10.4 (-19.4 to -1.4)	-13.0 (-24.8 to -1.2)	-16.3 (-27.4 to -5.1)
4th	-12.6 (-18.5 to -6.7)	-12.7 (-18.2 to -7.3)	-5.9 (-14.0 to 2.2)	-8.0 (-15.5 to -0.5)	-9.0 (-17.3 to -0.7)	-12.5 (-20.5 to -4.6)
5th	-11.7 (-17.3 to -6.2)	-11.9 (-17.1 to -6.7)	-4.2 (-11.6 to 3.2)	-6.5 (-13.2 to 0.3)	-6.3 (-12.9 to 0.3)	-10.1 (-16.4 to -3.7)
6th	-11.0 (-16.4 to -5.6)	-11.3 (-16.3 to -6.2)	-2.8 (-9.9 to 4.3)	-5.2 (-11.6 to 1.3)	-4.2 (-10 to 1.7)	-8.1 (-13.7 to -2.4)
7th	-10.3 (-15.7 to -5.0)	-10.6 (-15.6 to -5.7)	-1.4 (-8.5 to 5.6)	-4.0 (-10.4 to 2.5)	-2.1 (-8 to 3.9)	-6.2 (-11.8 to -0.4)
8th	-9.5 (-14.9 to -4.1)	-9.9 (-14.9 to -4.9)	0.2 (-7.3 to 7.6)	-2.5 (-9.2 to 4.3)	0.4 (-6.6 to 7.5)	-3.8 (-10.4 to 2.8)
9th	-8.5 (-14.2 to -2.9)	-9.0 (-14.3 to -3.8)	2.0 (-6.3 to 10.3)	-0.8 (-8.3 to 6.7)	3.2 (-5.8 to 12.3)	-1.2 (-9.6 to 7.2)
Highest 10%	-7.3 (-13.5 to -1.1)	-7.9 (-13.6 to -2.2)	4.5 (-5.4 to 14.3)	1.4 (-7.6 to 10.3)	7.0 (-5.3 to 19.3)	2.2 (-9.1 to 13.6)

Values are reductions from median intake levels for each demographic subgroup.
†Income deciles are based on the national income of the 164 countries included in the study. Each decile is comprised of 16 countries except the 4 lowest deciles, which are each comprised of 17 countries. The per capita income range (PPP-adjusted in thousand $US) for each decile: (1st) $0.6-$1.5, (2nd) $1.5-$2.7, (3rd) $2.7-$5.3, (4th) $5.5-$8.0, (5th) $8.3-$10.8, (6th) $11.1-$15.2, (7th) $15.3-$20.3, (8th) $20.6-$29.4, (9th) $30.4-$40.9, and (10th) $41.3-$127.2.

For peer review only

<Insert Figure1.png file here>

Figure 1 Comparison of mean SSB intakes among adults in age, sex, and country-specific strata across world regions and globally by select age groups. *n* represents the number of age, sex, and country-specific subgroups in each stratum. Boxes represent the median intake value and interquartile range; error bars represent the minimum and maximum values. Source: Global Dietary Database, 2010

For peer review only

For peer review only - http://bmjopen.bmj.com/site/about/guidelines.xhtml

<Insert Figure2.png file here>

Figure 2 Global SSB own-price elasticities by age, sex, and global income decile. Values are derived at median intake levels by demographic subgroup. Own-price elasticities are based on 1% price changes. Income deciles are based on the national income of the 164 countries included in the study. Each decile is comprised of 16 countries (except the 4 lowest deciles, which are each comprised of 17 countries). The per capita income range (PPP-adjusted in thousand $US) for each decile: (1st) $0.6-$1.5, (2nd) $1.5-$2.7, (3rd) $2.7-$5.3, (4th) $5.5-$8.0, (5th) $8.3-$10.8, (6th) $11.1-$15.2, (7th) $15.3-$20.3, (8th) $20.6-$29.4, (9th) $30.4-$40.9, and (10th) $41.3-$127.2.

For peer review only

For peer review only - http://bmjopen.bmj.com/site/about/guidelines.xhtml

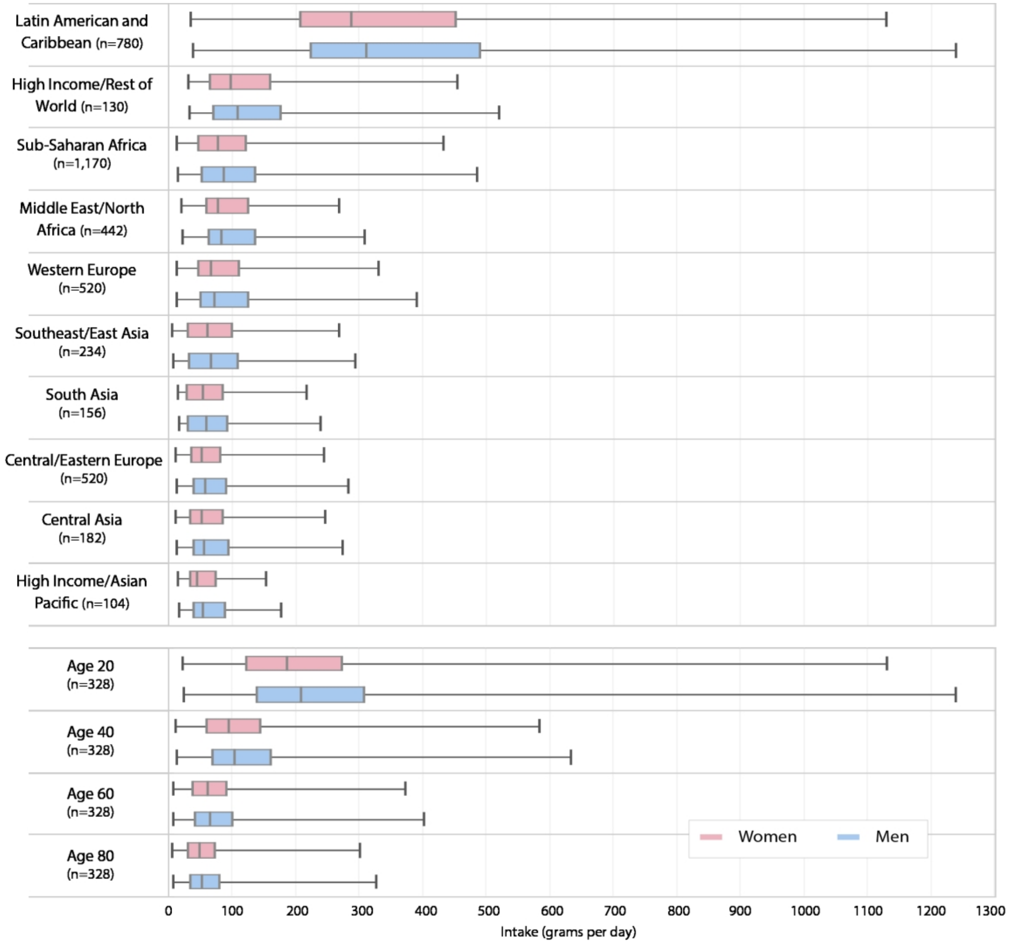

Figure 1 Comparison of mean SSB intakes among adults in age, sex, and country-specific strata across world regions and globally by select age groups. n represents the number of age, sex, and country-specific subgroups in each stratum. Boxes represent the median intake value and interquartile range; error bars represent the minimum and maximum values.
Source: Global Dietary Database, 2010

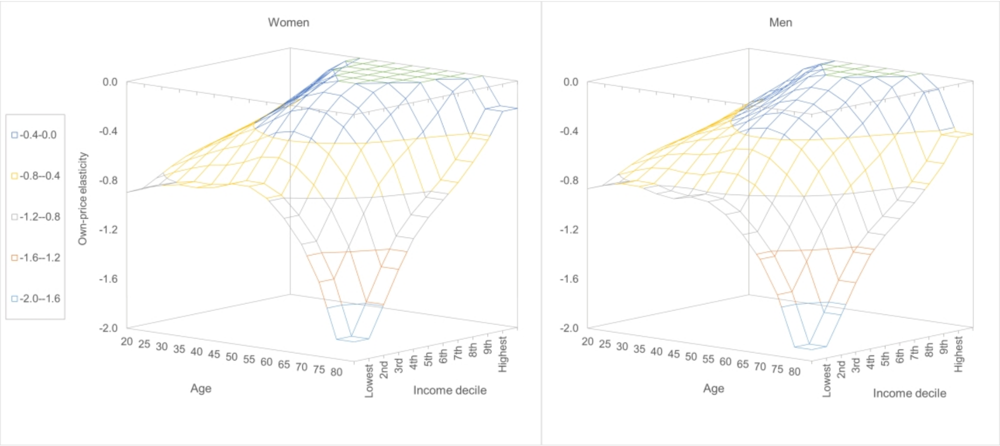

Figure 2 Global SSB own-price elasticities by age, sex, and global income decile. Values are derived at median intake levels by demographic subgroup. Own-price elasticities are based on 1% price changes. Income deciles are based on the national income of the 164 countries included in the study. Each decile is comprised of 16 countries (except the 4 lowest deciles, which are each comprised of 17 countries). The per capita income range (PPP-adjusted in thousand $US) for each decile: (1st) $0.6-$1.5, (2nd) $1.5-$2.7, (3rd) $2.7-$5.3, (4th) $5.5-$8.0, (5th) $8.3-$10.8, (6th) $11.1-$15.2, (7th) $15.3-$20.3, (8th) $20.6-$29.4, (9th) $30.4-$40.9, and (10th) $41.3-$127.2.

Supplementary Information

Global patterns in price elasticities of sugar-sweetened beverage intake and potential effectiveness of tax policy: results from 164 countries

Andrew Muhammad,[1] Birgit Meade,[2] David R Marquardt,[2] and Dariush Mozaffarian[3]

[1]University of Tennessee Institute of Agriculture, Department of Agricultural and Resource Economics, Knoxville, Tennessee, USA
[2]United States Department of Agriculture, Economic Research Service, Market and Trade Economics Division, Washington, D.C., USA
[3]Tufts University, Friedman School of Nutrition Science and Policy, Boston, Massachusetts, USA

Supplemental Figure 1	Mean SSB intake across countries in adults, age ≥ 20
Supplemental Table 1	Description of ICP food-price categories
Supplemental Table 2	Countries included in study by region (aggregate regions used for estimation)
Supplemental Table 3	Demand model estimates for SSB intake
Supplemental Table 4	Own-price elasticities of SSB intake by age, sex, and global income decile
Technical Appendix	Demand model and methods
Supplemental Figure 2	Deflated sugar price index: unadjusted and adjusted

For peer review only

Supplementary Information

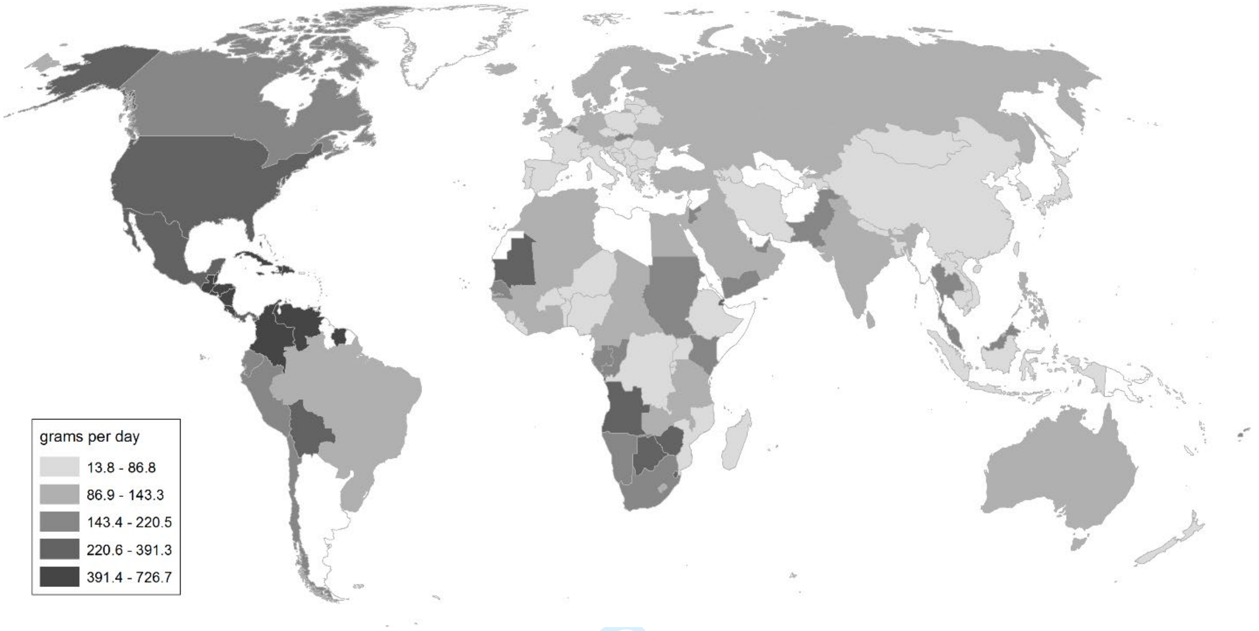

Supplemental Figure 1

Mean SSB intake across countries in adults, age ≥ 20

Source: Global Dietary Database, 2010

eview only

Supplemental Table 1

Description of ICP food-price categories

ICP food-price category
• *Fresh or chilled fruit* – All fresh or chilled fruit including melons and water melons; excludes vegetables grown for their fruit such as cucumbers and tomatoes.
• *Fresh milk* – Raw milk; pasteurised or sterilised milk; includes whole and low fat milk; recombined or reconstituted milk; soya milk.
• *Sugar* – Cane or beet sugar, unrefined or refined, powdered, crystallised or in lumps; includes artificial sugar substitutes.

For peer review only

Supplemental Table 2

Countries included in study by region (aggregate regions used for estimation)

Region	Countries
Southeast Asia, East Asia, and High Income Asia Pacific (Asia) (13 countries)	Brunei Darussalam, Cambodia, China, Indonesia, Japan, South Korea, Laos, Malaysia, Maldives, Philippines, Singapore, Thailand, and Vietnam.
Central Europe, Eastern Europe, and Central Asia (CEE/C. Asia) (27 countries)	Albania, Armenia, Azerbaijan, Belarus, Bosnia and Herzegovina, Bulgaria, Croatia, Czech Republic, Estonia, Georgia, Hungary, Kazakhstan, Kyrgyz Republic, Latvia, Lithuania, Macedonia, Moldova, Mongolia, Montenegro, Poland, Romania, Russia, Serbia, Slovak Republic, Slovenia, Tajikistan, and Ukraine.
Latin America and the Caribbean (LAC) (30 countries)	Antigua and Barbuda, Bahamas, Barbados, Belize, Bolivia, Brazil, Chile, Colombia, Costa Rica, Cuba, Dominica, Dominican Republic, Ecuador, El Salvador, Grenada, Guatemala, Haiti, Honduras, Jamaica, Mexico, Nicaragua, Panama, Paraguay, Peru, St. Lucia, St. Vincent and Grenadines, Suriname, Trinidad and Tobago, Uruguay, and Venezuela.
Middle East, North Africa, and South Asia (MENA/S. Asia) (23 countries)	Algeria, Bahrain, Bangladesh, Bhutan, Egypt, India, Iran, Iraq, Israel, Jordan, Kuwait, Morocco, Nepal, Oman, Pakistan, Qatar, Saudi Arabia, Sri Lanka, Tunisia, Turkey, United Arab Emirates, West Bank and Gaza, and Yemen.
Sub-Saharan Africa (SSA) (45 countries)	Angola, Benin, Botswana, Burkina Faso, Burundi, Cameroon, Cape Verde, Central African Republic, Chad, Comoros, Congo, Côte d'Ivoire, Djibouti, DR Congo, Equatorial Guinea, Ethiopia, Gabon, Gambia, Ghana, Guinea, Guinea-Bissau, Kenya, Lesotho, Liberia, Madagascar, Malawi, Mali, Mauritania, Mauritius, Mozambique, Namibia, Niger, Nigeria, Rwanda, São Tomé and Principe, Senegal, Sierra Leone, South Africa, Sudan, Swaziland, Tanzania, Togo, Uganda, Zambia, and Zimbabwe.
High Income/Rest of World (HIC) (26 countries)	Australia, Austria, Belgium, Canada, Cyprus, Denmark, Fiji, Finland, France, Germany, Greece, Iceland, Ireland, Italy, Luxembourg, Malta, Netherlands, New Zealand, Norway, Portugal, Seychelles, Spain, Sweden, Switzerland, United Kingdom, and United States.

For peer review only

Supplemental Table 3

Demand model estimates for SSB intake

Variable	model 1 estimate (SE)	model 2 estimate (SE)	model 3 (final model) estimate (SE)
constant	436.63 (25.47)***	-784.78 (378.46)**	-1,398.66 (535.82)***
female (F)	-13.36 (0.82)***	-13.36 (0.82)***	28.17 (15.92)*
age	-10.87 (0.74)***	-10.87 (0.74)***	14.06 (11.51)
age^2	0.08 (0.01)***	0.08 (0.01)***	-0.15 (0.09)*
SSA	1.83 (11.47)	35.51 (17.22)**	78.19 (37.85)**
LAC	258.41 (26.38)***	251.12 (28.01)***	546.89 (62.00)***
MENA/S. Asia	-10.05 (10.57)	7.45 (13.56)	21.20 (28.71)
CEE/C. Asia	-32.87 (9.99)***	-18.44 (12.59)	-28.38 (26.74)
Asia	-26.85 (14.66)*	-59.53 (19.50)***	-121.47 (41.21)***
age × SSA			-0.85 (0.42)**
age × LAC			-5.92 (0.69)***
age × MENA/S. Asia			-0.28 (0.32)
age × CEE/C. Asia			0.20 (0.30)
age × Asia			1.24 (0.45)***
$\log(P_s)$		-42.65 (15.23)***	-483.47 (116.99)***
$F \times \log(P_s)$			12.42 (2.33)***
$Age \times \log(P_s)$			9.89 (1.20)**
$Age^2 \times \log(P_s)$			-0.09 (0.01)***
$\log(P_f)$		85.90 (25.27)***	231.34 (83.87)***
$F \times \log(P_f)$			-1.38 (2.77)
$Age \times \log(P_f)$			-4.15 (2.22)*
$Age^2 \times \log(P_f)$			0.02 (0.02)
$\log(P_m)$		53.30 (20.73)***	107.80 (72.51)
$F \times \log(P_m)$			1.57 (2.63)
$Age \times \log(P_m)$			-0.97 (1.98)
$Age^2 \times \log(P_m)$			0.00 (0.02)
$\log(Y)$		145.85 (45.34)***	379.56 (109.52)***
$F \times \log(Y)$			-6.23 (2.17)***
$Age \times \log(Y)$			-3.49 (1.53)**
$Age^2 \times \log(Y)$			0.03 (0.01)***
$\log(Y)^2$		-8.77 (3.04)***	-18.97 (6.48)***
$\log(P_s) \times \log(Y)$			19.87 (10.73)*
Adjusted R^2	0.65	0.70	0.80

Note: Dependent variable is SSB intake in g/d. Standard errors are in (parentheses).
$^{*}p \leq 0.10$; $^{**}p \leq 0.05$; $^{***}p \leq 0.01$. SSA = Sub-Saharan Africa. LAC = Latin America and the Caribbean. MENA/S. Asia = Middle East, North Africa, and South Asia. CEE/C. Asia = Central Europe, Eastern Europe, and Central Asia. Asia = Asian Pacific, East Asia, and Southeast Asia. The reference region consist of high-income Western countries and a few small island states. P_s = SSB price, P_f = fruit juice price, P_m = milk price. All prices were deflated by a food price index. Y = real per capita income.

For peer review only - http://bmjopen.bmj.com/site/about/guidelines.xhtml

Supplemental Table 4

Own-price elasticities of SSB intake by age, sex, and global income decile

Income decile	Age 20	25	30	35	40	45	50	55	60	65	70	75	80
	Women												
Lowest	-0.90	-0.84	-0.80	-0.77	-0.78	-0.76	-0.70	-0.71	-0.78	-0.92	-1.11	-1.40	-1.84
10%	(0.21)	(0.23)	(0.25)	(0.29)	(0.35)	(0.40)	(0.42)	(0.45)	(0.49)	(0.52)	(0.53)	(0.55)	(0.60)
2nd	-0.83	-0.76	-0.71	-0.67	-0.65	-0.60	-0.54	-0.52	-0.58	-0.70	-0.88	-1.16	-1.59
	(0.18)	(0.19)	(0.21)	(0.24)	(0.29)	(0.33)	(0.34)	(0.36)	(0.38)	(0.41)	(0.41)	(0.43)	(0.46)
3rd	-0.76	-0.69	-0.62	-0.56	-0.51	-0.44	-0.36	-0.33	-0.37	-0.48	-0.65	-0.92	-1.33
	(0.16)	(0.17)	(0.18)	(0.20)	(0.23)	(0.26)	(0.27)	(0.28)	(0.29)	(0.30)	(0.30)	(0.31)	(0.34)
4th	-0.70	-0.62	-0.54	-0.47	-0.40	-0.31	-0.22	-0.17	-0.19	-0.28	-0.45	-0.71	-1.10
	(0.14)	(0.15)	(0.16)	(0.17)	(0.20)	(0.21)	(0.22)	(0.22)	(0.22)	(0.22)	(0.21)	(0.21)	(0.24)
5th	-0.67	-0.58	-0.49	-0.40	-0.32	-0.22	-0.12	-0.07	-0.07	-0.15	-0.32	-0.57	-0.96
	(0.14)	(0.14)	(0.15)	(0.16)	(0.18)	(0.20)	(0.19)	(0.19)	(0.19)	(0.18)	(0.17)	(0.16)	(0.18)
6th	-0.64	-0.54	-0.45	-0.35	-0.26	-0.14	-0.04	0.02	0.02	-0.05	-0.21	-0.46	-0.84
	(0.13)	(0.13)	(0.14)	(0.15)	(0.17)	(0.19)	(0.18)	(0.18)	(0.18)	(0.17)	(0.15)	(0.14)	(0.16)
7th	-0.60	-0.51	-0.41	-0.31	-0.20	-0.07	0.04	0.10	0.11	0.05	-0.11	-0.35	-0.72
	(0.13)	(0.13)	(0.14)	(0.15)	(0.17)	(0.19)	(0.19)	(0.18)	(0.18)	(0.17)	(0.15)	(0.14)	(0.16)
8th	-0.57	-0.46	-0.36	-0.25	-0.13	0.01	0.13	0.20	0.23	0.17	0.02	-0.22	-0.58
	(0.13)	(0.13)	(0.14)	(0.16)	(0.18)	(0.20)	(0.20)	(0.20)	(0.20)	(0.19)	(0.18)	(0.17)	(0.19)
9th	-0.53	-0.42	-0.31	-0.18	-0.05	0.11	0.23	0.32	0.35	0.31	0.16	-0.08	-0.43
	(0.14)	(0.14)	(0.15)	(0.17)	(0.20)	(0.22)	(0.22)	(0.23)	(0.24)	(0.24)	(0.23)	(0.23)	(0.24)
Highest	-0.47	-0.36	-0.23	-0.10	0.06	0.23	0.37	0.47	0.52	0.49	0.35	0.12	-0.22
10%	(0.15)	(0.16)	(0.17)	(0.19)	(0.23)	(0.26)	(0.27)	(0.29)	(0.30)	(0.32)	(0.31)	(0.32)	(0.34)
	Men												
Lowest	-0.87	-0.82	-0.79	-0.79	-0.83	-0.85	-0.81	-0.83	-0.91	-1.06	-1.24	-1.50	-1.91
10%	(0.19)	(0.21)	(0.23)	(0.26)	(0.32)	(0.37)	(0.39)	(0.42)	(0.45)	(0.48)	(0.50)	(0.51)	(0.55)
2nd	-0.81	-0.75	-0.71	-0.69	-0.71	-0.70	-0.66	-0.66	-0.73	-0.85	-1.03	-1.28	-1.68
	(0.17)	(0.18)	(0.19)	(0.22)	(0.27)	(0.31)	(0.32)	(0.34)	(0.36)	(0.38)	(0.39)	(0.4)	(0.43)
3rd	-0.75	-0.68	-0.63	-0.60	-0.59	-0.56	-0.50	-0.48	-0.53	-0.64	-0.81	-1.06	-1.44
	(0.15)	(0.15)	(0.16)	(0.18)	(0.22)	(0.25)	(0.25)	(0.26)	(0.27)	(0.28)	(0.28)	(0.29)	(0.32)
4th	-0.69	-0.62	-0.56	-0.51	-0.48	-0.43	-0.36	-0.33	-0.36	-0.46	-0.62	-0.87	-1.24
	(0.13)	(0.14)	(0.14)	(0.16)	(0.18)	(0.20)	(0.20)	(0.20)	(0.21)	(0.21)	(0.20)	(0.21)	(0.23)
5th	-0.66	-0.58	-0.51	-0.46	-0.41	-0.34	-0.27	-0.23	-0.25	-0.34	-0.50	-0.74	-1.10
	(0.13)	(0.13)	(0.13)	(0.15)	(0.17)	(0.18)	(0.18)	(0.18)	(0.18)	(0.17)	(0.16)	(0.16)	(0.19)
6th	-0.63	-0.55	-0.48	-0.41	-0.35	-0.28	-0.19	-0.15	-0.17	-0.25	-0.40	-0.64	-0.99
	(0.12)	(0.13)	(0.13)	(0.14)	(0.16)	(0.18)	(0.17)	(0.17)	(0.17)	(0.16)	(0.14)	(0.14)	(0.17)
7th	-0.60	-0.52	-0.44	-0.37	-0.30	-0.21	-0.12	-0.07	-0.08	-0.16	-0.31	-0.54	-0.89
	(0.12)	(0.12)	(0.13)	(0.14)	(0.16)	(0.18)	(0.17)	(0.17)	(0.17)	(0.16)	(0.14)	(0.14)	(0.16)
8th	-0.57	-0.48	-0.40	-0.32	-0.23	-0.13	-0.04	0.02	0.02	-0.04	-0.19	-0.42	-0.76
	(0.12)	(0.13)	(0.13)	(0.15)	(0.17)	(0.18)	(0.18)	(0.18)	(0.18)	(0.18)	(0.17)	(0.17)	(0.19)
9th	-0.53	-0.44	-0.35	-0.26	-0.16	-0.04	0.06	0.13	0.14	0.08	-0.06	-0.29	-0.62
	(0.13)	(0.13)	(0.14)	(0.16)	(0.18)	(0.20)	(0.21)	(0.21)	(0.22)	(0.22)	(0.21)	(0.21)	(0.23)
Highest	-0.49	-0.39	-0.28	-0.18	-0.06	0.07	0.19	0.27	0.29	0.25	0.11	-0.11	-0.43
10%	(0.14)	(0.14)	(0.15)	(0.18)	(0.21)	(0.24)	(0.25)	(0.27)	(0.28)	(0.29)	(0.29)	(0.29)	(0.31)

Note: Values are derived at median intake levels by demographic subgroup. Standard errors are in (parentheses). Price elasticities are based on 1% price changes. Income deciles are based on the national income of the 164 countries included in the study. Each decile is comprised of 16 countries except the 4 lowest deciles, which are each comprised of 17 countries. The per capita income range (PPP-adjusted in thousand $US) for each decile: (1st) $0.6-$1.5, (2nd) $1.5-$2.7, (3rd) $2.7-$5.3, (4th) $5.5-$8.0, (5th) $8.3-$10.8, (6th) $11.1-$15.2, (7th) $15.3-$20.3, (8th) $20.6-$29.4, (9th) $30.4-$40.9, and (10th) $41.3-$127.2.

For peer review only - http://bmjopen.bmj.com/site/about/guidelines.xhtml

Technical Appendix

Demand model and methods

To estimate SSB intake demand, we used a semi-logarithmic functional form that has been proven to be consistent with economic theory and rational consumer behavior.(1, 2) We applied a single-equation framework in this study. Prior studies have used a demand-system approach (multi-equation framework), primarily due to the adding-up property when using expenditure data (i.e., expenditures on all consumption categories "add up" to total expenditures), which results in the error terms being correlated across categories. Since this relationship does not exist with individual intakes, particularly when the correspondence between purchases and intakes is not one to one, the adopted approach is acceptable.

Many studies have used a double-log quadratic form.(3) However, a problem with the double-log form is that significant intake differences across subgroups can be lost in log conversions. A semi-log relationship allowed for a better assessment of subgroup effects on intake responsiveness. It has also been shown that semi-log models of demand are consistent with economic theory and contain the necessary information for obtaining, for instance, reliable measures of consumer welfare and the underlying preference structure of consumers.(1)

Let q_{ig} represent SSB intake by subgroup g (g: sex and age), p_i and p_j represent the price of SSBs and related good j, and Y and P represent real per capita income and overall food prices (all in country C). SSB intake demand by subgroup g in country C is specified as follows (C subscripts are omitted for convenience):

$$q_{ig} = \beta_0^* + \beta_1^* \ln(Y) + \beta_2^* \ln\left(\frac{p_i}{P}\right) + \beta_3^* \ln\left(\frac{p_j}{P}\right) + \beta_4^* \left[\ln(Y) \times \ln\left(\frac{p_i}{P}\right)\right] + \beta_5^* \ln(Y)^2 + u_{ig} \quad (1)$$

The β terms are coefficients to be estimated and u_{ig} is a random error term. The price terms (p_i and p_j) are deflated by P to discount price differences due to overall food prices and to implicitly account for the cross-price effects of intake categories other than i and j. Note that the structure of the model allows for the relationship between own-price (p_i) and intake to vary by national income level.

We accounted for age, sex, and regional differences by allowing these factors to have a direct effect on intake, as well as an additional effect through income and prices. Thus, the beta terms (β_i^*) were expanded to account for age, sex, and region interactions.

$$\beta_i^* = f(sex, age, region); i = 0,1,\ldots,5 \quad (2)$$

The variable *sex* is a binary (= 1 for women and 0 otherwise) and *age* is a variable ranging from 20 to 80 in 5-year intervals. We also considered age^2 to allow for nonlinear age effects and the possibility of optimal responsiveness between the youngest and oldest subgroup. We accounted for varying preferences across countries due to factors not related to income or prices by including six regional binary variables, including: Southeast Asia, East Asia, and High Income Asia Pacific (Asia) (13 countries); Central Europe, Eastern Europe, and Central Asia (CEE/C. Asia) (27); Latin America and the Caribbean (LAC) (30); Middle East, North Africa, and South Asia (MENA/S. Asia) (23); Sub-Saharan Africa (SSA) (45); and High Income/Rest of World (HIC) (26). HIC was comprised largely of Western, industrialized countries; while not geographically connected, these countries share other similarities. We included several small

For peer review only - http://bmjopen.bmj.com/site/about/guidelines.xhtml

island countries in this grouping because they were not sufficiently numerous to merit their own regional grouping.

We first estimated a model with all possible interactions and then utilized F-tests to compare that model to a series of restricted models and arrived at the final parsimonious model. All models were estimated assuming country clusters, that is, independent errors across countries but correlated errors within countries, as well as heteroskedastic-consistent errors.(4)

Given equation (1), the own-price elasticity is derived as follows:

$$\eta_{iig} = \frac{\%\Delta q_{ig}}{\%\Delta p_i} = \frac{1}{q_{ig}}[\beta_2^* + \beta_4^* \ln(Y)] \quad (3)$$

η_{iig} is the percentage change in intake (q_{ig}) (i: SSB) due to a 1% change in p_i, which should be negative since an increase in price usually results in a decrease in intake or quantity demanded. Note that if the β coefficients vary with sex, age, or region, equation (3) will vary accordingly.

Price index adjustment

We used sugar and fresh fruit price indexes as proxies for SSB and fruit juice prices, respectively. An issue with this approach is that sugar and/or fresh fruit may not account for a major share of the final product price, particularly in higher income countries. In view of this, we adjusted the sugar and fresh fruit price indexes with national income level assuming the following quadratic relationship between the price adjustment and real per capita income.

$$Price\ Adustment = a + b\ Income + c\ Income^2 \quad (4)$$

We used a calibration method to derive values for a, b, and c. First, we considered the extreme case, $Income = 0$ and set $a = 1$, which allowed for the index to remained relatively unchanged at low income levels. Using information on the value-added share of farm products in the U.S. food and beverage sector we obtained the following: $b = 0.0003$ and $c = 0.0000000015$. We applied this adjustment to sugar and fresh fruit prices in all countries. The unadjusted and adjusted sugar price indexes are reported in supplemental figure 2.

References

1. LaFrance JT. Incomplete demand systems and semilogarithmic demand models. Australian J Agr Econ. 1990;34(2):118-31.
2. von Haefen RH. A complete characterization of the linear, log-linear, and semi-log incomplete demand system models. J Agr Resource Econ. 2002;27(2):281-319.
3. Sahn DE. The Effect of Price and Income Changes on Food-Energy Intake in Sri Lanka. Econ Devel Cult Change. 1988;36(2):315-40.
4. Cameron AC, Trivedi PK. Microeconometrics: Methods and Applications. Cambridge: Cambridge University Press; 2005.

For peer review only - http://bmjopen.bmj.com/site/about/guidelines.xhtml

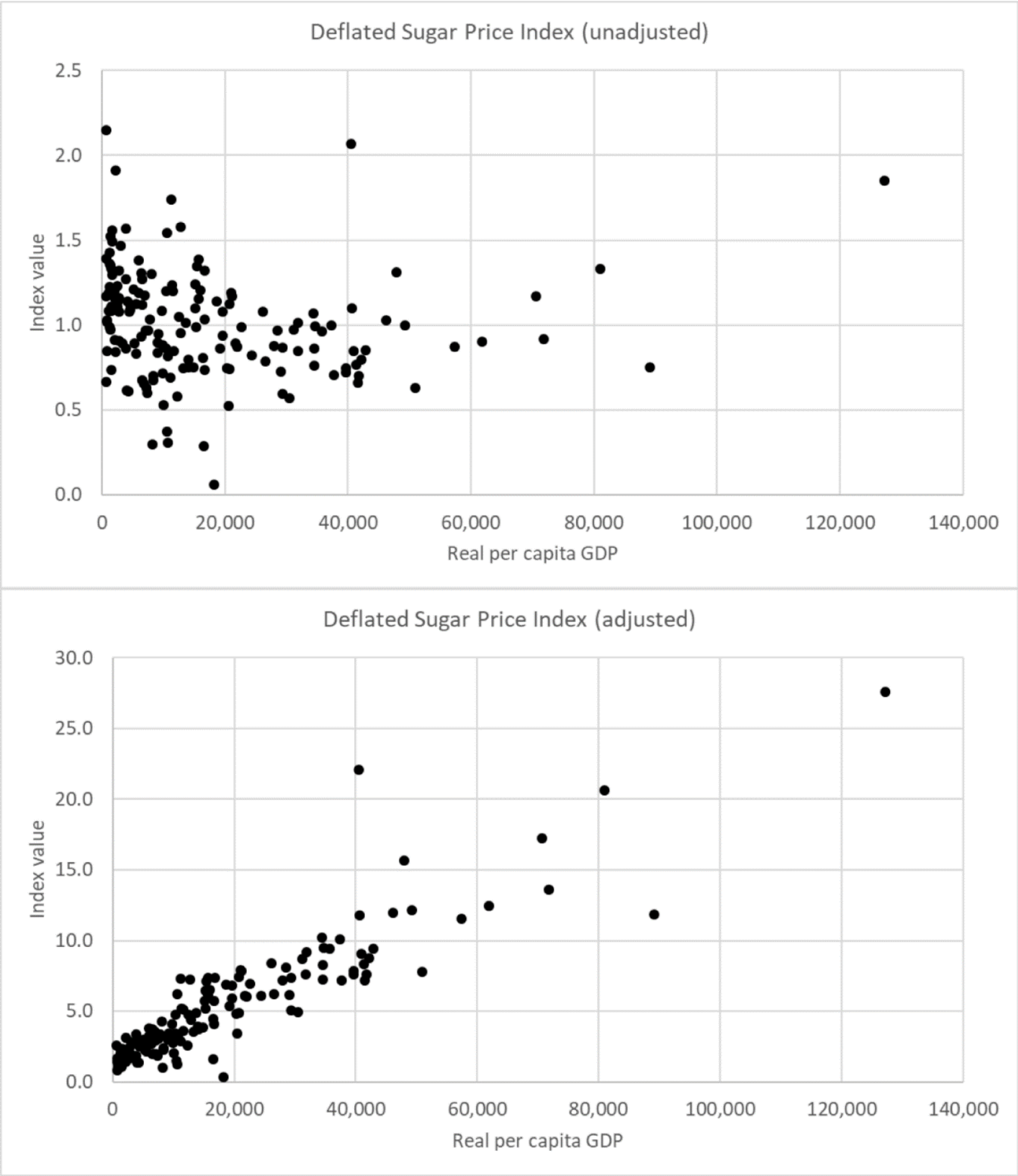

Supplemental Figure 2

Deflated sugar price index: unadjusted and adjusted

Note: prices are deflated by a total food price index to discount differences across countries due to overall food prices.

Global patterns in price elasticities of sugar-sweetened beverage intake and potential effectiveness of tax policy: a cross-sectional study of 164 countries by sex, age, and global-income decile

Journal:	*BMJ Open*
Manuscript ID	bmjopen-2018-026390.R2
Article Type:	Research
Date Submitted by the Author:	21-Mar-2019
Complete List of Authors:	Muhammad, Andrew; University of Tennessee Institute of Agriculture<br>Meade, Birgit; USDA Economic Research Service<br>Marquardt, David; USDA Economic Research Service<br>Mozaffarian, Dariush; Tufts University Friedman School of Nutrition Science and Policy
**Primary Subject Heading**:	Global health
Secondary Subject Heading:	Health economics, Health policy
Keywords:	elasticity, intake, global, prices, sugar-sweetened beverages, taxes

SCHOLARONE™
Manuscripts

Global patterns in price elasticities of sugar-sweetened beverage intake and potential effectiveness of tax policy: a cross-sectional study of 164 countries by sex, age, and global-income decile

Andrew Muhammad,[1] Birgit Meade,[2] David R Marquardt,[2] and Dariush Mozaffarian[3]

(Corresponding author) Andrew Muhammad, University of Tennessee Institute of Agriculture, Department of Agricultural and Resource Economics, 2621 Morgan Circle, 302 Morgan Hall Knoxville, Tennessee 37996-4518, USA

Email: amuhammad@utk.edu

[1]University of Tennessee Institute of Agriculture, Department of Agricultural and Resource Economics, Knoxville, Tennessee, USA

[2]United States Department of Agriculture, Economic Research Service, Market and Trade Economics Division, Washington, D.C., USA

[3]Tufts University, Friedman School of Nutrition Science and Policy, Boston, Massachusetts, USA

Keywords: demand, diet, elasticity, intake, global, prices, sugar-sweetened beverages, taxes

Disclaimer: The views expressed are those of the authors, and may not be attributed to the Economic Research Service or the U.S. Department of Agriculture.

Copyright: The Corresponding Author has the right to grant on behalf of all authors and does grant on behalf of all authors, a worldwide licence (or non-exclusive for government employees) to the Publishers and its licensees in perpetuity, in all forms, formats and media (whether known now or created in the future), to i) publish, reproduce, distribute, display and store the Contribution, ii) translate the Contribution into other languages, create adaptations, reprints, include within collections and create summaries, extracts and/or, abstracts of the Contribution, iii) create any other derivative work(s) based on the Contribution, iv) to exploit all subsidiary rights in the Contribution, v) the inclusion of electronic links from the Contribution to third party material where-ever it may be located; and, vi) licence any third party to do any or all of the above.

For peer review only - http://bmjopen.bmj.com/site/about/guidelines.xhtml

Competing interests: We have read and understood BMJ policy on declaration of interests and declare that we have no competing interests.

Contributors: AM conceptualized the study and was responsible for the study design, model estimations, and contributed to the interpretation of results. AM, DM, and BM contributed to the interpretation of results and discussion. DM wrote and edited sections describing the intake data. BM was primarily responsible for the literature review and facilitated the data agreement with the International Comparison Program, World Bank. DM provided the intake data and obtained the funding. DRM and AM were responsible for the visualizations and corresponding text. AM was the primary author, but all authors contributed to writing the manuscript. AM is the manuscript's guarantor.

Data sharing: Real per capita income data were obtained from the World Development Indicators Data Bank. https://databank.worldbank.org/data/reports.aspx?source=world-development-indicators. SSB Intake data are available upon request from the Global Dietary Database. http://www.globaldietarydatabase.org/. Price indexes for select food categories are available upon request from the World Bank, International Comparison Program. icp@worldbank.org.

Transparency declaration: The lead author* affirms that this manuscript is an honest, accurate, and transparent account of the study being reported; that no important aspects of the study have been omitted; and that any discrepancies from the study as planned (and, if relevant, registered) have been explained. *The manuscript's guarantor.

Funding: The Bill & Melinda Gates Foundation (project: Global Dietary Habits among Women, Price and Income Elasticities, and Validity of Food Balance Sheets).

Role of the funding source: The funders had no role in the study design, study conduct, data analysis, data interpretation, or writing of the report. All authors had full access to all the data

For peer review only - http://bmjopen.bmj.com/site/about/guidelines.xhtml

and take responsibility for the integrity of the data and the accuracy of the analysis. The corresponding author had final responsibility to submit the report for publication.

For peer review only

For peer review only - http://bmjopen.bmj.com/site/about/guidelines.xhtml

Abstract

Objective – To quantify global relationships between sugar-sweetened beverage (SSB) intake and prices and examine the potential effectiveness of tax policy.

Design – SSB intake data by country, age, and sex from the Global Dietary Database were combined with gross domestic product (GDP) and price data from the World Bank International Comparison Program. Intake responsiveness to income and prices was estimated accounting for national income, age, and sex differences.

Setting – 164 countries.

Population – Full adult population in each country.

Main outcome measures – A consumer demand modeling framework was used to estimate the relationship between SSB intake and prices and derive own-price elasticities (measures of percentage changes in intake from a 1% price change) globally by age and sex. We simulated how a 20% tax would impact SSB intake globally. Tax policy outcomes were examined across countries by global income decile for representative age and sex subgroups.

Results – Own-price responsiveness was highest in lowest income countries, ranging from -0.70 (p<0.100) for women, age 50, to -1.91 (p<0.001) for men, age 80. In the highest income countries, responsiveness was as high as -0.49 (p<0.001) (men, age 20), but was mostly insignificant for older adults. Overall, elasticities were strongest (more negative) at the youngest and oldest age groups, and mostly insignificant for middle-aged adults, particularly in middle-income and high-income countries. Sex differences were mostly negligible. Potential intake reductions from a 20% tax in lowest income countries ranged from 14.5% (95% CI: 29.5%, -0.4%) in women, 35 ≤ age < 60, to 24.9% (44.4%, 5.3%) in men, age ≥ 60. Intake reductions decreased with country income overall, and were mostly insignificant for middle-aged adults.

Conclusions – These findings estimate the global price-responsiveness of SSB intake by age and sex, informing ongoing policy discussions on potential effects of taxes.

For peer review only

For peer review only - http://bmjopen.bmj.com/site/about/guidelines.xhtml

Strengths and limitations of this study

- First study to examine SSB intake and taxation in a global context, providing a better understanding of tax-policy effectiveness across the complete spectrum of countries.
- Results quantify the potential variability in influence of price on SSB intake across countries including by age and sex, suggesting that outcomes of SSB taxes may be significantly influenced by age and the income status of countries.
- Being a modeling study, the projected outcomes can only inform how taxes could affect behavior.
- Cross-country analysis of this scope rely on specific data collection initiatives that often do not occur on an annual basis and/or do not provide specific variables; proxy variables are needed when data are not available.

For peer review only

For peer review only - http://bmjopen.bmj.com/site/about/guidelines.xhtml

Introduction

Taxation of sugar-sweetened beverages (SSBs) has received growing attention, given their links to excessive weight gain and increased risk of obesity, type-2 diabetes, and other non-communicable diseases (NCDs).(1-5) Arguably, taxation is not punitive but market normalizing, as the true costs of SSBs due to public health-care expenditures and other societal costs from excessive intake are not reflected in current market prices. Thus, by increasing SSB prices relative to other foods, taxes can play a role in decreasing consumption, lowering societal costs, and improving societal wellbeing.(6, 7) Based on these considerations, a rapidly growing number of countries have implemented or announced national SSB taxes, (8, 9) including Norway in 1981 and Samoa in 1984; Australia, French Polynesia, Fiji, and Nauru between 2000 and 2007; and Finland, Hungary, France, Chile, Mexico, Barbados, St. Helena, and Dominica since 2011. In 2018, the Philippines, the United Kingdom, South Africa, the Republic of Ireland, Peru, and Norway implemented SSB taxes. Colombia and Saudi Arabia have included such taxes in recent proposals, while Bermuda, India and Indonesia are considering similar measures. In the U.S., more than 30 jurisdictions have implemented or attempted to pass SSB taxes since 2016, including San Francisco and Seattle in 2018.(10, 11) Despite their growing acceptance globally, the potential impact of SSB taxation on intake remains uncertain, particularly how it might vary across countries, and by age and sex within countries.

Most studies of SSB taxation have been limited to a small group of countries or focused on a specific country or jurisdiction where taxes have been implemented.(12-17) No study to date has examined SSB consumption and taxation in a global context. In addition, few studies have considered how SSB intake could vary depending on the price of substitute products.(18) Because expert organizations are advocating and governments are considering SSB taxation

For peer review only

For peer review only - http://bmjopen.bmj.com/site/about/guidelines.xhtml

across the globe,(19) examining demand in a global context can provide a better understanding of potential tax-policy effectiveness across the complete spectrum of countries, from most to least developed.

To investigate this issue, we examined SSB intake across 164 countries and estimated how intake differences within and across countries are influenced by the price of SSBs and substitute caloric beverages (fruit juice and milk), as well as other factors such as national income, age, and sex. Based on World Health Organization (WHO) recommendations,(19) we further simulated how SSB intake would respond to a 20% tax (price increase). Tax-policy outcomes were examined across countries by income decile for representative age and sex subgroups.

Methods

Using globally representative intake and pricing data, we implemented a consumer demand modeling framework to examine determinants of SSB intake within and across countries. The modeling framework accounted for age and sex differences and economic determinants such as own price, price of substitutes (fruit juice and milk), and real per capita income at the national level. We also considered the potential for unmeasured region-specific differences, such as taste or other preferences, by including regional binary variables. Model estimates were used to derive SSB own-price elasticities for detailed strata (age, sex, and countries by income decile), and to assess the potential impact of taxes on intake. Accounting for these factors, we report price elasticities of SSB intake (measures of the percentage change in intake from a 1% change in price), which have been a primary means of estimating potential tax-

For peer review only - http://bmjopen.bmj.com/site/about/guidelines.xhtml

policy effectiveness.(20) We also evaluated the variability in tax-policy effectiveness and examined outcomes for select age and sex subgroups and countries by income decile.

Data and sources

Data on SSB intake were derived from the 2010 Global Dietary Database (GDD), a database of global food and nutrient intakes by age (20-80 in 5-year intervals) and sex for 187 countries. The SSB category in the GDD includes intake of all sugar-sweetened beverages, including any beverage with added sugar and ≥ 50 kcal per 8 oz., such as carbonated beverages, sodas, energy drinks, fruit drinks, etc., excluding 100% juices. GDD data collection, statistical methods, data validation, and findings have been described in detail (also see http://www.globaldietarydatabase.org/).(21-25) In brief, GDD data were derived based on national and subnational dietary surveys, informed by additional information from United Nations Food and Agricultural Organization (FAO) food balance sheets data, individual-level surveys from cohort studies, household expenditure surveys when dietary surveys were not available, as well as other data sources such as the WHO Global Infobase and the WHO STEPS database.(25)

For prices, we used global price indices from the 2011 International Comparison Program (ICP) of the World Bank (see supplemental table 1).(26, 27) The ICP is a worldwide statistical initiative that produces price and expenditure data on consumer goods, services, and capital goods. The price indices used in this study are standardized to a common currency, the U.S. dollar in this case. Our choice of price variables was limited by inadequate data on a global scale. For instance, the ICP categories included milk but not SSBs and fruit juice. For SSBs, we used the ICP price index for sugar, which is justified, in part, due to sugar being a defining input.

For peer review only - http://bmjopen.bmj.com/site/about/guidelines.xhtml

Similarly, we used the ICP fresh or chilled fruit price index as a proxy for fruit juice prices. Since sugar or fresh fruit may not be a major share of the final product price, particularly in rich countries, there are limitations to these proxies. In view of this, we adjusted the sugar and fresh fruit price indexes according to national income level using information on the value-added share of farm products in U.S. food and beverage production (https://www.ers.usda.gov/data-products/food-dollar-series.aspx). This procedure resulted in relatively higher prices at higher income levels. Details are in the supplement (see supplementary information, technical appendix).

We divided each price series by an aggregate price level index for *food and nonalcoholic beverages* to adjust for differences in overall food prices across countries. This discounts any price differences across countries due to differences in overall food costs and implicitly accounts for the cross-price effects of food products not in the model.

The current analysis included 164 countries (4,264 stratum observations) having both GDD intake and ICP price data.

For national income, we used 2010 gross domestic product (GDP) data expressed in U.S. dollars per capita from the World Bank Development Indicators Database.(28) To account for differences in currency and purchasing power across economies, we used purchasing power parity (PPP) adjusted GDP. Since PPP-adjusted GDP accounts for inflationary factors across countries, we refer to our income measure as *real* per capita GDP. Income deciles were based on real per capita GDP for the 164 countries in the study.

Model and analysis

For peer review only - http://bmjopen.bmj.com/site/about/guidelines.xhtml

To estimate SSB intake demand, we applied a single-equation framework and used a semi-logarithmic functional form (see supplementary information, technical appendix).(29, 30) Many studies have used a double-log quadratic form.(31) However, a problem with the double-log form is that significant intake differences across subgroups can be lost in log conversions. A semi-log relationship allowed for a better assessment of subgroup effects on intake responsiveness. It has also been shown that semi-log models of demand are consistent with economic theory and contain the necessary information for obtaining, for instance, reliable measures of consumer welfare and the underlying preference structure of consumers.(29) Prior studies have also used a demand-system approach (multi-equation framework), primarily due to the need to account for the adding-up property when using expenditure data (i.e., expenditures on all consumption categories "add up" to total expenditures), which results in the error terms being correlated across categories. Since we are not estimating demand using an expenditure or allocation framework, the adopted approach is acceptable.

We accounted for age, sex, and regional differences by allowing these factors to have a direct effect on intake, as well as an additional effect through income and prices, including a quadratic age term to allow for nonlinear effects and the possibility of optimal responsiveness being between the youngest and oldest subgroups.

We accounted for varying preferences across countries due to factors not related to income or prices by including regional binary variables in the model: Southeast Asia, East Asia, and High Income Asia Pacific (Asia) (13 countries); Central Europe, Eastern Europe, and Central Asia (CEE/C. Asia) (27 countries); Latin America and the Caribbean (LAC) (30 countries); Middle East, North Africa, and South Asia (MENA/S. Asia) (23 countries); Sub-Saharan Africa (SSA) (45 countries); and High Income/Rest of World (HIC) (26 countries). HIC

For peer review only - http://bmjopen.bmj.com/site/about/guidelines.xhtml

was comprised largely of Western, industrialized countries; while not geographically connected, these countries share other similarities. We included several small island countries in this grouping because they were not sufficiently numerous to merit their own regional grouping (see supplemental table 2).

We utilized F-tests to compare a model including all explanatory variables and interaction terms to a series of restricted models and arrived at the final parsimonious model. Least-squares regression treats data independently and does not account for within-country correlations resulting in biased and comparatively small standard errors. Correcting for this, all models were estimated assuming country clusters, that is, independent errors across countries but correlated errors within countries, as well as heteroskedastic-consistent errors.(32) The elasticities reported in the following section were derived using the estimated coefficients from model 3 (final model) (see supplemental table 3).

Given WHO recommendations, we simulated how SSB intake would respond to a 20% tax (price increase).(19) Results were evaluated across countries by income decile for the following demographic subgroups: men and women, age < 35, 35-59, ≥ 60 years. We used probabilistic sensitivity analyses (Monte Carlo simulations) to derive 95% confidence intervals of intake responsiveness to the tax. Confidence intervals were based on the covariance matrix of the estimated coefficients, which accounted for the variability in the own-price relationship and the additional variability due to age, sex, and national income level.

Patient and public involvement

Patients and public were not involved.

Results

For peer review only - http://bmjopen.bmj.com/site/about/guidelines.xhtml

Global SSB intake

SSB intake levels varied significantly across countries (see supplemental figure 1) and by world region and age (figure 1). LAC had the highest median intake at 311 g/d (men) and 288 g/d (women) – almost four times the intake in SSA, and six times the lowest intake region (Asia). Across age/sex strata globally, the group with the highest median intake was young men, age 20 (209 g/d), followed closely by young women, age 20 (188 g/d). Compared to 20-year olds, median global intake in men and women, age 80, was about 75% lower. Across age and sex strata worldwide, the highest intake level was observed for men, age 20, in Trinidad and Tobago (1,239 g/d), and the lowest intake for women, age 80, in China (6 g/d). A more detailed discussion of global SSB intake by age, sex, and world region is available.(33)

SSB own-price elasticities

Given the variables in the final model, it was more appropriate to derive elasticities across country groups based on income level. We derived and compared SSB own-price elasticities across all strata jointly by age, sex, and global income decile (figure 2 and table 1; also see supplemental table 4). Note that reported values are derived at median intake levels by age and sex subgroup. Thus, observed differences across age, sex, and income decile are solely a function of own-price interactions with sex, age, and income. At any given age, SSB intake became less responsive to price changes with rising income. For instance, in women, age 20, the own-price elasticities ranged from -0.90 ($p<0.001$) for the lowest income decile to -0.47 ($p<0.001$) for the highest income decile. The decline in responsiveness became more pronounced with age. For instance, in men, age 80, the own-price elasticities ranged from -1.91 ($p<0.001$) for the lowest income decile to -0.43 ($p>0.100$) for the highest income decile. The

For peer review only - http://bmjopen.bmj.com/site/about/guidelines.xhtml

influence of age on SSB own-price elasticities varied depending on income status. At lower income levels, elasticities were strongest (became more negative) at older ages; but at middle and higher income levels, there was less influence of age on elasticities. The least responsive group were middle-aged adults, particularly in upper-middle and higher income deciles.

Potential impact of SSB taxes on intake

Potential reductions in median intake from a 20% tax (price increase) were largest for the lowest income decile, ranging from 14.5% (95% CI: -0.4, 29.5) to 24.1% (5.3, 44.4), depending on age and sex (table 2). Across income deciles, reductions varied less in younger adults (age < 35) – for example, ranging from 16.8% (8.6, 25.0) in young men in the lowest income decile to 7.9% (2.2, 13.6) in the highest income decile – than in older adults (e.g., men, age ≥ 60). This is consistent with the much higher baseline SSB intakes among younger adults globally (figure 1), suggesting that such intake will be significantly influenced by taxes regardless of income status. Older men and women (age ≥ 60) in the lowest income decile were estimated to be most influenced by SSB taxes, suggesting a high price-responsiveness to such a luxury in poor nations globally. Insignificant outcomes were mostly observed for middle-aged and older adults in middle and higher income deciles.

Discussion

In this global analysis of SSB intakes and prices, we identified significant price responsiveness in nearly every age, sex, and country income subgroup worldwide. We also identified significant heterogeneity in these potential responses. Price responsiveness was higher in lower income than in wealthier countries, consistent with expectations and the much higher

For peer review only - http://bmjopen.bmj.com/site/about/guidelines.xhtml

relative share of income spent on food and other necessities in low-income countries. Interestingly, the response by age varied by national income. In lower income countries, own-price responsiveness increased with age, but less so in middle and higher income countries.

Finally, our estimates of effects of a 20% tax suggested significant SSB intake reductions across income levels, particularly for young adults. Outcomes for middle-aged adults, and older adults at higher income levels, were not significant.

Strengths and limitations

This study has several strengths, the first being the extensive country coverage. We provide a global snapshot of SSB intake behavior allowing for comparisons within and across most countries. Since past studies have been limited to a single country or a select group of countries, the results of this study inform policy and decision-making beyond the current state of knowledge. Problems associated with poor diets and NCDs occur in both developing and developed countries.(34) A comparative analysis across the complete spectrum of countries can assist international organizations in developing heterogeneous strategies for specific subgroups and countries. Our use of individual intakes by age, sex, and country provides for more accurate representation of dietary behavior. Previous findings based on expenditure data may be limited by differences in expenditures and actual consumption.

Potential limitations should also be considered. First, being a modeling study, the projected outcomes can only inform how taxes could affect behavior. While an intervention study would be more fitting, interventions across 164 countries would not be feasible. Secondly, our analysis was limited by the use of price and income data at the national level. Ideally, our explanatory variables would also be at the subgroup level, reflecting that incomes typically vary

For peer review only - http://bmjopen.bmj.com/site/about/guidelines.xhtml

For peer review only

with age and sex, and different subgroups could face a different set of prices within a country. For instance, in countries where urban populations are relatively young, young adults could face different prices depending on market conditions in urban and rural areas. This limitation is due to the number of countries in our study. Such detailed data is not available for many countries.

While it would be ideal to have a time series of global SSB intake data, unfortunately these data do not exist. However, there is value in examining data at a point-in-time and intake in one demographic group compared to other groups, as well as comparing intake patterns across countries. Our purpose is to inform how demographic subgroups across countries might respond to price signals in form of taxes. There is value in understanding the relative responsiveness which can be gleaned from a cross-country snapshot.

The use of the global sugar prices as a proxy for SSB prices raises questions about the primary relationship of interest (SSB own-price elasticity). For higher income countries where farm production costs are a small share of the final product price, the proxy is less suitable and could result is lower “own-price” responsiveness. Accordingly, we adjusted the price index to account for higher SSB prices relative to sugar prices at higher income levels. The adjustment resulted in a 10- to 15-fold increase in the index value for higher income countries similar to the U.S. For low-income countries, adjusted and unadjusted prices were not that dissimilar (see supplemental figure 2). Using adjusted prices, we found significantly higher own-price responsiveness compared to estimates using unadjusted prices.

Comparison with other studies

Since previous research has mostly focused on higher income countries, primarily the U.S., it is difficult to compare all of our results with earlier findings. Several U.S. based studies

For peer review only - http://bmjopen.bmj.com/site/about/guidelines.xhtml

For peer review only

have considered how SSB consumption would respond to a tax. Given a 10% tax, the projected decrease in SSB sales ranged from 6.7% to 18.2%.(15) These results are greater than our findings for middle-aged and older adults in the highest income decile, but are closer to our findings for young adults (7.3%, women, age < 35, and 7.9%, men, age < 35), albeit we are considering a 20% tax.

Our tax outcomes are due to comparably smaller own-price elasticities. Whereas our own-price elasticity estimates for the highest income countries range from -0.5 to -0.0, meta-analyses of U.S. studies give estimates of -0.8 (-3.2 to -0.13) and -1.1 (-1.3 to -0.9).(16, 35) In a study of Mexico using data before and after implementation of a national soda tax (10%) in 2014, SSB purchases decreased by an average of 6% during the first year of implementation,(12) which is actually comparable to our findings for young adults in middle-income countries. Other studies of Latin American countries using household survey data reported estimates more comparable to our results for lower income countries.(36-38)

The fact that our estimates are relative smaller does not necessarily make them less accurate. Note that past studies have mostly used expenditure data. It has been documented that significant changes in expenditures do not always result in changes in the quantity or quality of food consumption.(39) In fact, studies have found the association between food expenditures and intake to be particularly weak and insufficient for diet and nutrition research.(40) For instance, a recent study of the SSB tax in Berkeley, California, U.S. found significant reductions in consumer spending on SSBs, increased spending on substitute beverages, but insignificant reductions in reported SSB intake.(41) Another issue is that SSBs are less perishable that other foods. When goods have an extended shelf life, individuals can take advantage of price

For peer review only - http://bmjopen.bmj.com/site/about/guidelines.xhtml

discounts, increasing expenditures when prices are low, stock piling for future consumption. Ignoring this fact can result in overestimates of own-price elasticities.(42)

Conclusion

This is the first study to examine SSB consumption and taxation in a global context. Our findings provide a better understanding of the potential effectiveness of taxes across the full spectrum of countries. Overall, we found that the influence of SSB prices on intake significantly depends on the income status of countries. Our results suggest that intake reductions (in percent) could be small or negligible for certain demographics in higher income countries. Although small in percentage terms, actual intake reductions could still be sizeable enough for high-consuming subgroups for taxes to be worth pursuing. For higher income countries, a larger tax or a tax combined with other approaches might be needed to significantly change behavior. For instance, taxes could be combined with media and education campaigns, food labeling, and other interventions.(43) For all adults in lower income countries and young adults globally, our findings indicate that taxes would be particularly effective, which is to be expected since food expenditures account for a greater share of income for these groups making them more sensitive to prices.

For peer review only

References

1. de Koning L, Malik VS, Kellogg MD, Rimm EB, Willett WC, Hu FB. Sweetened beverage consumption, incident coronary heart disease, and biomarkers of risk in men. Circulation. 2012;125(14):1735-41.

2. Fung TT, Malik V, Rexrode KM, Manson JE, Willett WC, Hu FB. Sweetened beverage consumption and risk of coronary heart disease in women. The American Journal of Clinical Nutrition. 2009;89(4):1037-42.

3. Ludwig DS, Peterson KE, Gortmaker SL. Relation between consumption of sugar-sweetened drinks and childhood obesity: a prospective, observational analysis. Lancet. 2001;357(9255):505-8.

4. Malik VS, Popkin BM, Bray GA, Després J-P, Willett WC, Hu FB. Sugar-sweetened beverages and risk of metabolic syndrome and type 2 diabetes. Diabetes Care. 2010;33(11):2477-83.

5. Mozaffarian D, Hao T, Rimm EB, Willett WC, Hu FB. Changes in diet and lifestyle and long-term weight gain in women and men. N Engl J Med. 2011;364(25):2392-404.

6. Eyles H, Ni Mhurchu C, Nghiem N, Blakely T. Food pricing strategies, population diets, and non-communicable disease: a systematic review of simulation studies. PLoS Med. 2012;9(12):e1001353.

7. World Health Organization. Using Price Policies to Promote Healthier Diets. Copenhagen, Denmark: WHO Regional Office for Europe; 2015.

8. Gustin G. Latest Soda Tax Puts Pressure on Waistlines and the World. National Geographic. 2016 March 17, 2016.

9. Grogger J. Soda Taxes and the Prices of Sodas and Other Drinks: Evidence from Mexico. National Bureau of Economic Research; 2015. Contract No.: NBER Working Paper No. 21197.

10. Paarlberg R, Mozaffarian D, Micha R. Viewpoint: Can U.S. local soda taxes continue to spread? Food Pol. 2017;71:1-7.

11. Backholer K, Vandevijvere S, Blake M, Tseng M. Sugar-sweetened beverage taxes in 2018: a year of reflections and consolidation. Public Health Nutr. 2018;21(18):3291-5.

12. Colchero MA, Popkin BM, Rivera JA, Ng SW. Beverage Purchases from Stores in Mexico Under the Excise Tax on Sugar Sweetened Beverages: Observational Study. BMJ. 2016;352:h6704.

13. Brownell KD, Farley T, Willett WC, Popkin BM, Chaloupka FJ, Thompson JW, et al. The public health and economic benefits of taxing sugar-sweetened beverages. N Engl J Med. 2009;361(16):1599-605.

For peer review only - http://bmjopen.bmj.com/site/about/guidelines.xhtml

14. Nakhimovsky SS, Feigl AB, Avila C, O'Sullivan G, Macgregor-Skinner E, Spranca M. Taxes on sugar-sweetened beverages to reduce overweight and obesity in middle-income countries: a systematic review. PLoS One. 2016;11(9):e0163358.

15. Afshin A, Peñalvo JL, Del Gobbo L, Silva J, Michaelson M, O'Flaherty M, et al. The prospective impact of food pricing on improving dietary consumption: A systematic review and meta-analysis. PLoS One. 2017;12(3):e0172277.

16. Powell LM, Chriqui JF, Khan T, Wada R, Chaloupka FJ. Assessing the potential effectiveness of food and beverage taxes and subsidies for improving public health: a systematic review of prices, demand and body weight outcomes. Obes Rev. 2013;14(2):110-28.

17. Briggs ADM, Mytton OT, Kehlbacher A, Tiffin R, Rayner M, Scarborough P. Overall and income specific effect on prevalence of overweight and obesity of 20% sugar sweetened drink tax in UK: econometric and comparative risk assessment modelling study. BMJ. 2013;347:f6189.

18. Grogger J. Soda taxes and the prices of sodas and other drinks: evidence from Mexico. Amer J Agr Econ. 2017;99(2):481-98.

19. World Health Organization. Fiscal Policies for Diet and the Prevention of Noncommunicable Diseases. Technical Meeting Report. Geneva, Switzerland: World Health Organization; 2016.

20. Mytton OT, Clarke D, Rayner M. Taxing unhealthy food and drinks to improve health. BMJ. 2012;344(7857):e2931.

21. Del Gobbo LC, Khatibzadeh S, Imamura F, Micha R, Shi P, Smith M, et al. Assessing global dietary habits: a comparison of national estimates from the FAO and the Global Dietary Database. Am J Clin Nutr. 2015;101(5):1038-46.

22. Imamura F, Micha R, Khatibzadeh S, Fahimi S, Shi P, Powles J, et al. Dietary quality among men and women in 187 countries in 1990 and 2010: a systematic assessment. Lancet Global Health. 2015;3(3):e132-e42.

23. Micha R, Kalantarian S, Wirojratana P, Byers T, Danaei G, Elmadfa I, et al. Estimating the global and regional burden of suboptimal nutrition on chronic disease: methods and inputs to the analysis. Eur J Clin Nutr. 2012;66(1):119-29.

24. Micha R, Khatibzadeh S, Shi P, Fahimi S, Lim S, Andrews KG, et al. Global, regional, and national consumption levels of dietary fats and oils in 1990 and 2010: a systematic analysis including 266 country-specific nutrition surveys. BMJ. 2014;348:g2272.

25. Khatibzadeh S, Kashaf MS, Micha R, Fahimi S, Shi P, Elmadfa I, et al. A global database of food and nutrient consumption. Bull World Health Organ. 2016;94(12):931-4E.

26. World Bank. Purchasing Power Parities and Real Expenditures of World Economies Washington, D.C.: World Bank Publications; 2014.

For peer review only - http://bmjopen.bmj.com/site/about/guidelines.xhtml

27. World Bank. Purchasing Power Parities and the Real Size of World Economies Washington, D.C.: World Bank Publications; 2015.

28. World Bank. World Development Indicators 2015 [Available from: http://data.worldbank.org/data-catalog/world-development-indicators.

29. LaFrance JT. Incomplete demand systems and semilogarithmic demand models. Australian J Agr Econ. 1990;34(2):118-31.

30. von Haefen RH. A complete characterization of the linear, log-linear, and semi-log incomplete demand system models. J Agr Resource Econ. 2002;27(2):281-319.

31. Sahn DE. The Effect of Price and Income Changes on Food-Energy Intake in Sri Lanka. Econ Devel Cult Change. 1988;36(2):315-40.

32. Cameron AC, Trivedi PK. Microeconometrics: Methods and Applications. Cambridge: Cambridge University Press; 2005.

33. Singh GM, Micha R, Khatibzadeh S, Shi P, Lim S, Andrews KG, et al. Global, regional, and national consumption of sugar-sweetened beverages, fruit juices, and milk: a systematic assessment of beverage intake in 187 countries. PLoS One. 2015;10(8):e0124845.

34. United Nations. Note by the Secretary-General transmitting the report of the Director-General of the World Health Organization on the prevention and control of non-communicable diseases. New York: United Nations; 2013. Report No.: A/68/650 Contract No.: A/68/650.

35. Andreyeva T, Long MW, Brownell KD. The impact of food prices on consumption: a systematic review of research on the price elasticity of demand for food. Am J Public Health. 2010;100(2):216-22.

36. Colchero MA, Salgado JC, Unar-Munguía M, Hernández-Ávila M, Rivera-Dommarco JA. Price elasticity of the demand for sugar sweetened beverages and soft drinks in Mexico. Econ Hum Biol. 2015;19:129-37.

37. Guerrero-López CM, Unar-Munguía M, Colchero MA. Price elasticity of the demand for soft drinks, other sugar-sweetened beverages and energy dense food in Chile. BMC Public Health. 2017;17(1):180.

38. Paraje G. The Effect of Price and Socio-Economic Level on the Consumption of Sugar-Sweetened Beverages (SSB): The Case of Ecuador. PLoS One. 2016;11(3):e0152260.

39. Mark Aguiar, Erik Hurst. Consumption versus Expenditure. J Polit Economy. 2005;113(5):919-48.

40. Monsivais P, Perrigue MM, Adams SL, Drewnowski A. Measuring diet cost at the individual level: a comparison of three methods. Eur J Clin Nutr. 2013;67:1220.

For peer review only - http://bmjopen.bmj.com/site/about/guidelines.xhtml

41. Silver LD, Ng SW, Ryan-Ibarra S, Taillie LS, Induni M, Miles DR, et al. Changes in prices, sales, consumer spending, and beverage consumption one year after a tax on sugar-sweetened beverages in Berkeley, California, US: A before-and-after study. PLoS Med. 2017;14(4):e1002283.

42. Hartmann WR. Intertemporal effects of consumption and their implications for demand elasticity estimates. Quantitative Marketing and Economics. 2006;4(4):325-49.

43. Afshin A, Micha R, Khatibzadeh S, Schmidt LA, Mozaffarian D. Dietary Policies to Reduce Noncommunicable Diseases. In: Brown GW, Yamey G, Wamala S, editors. The Handbook of Global Health Policy. West Sussex, UK: Wiley-Blackwell; 2014. p. 175-93.

For peer review only

For peer review only - http://bmjopen.bmj.com/site/about/guidelines.xhtml

Figure 1 Comparison of mean SSB intakes among adults in age, sex, and country-specific strata across world regions and globally by select age groups. *n* represents the number of age, sex, and country-specific subgroups in each stratum. Boxes represent the median intake Value and interquartile range; error bars represent the minimum and maximum values. Source: Global Dietary Database, 2010

Figure 2 Global SSB own-price elasticities by age, sex, and global income decile. Values are derived at median intake levels by demographic subgroup. Own-price elasticities are based on 1% price changes. Income deciles are based on the national income of the 164 countries included in the study. Each decile is comprised of 16 countries (except the 4 lowest deciles, which are each comprised of 17 countries). The per capita income range (PPP-adjusted in thousand $US) for each decile: (1st) $0.6-$1.5, (2nd) $1.5-$2.7, (3rd) $2.7-$5.3, (4th) $5.5-$8.0, (5th) $8.3-$10.8, (6th) $11.1-$15.2, (7th) $15.3-$20.3, (8th) $20.6-$29.4, (9th) $30.4-$40.9, and (10th) $41.3-$127.2.

For peer review only

For peer review only - http://bmjopen.bmj.com/site/about/guidelines.xhtml

Table 1 Own-price elasticities of SSB intake by age, sex, and global income decile†

Income decile‡	Age 20	Age 30	Age 40	Age 50	Age 60	Age 70	Age 80	Population-weighted average
	Women							
Lowest 10%	-0.90 (0.21)***	-0.80 (0.25)***	-0.78 (0.35)**	-0.70 (0.42)*	-0.78 (0.49)	-1.11 (0.53)**	-1.84 (0.60)***	-0.82 (0.30)***
2nd	-0.83 (0.18)***	-0.71 (0.21)***	-0.65 (0.29)**	-0.54 (0.34)	-0.58 (0.38)	-0.88 (0.41)**	-1.59 (0.46)***	-0.71 (0.25)***
3rd	-0.76 (0.16)***	-0.62 (0.18)***	-0.51 (0.23)**	-0.36 (0.27)	-0.37 (0.29)	-0.65 (0.30)**	-1.33 (0.34)***	-0.59 (0.21)***
4th	-0.70 (0.14)***	-0.54 (0.16)***	-0.40 (0.20)**	-0.22 (0.22)	-0.19 (0.22)	-0.45 (0.21)**	-1.10 (0.24)***	-0.49 (0.17)***
5th	-0.67 (0.14)***	-0.49 (0.15)***	-0.32 (0.18)*	-0.12 (0.19)	-0.07 (0.19)	-0.32 (0.17)*	-0.96 (0.18)***	-0.40 (0.16)**
6th	-0.64 (0.13)***	-0.45 (0.14)***	-0.26 (0.17)	-0.04 (0.18)	0.02 (0.18)	-0.21 (0.15)	-0.84 (0.16)***	-0.33 (0.15)**
7th	-0.60 (0.13)***	-0.41 (0.14)***	-0.20 (0.17)	0.04 (0.19)	0.11 (0.18)	-0.11 (0.15)	-0.72 (0.16)***	-0.27 (0.11)**
8th	-0.57 (0.13)***	-0.36 (0.14)**	-0.13 (0.18)	0.13 (0.20)	0.23 (0.20)	0.02 (0.18)	-0.58 (0.19)***	-0.22 (0.11)**
9th	-0.53 (0.14)***	-0.31 (0.15)**	-0.05 (0.20)	0.23 (0.22)	0.35 (0.24)	0.16 (0.23)	-0.43 (0.24)*	-0.15 (0.06)**
Highest 10%	-0.47 (0.15)***	-0.23 (0.17)	0.06 (0.23)	0.37 (0.27)	0.52 (0.30)	0.35 (0.31)	-0.22 (0.34)	-0.11 (0.07)
	Men							
Lowest 10%	-0.87 (0.19)***	-0.79 (0.23)***	-0.83 (0.32)***	-0.81 (0.39)**	-0.91 (0.45)**	-1.24 (0.50)**	-1.91 (0.55)***	-0.84 (0.30)***
2nd	-0.81 (0.17)***	-0.71 (0.19)***	-0.71 (0.27)***	-0.66 (0.32)**	-0.73 (0.36)**	-1.03 (0.39)***	-1.68 (0.43)***	-0.76 (0.23)***
3rd	-0.75 (0.15)***	-0.63 (0.16)***	-0.59 (0.22)***	-0.50 (0.25)**	-0.53 (0.27)*	-0.81 (0.28)***	-1.44 (0.32)***	-0.59 (0.21)***
4th	-0.69 (0.13)***	-0.56 (0.14)***	-0.48 (0.18)***	-0.36 (0.20)*	-0.36 (0.21)*	-0.62 (0.20)***	-1.24 (0.23)***	-0.54 (0.16)***
5th	-0.66 (0.13)***	-0.51 (0.13)***	-0.41 (0.17)**	-0.27 (0.18)	-0.25 (0.18)	-0.50 (0.16)***	-1.10 (0.19)***	-0.40 (0.16)**
6th	-0.63 (0.12)***	-0.48 (0.13)***	-0.35 (0.16)**	-0.19 (0.17)	-0.17 (0.17)	-0.40 (0.14)***	-0.99 (0.17)***	-0.42 (0.14)***
7th	-0.60 (0.12)***	-0.44 (0.13)***	-0.30 (0.16)*	-0.12 (0.17)	-0.08 (0.17)	-0.31 (0.14)**	-0.89 (0.16)***	-0.28 (0.13)**
8th	-0.57 (0.12)***	-0.40 (0.13)***	-0.23 (0.17)	-0.04 (0.18)	0.02 (0.18)	-0.19 (0.17)	-0.76 (0.19)***	-0.29 (0.12)**
9th	-0.53 (0.13)***	-0.35 (0.14)**	-0.16 (0.18)	0.06 (0.21)	0.14 (0.22)	-0.06 (0.21)	-0.62 (0.23)***	-0.16 (0.09)*
Highest 10%	-0.49 (0.14)***	-0.28 (0.15)*	-0.06 (0.21)	0.19 (0.25)	0.29 (0.28)	0.11 (0.29)	-0.43 (0.31)	-0.16 (0.10)

Values are derived at median intake levels by demographic subgroup. Standard errors are in (parenthesis). Population weights by sex, age, and income status were obtained from the World Development Indicators Data Bank: https://databank.worldbank.org/data/reports.aspx?source=world-development-indicators#.
*p≤0.10; **p≤0.05; ***p≤0.01.
†Price elasticities are based on 1% price changes. For instance, given a 1% SSB price increase in the lowest income countries, intake by women, age 20 falls by 0.90%. ‡Income deciles are based on the national income of the 164 countries included in the study. Each decile is comprised of 16 countries (except the 4 lowest deciles, which are each comprised of 17 countries). The per capita income range (PPP-adjusted in thousand $US) for each decile: (1st) $0.6-$1.5, (2nd) $1.5-$2.7, (3rd) $2.7-$5.3, (4th) $5.5-$8.0, (5th) $8.3-$10.8, (6th) $11.1-$15.2, (7th) $15.3-$20.3, (8th) $20.6-$29.4, (9th) $30.4-$40.9, and (10th) $41.3-$127.2.

For peer review only

Table 2 Potential impact of a 20% tax (price increase) on SSB intake by age, sex, and global income decile.

Income decile†	Women age < 35	Men age < 35	Women 35 ≤ age < 60	Men 35 ≤ age < 60	Women age ≥ 60	Men age ≥ 60
	Percentage change in intake (95% CI)					
Lowest 10%	-17.1 (-26.1 to -8.1)	-16.8 (-25.0 to -8.6)	-14.5 (-29.5 to 0.4)	-15.9 (-29.5 to -2.2)	-22.3 (-43.2 to -1.4)	-24.9 (-44.4 to -5.3)
2nd	-15.6 (-23.3 to -7.9)	-15.4 (-22.5 to -8.3)	-11.6 (-23.8 to 0.7)	-13.2 (-24.3 to -1.9)	-17.7 (-33.9 to -1.4)	-20.6 (-35.9 to -5.3)
3rd	-14.0 (-20.6 to -7.4)	-14.0 (-20.1 to -7.8)	-8.5 (-18.3 to 1.3)	-10.4 (-19.4 to -1.4)	-13.0 (-24.8 to -1.2)	-16.3 (-27.4 to -5.1)
4th	-12.6 (-18.5 to -6.7)	-12.7 (-18.2 to -7.3)	-5.9 (-14.0 to 2.2)	-8.0 (-15.5 to -0.5)	-9.0 (-17.3 to -0.7)	-12.5 (-20.5 to -4.6)
5th	-11.7 (-17.3 to -6.2)	-11.9 (-17.1 to -6.7)	-4.2 (-11.6 to 3.2)	-6.5 (-13.2 to 0.3)	-6.3 (-12.9 to 0.3)	-10.1 (-16.4 to -3.7)
6th	-11.0 (-16.4 to -5.6)	-11.3 (-16.3 to -6.2)	-2.8 (-9.9 to 4.3)	-5.2 (-11.6 to 1.3)	-4.2 (-10 to 1.7)	-8.1 (-13.7 to -2.4)
7th	-10.3 (-15.7 to -5.0)	-10.6 (-15.6 to -5.7)	-1.4 (-8.5 to 5.6)	-4.0 (-10.4 to 2.5)	-2.1 (-8 to 3.9)	-6.2 (-11.8 to -0.4)
8th	-9.5 (-14.9 to -4.1)	-9.9 (-14.9 to -4.9)	0.2 (-7.3 to 7.6)	-2.5 (-9.2 to 4.3)	0.4 (-6.6 to 7.5)	-3.8 (-10.4 to 2.8)
9th	-8.5 (-14.2 to -2.9)	-9.0 (-14.3 to -3.8)	2.0 (-6.3 to 10.3)	-0.8 (-8.3 to 6.7)	3.2 (-5.8 to 12.3)	-1.2 (-9.6 to 7.2)
Highest 10%	-7.3 (-13.5 to -1.1)	-7.9 (-13.6 to -2.2)	4.5 (-5.4 to 14.3)	1.4 (-7.6 to 10.3)	7.0 (-5.3 to 19.3)	2.2 (-9.1 to 13.6)

Values are reductions from median intake levels for each demographic subgroup.
†Income deciles are based on the national income of the 164 countries included in the study. Each decile is comprised of 16 countries except the 4 lowest deciles, which are each comprised of 17 countries. The per capita income range (PPP-adjusted in thousand $US) for each decile: (1st) $0.6-$1.5, (2nd) $1.5-$2.7, (3rd) $2.7-$5.3, (4th) $5.5-$8.0, (5th) $8.3-$10.8, (6th) $11.1-$15.2, (7th) $15.3-$20.3, (8th) $20.6-$29.4, (9th) $30.4-$40.9, and (10th) $41.3-$127.2.

For peer review only

<Insert Figure1.png file here>

Figure 1 Comparison of mean SSB intakes among adults in age, sex, and country-specific strata across world regions and globally by select age groups. *n* represents the number of age, sex, and country-specific subgroups in each stratum. Boxes represent the median intake value and interquartile range; error bars represent the minimum and maximum values. Source: Global Dietary Database, 2010

For peer review only

For peer review only - http://bmjopen.bmj.com/site/about/guidelines.xhtml

<Insert Figure2.png file here>

Figure 2 Global SSB own-price elasticities by age, sex, and global income decile. Values are derived at median intake levels by demographic subgroup. Own-price elasticities are based on 1% price changes. Income deciles are based on the national income of the 164 countries included in the study. Each decile is comprised of 16 countries (except the 4 lowest deciles, which are each comprised of 17 countries). The per capita income range (PPP-adjusted in thousand $US) for each decile: (1st) $0.6-$1.5, (2nd) $1.5-$2.7, (3rd) $2.7-$5.3, (4th) $5.5-$8.0, (5th) $8.3-$10.8, (6th) $11.1-$15.2, (7th) $15.3-$20.3, (8th) $20.6-$29.4, (9th) $30.4-$40.9, and (10th) $41.3-$127.2.

For peer review only

For peer review only - http://bmjopen.bmj.com/site/about/guidelines.xhtml

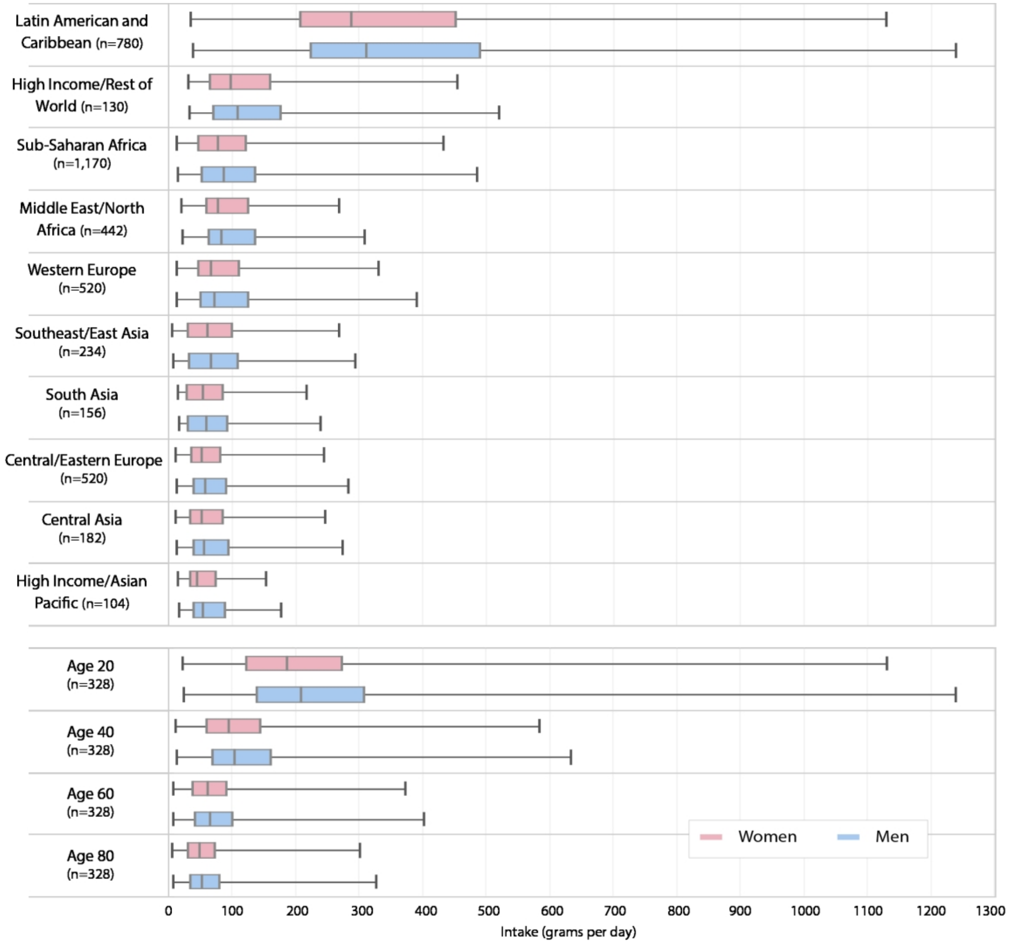

Figure 1 Comparison of mean SSB intakes among adults in age, sex, and country-specific strata across world regions and globally by select age groups. n represents the number of age, sex, and country-specific subgroups in each stratum. Boxes represent the median intake value and interquartile range; error bars represent the minimum and maximum values.
Source: Global Dietary Database, 2010

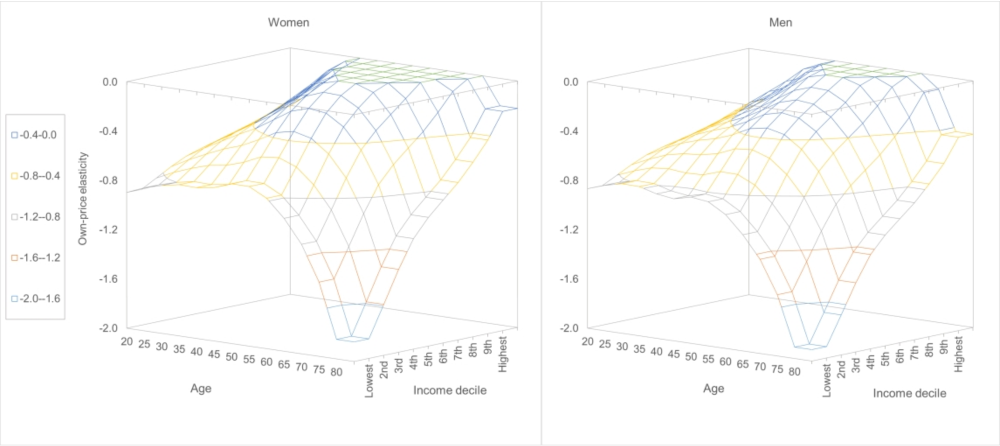

Figure 2 Global SSB own-price elasticities by age, sex, and global income decile. Values are derived at median intake levels by demographic subgroup. Own-price elasticities are based on 1% price changes. Income deciles are based on the national income of the 164 countries included in the study. Each decile is comprised of 16 countries (except the 4 lowest deciles, which are each comprised of 17 countries). The per capita income range (PPP-adjusted in thousand $US) for each decile: (1st) $0.6-$1.5, (2nd) $1.5-$2.7, (3rd) $2.7-$5.3, (4th) $5.5-$8.0, (5th) $8.3-$10.8, (6th) $11.1-$15.2, (7th) $15.3-$20.3, (8th) $20.6-$29.4, (9th) $30.4-$40.9, and (10th) $41.3-$127.2.

Supplementary Information

Global patterns in price elasticities of sugar-sweetened beverage intake and potential effectiveness of tax policy: a cross-sectional study of 164 countries by sex, age, and global-income decile

Andrew Muhammad,[1] Birgit Meade,[2] David R Marquardt,[2] and Dariush Mozaffarian[3]

[1]University of Tennessee Institute of Agriculture, Department of Agricultural and Resource Economics, Knoxville, Tennessee, USA
[2]United States Department of Agriculture, Economic Research Service, Market and Trade Economics Division, Washington, D.C., USA
[3]Tufts University, Friedman School of Nutrition Science and Policy, Boston, Massachusetts, USA

Supplemental Figure 1	Mean SSB intake across countries in adults, age ≥ 20
Supplemental Table 1	Description of ICP food-price categories
Supplemental Table 2	Countries included in study by region (aggregate regions used for estimation)
Supplemental Table 3	Demand model estimates for SSB intake
Supplemental Table 4	Own-price elasticities of SSB intake by age, sex, and global income decile
Technical Appendix	Demand model and methods
Supplemental Figure 2	Deflated sugar price index: unadjusted and adjusted

For peer review only - http://bmjopen.bmj.com/site/about/guidelines.xhtml

Supplementary Information

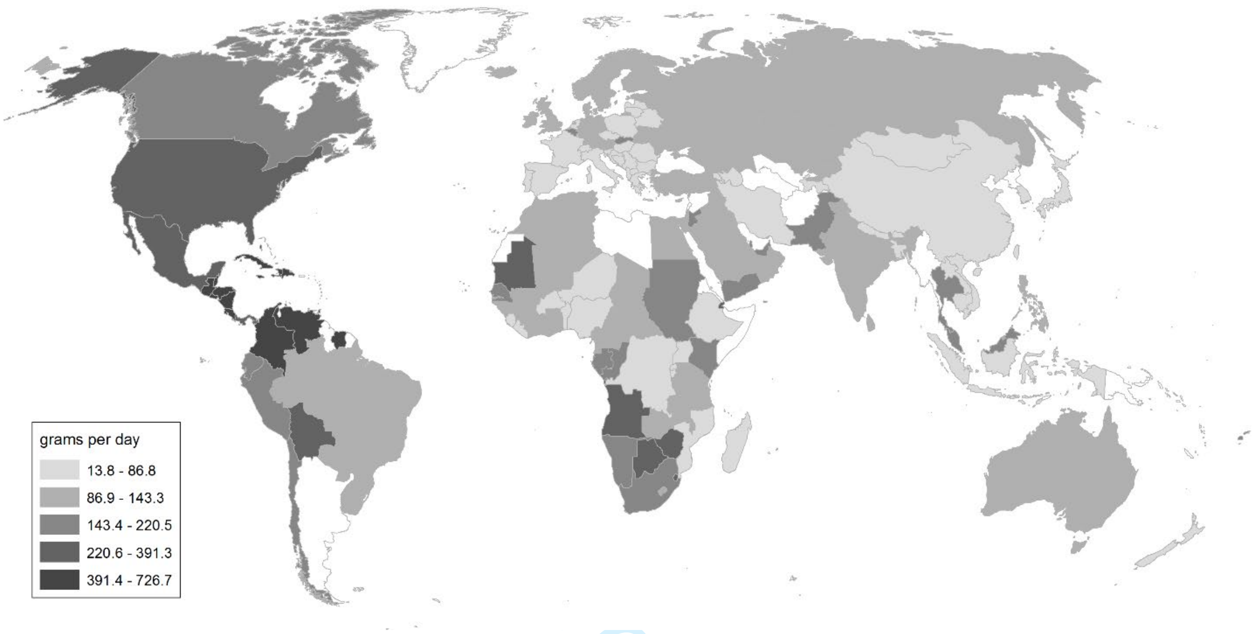

Supplemental Figure 1

Mean SSB intake across countries in adults, age ≥ 20

Map was created using ArcGIS® software by Esri. ArcGIS® and ArcMap™ are the intellectual property of Esri and are used herein under license. Copyright © Esri. All rights reserved. For more information about Esri® software, please visit www.esri.com.

Source: Global Dietary Database, 2010

For peer review only - http://bmjopen.bmj.com/site/about/guidelines.xhtml

Supplemental Table 1

Description of ICP food-price categories

ICP food-price category
• *Fresh or chilled fruit* – All fresh or chilled fruit including melons and water melons; excludes vegetables grown for their fruit such as cucumbers and tomatoes.
• *Fresh milk* – Raw milk; pasteurised or sterilised milk; includes whole and low fat milk; recombined or reconstituted milk; soya milk.
• *Sugar* – Cane or beet sugar, unrefined or refined, powdered, crystallised or in lumps; includes artificial sugar substitutes.

For peer review only

For peer review only - http://bmjopen.bmj.com/site/about/guidelines.xhtml

Supplemental Table 2

Countries included in study by region (aggregate regions used for estimation)

Region	Countries
Southeast Asia, East Asia, and High Income Asia Pacific (Asia) (13 countries)	Brunei Darussalam, Cambodia, China, Indonesia, Japan, South Korea, Laos, Malaysia, Maldives, Philippines, Singapore, Thailand, and Vietnam.
Central Europe, Eastern Europe, and Central Asia (CEE/C. Asia) (27 countries)	Albania, Armenia, Azerbaijan, Belarus, Bosnia and Herzegovina, Bulgaria, Croatia, Czech Republic, Estonia, Georgia, Hungary, Kazakhstan, Kyrgyz Republic, Latvia, Lithuania, Macedonia, Moldova, Mongolia, Montenegro, Poland, Romania, Russia, Serbia, Slovak Republic, Slovenia, Tajikistan, and Ukraine.
Latin America and the Caribbean (LAC) (30 countries)	Antigua and Barbuda, Bahamas, Barbados, Belize, Bolivia, Brazil, Chile, Colombia, Costa Rica, Cuba, Dominica, Dominican Republic, Ecuador, El Salvador, Grenada, Guatemala, Haiti, Honduras, Jamaica, Mexico, Nicaragua, Panama, Paraguay, Peru, St. Lucia, St. Vincent and Grenadines, Suriname, Trinidad and Tobago, Uruguay, and Venezuela.
Middle East, North Africa, and South Asia (MENA/S. Asia) (23 countries)	Algeria, Bahrain, Bangladesh, Bhutan, Egypt, India, Iran, Iraq, Israel, Jordan, Kuwait, Morocco, Nepal, Oman, Pakistan, Qatar, Saudi Arabia, Sri Lanka, Tunisia, Turkey, United Arab Emirates, West Bank and Gaza, and Yemen.
Sub-Saharan Africa (SSA) (45 countries)	Angola, Benin, Botswana, Burkina Faso, Burundi, Cameroon, Cape Verde, Central African Republic, Chad, Comoros, Congo, Côte d'Ivoire, Djibouti, DR Congo, Equatorial Guinea, Ethiopia, Gabon, Gambia, Ghana, Guinea, Guinea-Bissau, Kenya, Lesotho, Liberia, Madagascar, Malawi, Mali, Mauritania, Mauritius, Mozambique, Namibia, Niger, Nigeria, Rwanda, São Tomé and Principe, Senegal, Sierra Leone, South Africa, Sudan, Swaziland, Tanzania, Togo, Uganda, Zambia, and Zimbabwe.
High Income/Rest of World (HIC) (26 countries)	Australia, Austria, Belgium, Canada, Cyprus, Denmark, Fiji, Finland, France, Germany, Greece, Iceland, Ireland, Italy, Luxembourg, Malta, Netherlands, New Zealand, Norway, Portugal, Seychelles, Spain, Sweden, Switzerland, United Kingdom, and United States.

For peer review only

For peer review only - http://bmjopen.bmj.com/site/about/guidelines.xhtml

Supplemental Table 3

Demand model estimates for SSB intake

Variable	model 1 estimate (SE)	model 2 estimate (SE)	model 3 (final model) estimate (SE)
constant	436.63 (25.47)***	-784.78 (378.46)**	-1,398.66 (535.82)***
female (F)	-13.36 (0.82)***	-13.36 (0.82)***	28.17 (15.92)*
age	-10.87 (0.74)***	-10.87 (0.74)***	14.06 (11.51)
age^2	0.08 (0.01)***	0.08 (0.01)***	-0.15 (0.09)*
SSA	1.83 (11.47)	35.51 (17.22)**	78.19 (37.85)**
LAC	258.41 (26.38)***	251.12 (28.01)***	546.89 (62.00)***
MENA/S. Asia	-10.05 (10.57)	7.45 (13.56)	21.20 (28.71)
CEE/C. Asia	-32.87 (9.99)***	-18.44 (12.59)	-28.38 (26.74)
Asia	-26.85 (14.66)*	-59.53 (19.50)***	-121.47 (41.21)***
age × SSA			-0.85 (0.42)**
age × LAC			-5.92 (0.69)***
age × MENA/S. Asia			-0.28 (0.32)
age × CEE/C. Asia			0.20 (0.30)
age × Asia			1.24 (0.45)***
$\log(P_s)$		-42.65 (15.23)***	-483.47 (116.99)***
$F \times \log(P_s)$			12.42 (2.33)***
$Age \times \log(P_s)$			9.89 (1.20)**
$Age^2 \times \log(P_s)$			-0.09 (0.01)***
$\log(P_f)$		85.90 (25.27)***	231.34 (83.87)***
$F \times \log(P_f)$			-1.38 (2.77)
$Age \times \log(P_f)$			-4.15 (2.22)*
$Age^2 \times \log(P_f)$			0.02 (0.02)
$\log(P_m)$		53.30 (20.73)***	107.80 (72.51)
$F \times \log(P_m)$			1.57 (2.63)
$Age \times \log(P_m)$			-0.97 (1.98)
$Age^2 \times \log(P_m)$			0.00 (0.02)
$\log(Y)$		145.85 (45.34)***	379.56 (109.52)***
$F \times \log(Y)$			-6.23 (2.17)***
$Age \times \log(Y)$			-3.49 (1.53)**
$Age^2 \times \log(Y)$			0.03 (0.01)***
$\log(Y)^2$		-8.77 (3.04)***	-18.97 (6.48)***
$\log(P_s) \times \log(Y)$			19.87 (10.73)*
Adjusted R^2	0.65	0.70	0.80

Note: Dependent variable is SSB intake in g/d. Standard errors are in (parentheses).
*p≤0.10; **p≤0.05; ***p≤0.01. SSA = Sub-Saharan Africa. LAC = Latin America and the Caribbean. MENA/S. Asia = Middle East, North Africa, and South Asia. CEE/C. Asia = Central Europe, Eastern Europe, and Central Asia. Asia = Asian Pacific, East Asia, and Southeast Asia. The reference region consist of high-income Western countries and a few small island states. P_s = SSB price, P_f = fruit juice price, P_m = milk price. All prices were deflated by a food price index. Y = real per capita income.

For peer review only

Supplemental Table 4

Own-price elasticities of SSB intake by age, sex, and global income decile

Income decile	Age 20	25	30	35	40	45	50	55	60	65	70	75	80
	Women												
Lowest 10%	-0.90	-0.84	-0.80	-0.77	-0.78	-0.76	-0.70	-0.71	-0.78	-0.92	-1.11	-1.40	-1.84
	(0.21)	(0.23)	(0.25)	(0.29)	(0.35)	(0.40)	(0.42)	(0.45)	(0.49)	(0.52)	(0.53)	(0.55)	(0.60)
2nd	-0.83	-0.76	-0.71	-0.67	-0.65	-0.60	-0.54	-0.52	-0.58	-0.70	-0.88	-1.16	-1.59
	(0.18)	(0.19)	(0.21)	(0.24)	(0.29)	(0.33)	(0.34)	(0.36)	(0.38)	(0.41)	(0.41)	(0.43)	(0.46)
3rd	-0.76	-0.69	-0.62	-0.56	-0.51	-0.44	-0.36	-0.33	-0.37	-0.48	-0.65	-0.92	-1.33
	(0.16)	(0.17)	(0.18)	(0.20)	(0.23)	(0.26)	(0.27)	(0.28)	(0.29)	(0.30)	(0.30)	(0.31)	(0.34)
4th	-0.70	-0.62	-0.54	-0.47	-0.40	-0.31	-0.22	-0.17	-0.19	-0.28	-0.45	-0.71	-1.10
	(0.14)	(0.15)	(0.16)	(0.17)	(0.20)	(0.21)	(0.22)	(0.22)	(0.22)	(0.22)	(0.21)	(0.21)	(0.24)
5th	-0.67	-0.58	-0.49	-0.40	-0.32	-0.22	-0.12	-0.07	-0.07	-0.15	-0.32	-0.57	-0.96
	(0.14)	(0.14)	(0.15)	(0.16)	(0.18)	(0.20)	(0.19)	(0.19)	(0.19)	(0.18)	(0.17)	(0.16)	(0.18)
6th	-0.64	-0.54	-0.45	-0.35	-0.26	-0.14	-0.04	0.02	0.02	-0.05	-0.21	-0.46	-0.84
	(0.13)	(0.13)	(0.14)	(0.15)	(0.17)	(0.19)	(0.18)	(0.18)	(0.18)	(0.17)	(0.15)	(0.14)	(0.16)
7th	-0.60	-0.51	-0.41	-0.31	-0.20	-0.07	0.04	0.10	0.11	0.05	-0.11	-0.35	-0.72
	(0.13)	(0.13)	(0.14)	(0.15)	(0.17)	(0.19)	(0.19)	(0.18)	(0.18)	(0.17)	(0.15)	(0.14)	(0.16)
8th	-0.57	-0.46	-0.36	-0.25	-0.13	0.01	0.13	0.20	0.23	0.17	0.02	-0.22	-0.58
	(0.13)	(0.13)	(0.14)	(0.16)	(0.18)	(0.20)	(0.20)	(0.20)	(0.20)	(0.19)	(0.18)	(0.17)	(0.19)
9th	-0.53	-0.42	-0.31	-0.18	-0.05	0.11	0.23	0.32	0.35	0.31	0.16	-0.08	-0.43
	(0.14)	(0.14)	(0.15)	(0.17)	(0.20)	(0.22)	(0.22)	(0.23)	(0.24)	(0.24)	(0.23)	(0.23)	(0.24)
Highest 10%	-0.47	-0.36	-0.23	-0.10	0.06	0.23	0.37	0.47	0.52	0.49	0.35	0.12	-0.22
	(0.15)	(0.16)	(0.17)	(0.19)	(0.23)	(0.26)	(0.27)	(0.29)	(0.30)	(0.32)	(0.31)	(0.32)	(0.34)
	Men												
Lowest 10%	-0.87	-0.82	-0.79	-0.79	-0.83	-0.85	-0.81	-0.83	-0.91	-1.06	-1.24	-1.50	-1.91
	(0.19)	(0.21)	(0.23)	(0.26)	(0.32)	(0.37)	(0.39)	(0.42)	(0.45)	(0.48)	(0.50)	(0.51)	(0.55)
2nd	-0.81	-0.75	-0.71	-0.69	-0.71	-0.70	-0.66	-0.66	-0.73	-0.85	-1.03	-1.28	-1.68
	(0.17)	(0.18)	(0.19)	(0.22)	(0.27)	(0.31)	(0.32)	(0.34)	(0.36)	(0.38)	(0.39)	(0.4)	(0.43)
3rd	-0.75	-0.68	-0.63	-0.60	-0.59	-0.56	-0.50	-0.48	-0.53	-0.64	-0.81	-1.06	-1.44
	(0.15)	(0.15)	(0.16)	(0.18)	(0.22)	(0.25)	(0.25)	(0.26)	(0.27)	(0.28)	(0.28)	(0.29)	(0.32)
4th	-0.69	-0.62	-0.56	-0.51	-0.48	-0.43	-0.36	-0.33	-0.36	-0.46	-0.62	-0.87	-1.24
	(0.13)	(0.14)	(0.14)	(0.16)	(0.18)	(0.20)	(0.20)	(0.20)	(0.21)	(0.21)	(0.20)	(0.21)	(0.23)
5th	-0.66	-0.58	-0.51	-0.46	-0.41	-0.34	-0.27	-0.23	-0.25	-0.34	-0.50	-0.74	-1.10
	(0.13)	(0.13)	(0.13)	(0.15)	(0.17)	(0.18)	(0.18)	(0.18)	(0.18)	(0.17)	(0.16)	(0.16)	(0.19)
6th	-0.63	-0.55	-0.48	-0.41	-0.35	-0.28	-0.19	-0.15	-0.17	-0.25	-0.40	-0.64	-0.99
	(0.12)	(0.13)	(0.13)	(0.14)	(0.16)	(0.18)	(0.17)	(0.17)	(0.17)	(0.16)	(0.14)	(0.14)	(0.17)
7th	-0.60	-0.52	-0.44	-0.37	-0.30	-0.21	-0.12	-0.07	-0.08	-0.16	-0.31	-0.54	-0.89
	(0.12)	(0.12)	(0.13)	(0.14)	(0.16)	(0.18)	(0.17)	(0.17)	(0.17)	(0.16)	(0.14)	(0.14)	(0.16)
8th	-0.57	-0.48	-0.40	-0.32	-0.23	-0.13	-0.04	0.02	0.02	-0.04	-0.19	-0.42	-0.76
	(0.12)	(0.13)	(0.13)	(0.15)	(0.17)	(0.18)	(0.18)	(0.18)	(0.18)	(0.18)	(0.17)	(0.17)	(0.19)
9th	-0.53	-0.44	-0.35	-0.26	-0.16	-0.04	0.06	0.13	0.14	0.08	-0.06	-0.29	-0.62
	(0.13)	(0.13)	(0.14)	(0.16)	(0.18)	(0.20)	(0.21)	(0.21)	(0.22)	(0.22)	(0.21)	(0.21)	(0.23)
Highest 10%	-0.49	-0.39	-0.28	-0.18	-0.06	0.07	0.19	0.27	0.29	0.25	0.11	-0.11	-0.43
	(0.14)	(0.14)	(0.15)	(0.18)	(0.21)	(0.24)	(0.25)	(0.27)	(0.28)	(0.29)	(0.29)	(0.29)	(0.31)

Note: Values are derived at median intake levels by demographic subgroup. Standard errors are in (parentheses). Price elasticities are based on 1% price changes. Income deciles are based on the national income of the 164 countries included in the study. Each decile is comprised of 16 countries except the 4 lowest deciles, which are each comprised of 17 countries. The per capita income range (PPP-adjusted in thousand $US) for each decile: (1st) $0.6-$1.5, (2nd) $1.5-$2.7, (3rd) $2.7-$5.3, (4th) $5.5-$8.0, (5th) $8.3-$10.8, (6th) $11.1-$15.2, (7th) $15.3-$20.3, (8th) $20.6-$29.4, (9th) $30.4-$40.9, and (10th) $41.3-$127.2.

For peer review only

For peer review only - http://bmjopen.bmj.com/site/about/guidelines.xhtml

Technical Appendix

Demand model and methods

To estimate SSB intake demand, we used a semi-logarithmic functional form that has been proven to be consistent with economic theory and rational consumer behavior.(1, 2) We applied a single-equation framework in this study. Prior studies have used a demand-system approach (multi-equation framework), primarily due to the adding-up property when using expenditure data (i.e., expenditures on all consumption categories “add up” to total expenditures), which results in the error terms being correlated across categories. Since this relationship does not exist with individual intakes, particularly when the correspondence between purchases and intakes is not one to one, the adopted approach is acceptable.

Many studies have used a double-log quadratic form.(3) However, a problem with the double-log form is that significant intake differences across subgroups can be lost in log conversions. A semi-log relationship allowed for a better assessment of subgroup effects on intake responsiveness. It has also been shown that semi-log models of demand are consistent with economic theory and contain the necessary information for obtaining, for instance, reliable measures of consumer welfare and the underlying preference structure of consumers.(1)

Let q_{ig} represent SSB intake by subgroup g (g: sex and age), p_i and p_j represent the price of SSBs and related good j, and Y and P represent real per capita income and overall food prices (all in country C). SSB intake demand by subgroup g in country C is specified as follows (C subscripts are omitted for convenience):

$$q_{ig} = \beta_0^* + \beta_1^* \ln(Y) + \beta_2^* \ln\left(\frac{p_i}{P}\right) + \beta_3^* \ln\left(\frac{p_j}{P}\right) + \beta_4^* \left[\ln(Y) \times \ln\left(\frac{p_i}{P}\right)\right] + \beta_5^* \ln(Y)^2 + u_{ig} \quad (1)$$

The β terms are coefficients to be estimated and u_{ig} is a random error term. The price terms (p_i and p_j) are deflated by P to discount price differences due to overall food prices and to implicitly account for the cross-price effects of intake categories other than i and j. Note that the structure of the model allows for the relationship between own-price (p_i) and intake to vary by national income level.

We accounted for age, sex, and regional differences by allowing these factors to have a direct effect on intake, as well as an additional effect through income and prices. Thus, the beta terms (β_k^*) were expanded to account for age, sex, and region interactions.

$$\beta_k^* = f(sex, age, region) \forall k \quad (2)$$

The variable *sex* is a binary (= 1 for women and 0 otherwise) and *age* is a variable ranging from 20 to 80 in 5-year intervals. We also considered age^2 to allow for nonlinear age effects and the possibility of optimal responsiveness between the youngest and oldest subgroup. We accounted for varying preferences across countries due to factors not related to income or prices by including six regional binary variables, including: Southeast Asia, East Asia, and High Income Asia Pacific (Asia) (13 countries); Central Europe, Eastern Europe, and Central Asia (CEE/C. Asia) (27); Latin America and the Caribbean (LAC) (30); Middle East, North Africa, and South Asia (MENA/S. Asia) (23); Sub-Saharan Africa (SSA) (45); and High Income/Rest of World (HIC) (26). HIC was comprised largely of Western, industrialized countries; while not geographically connected, these countries share other similarities. We included several small

For peer review only - http://bmjopen.bmj.com/site/about/guidelines.xhtml

island countries in this grouping because they were not sufficiently numerous to merit their own regional grouping.

We first estimated a model with all possible interactions and then utilized F-tests to compare that model to a series of restricted models and arrived at the final parsimonious model. All models were estimated assuming country clusters, that is, independent errors across countries but correlated errors within countries, as well as heteroskedastic-consistent errors.(4)

Given equation (1), the own-price elasticity is derived as follows:

$$\eta_{iig} = \frac{\%\Delta q_{ig}}{\%\Delta p_i} = \frac{1}{q_{ig}}[\beta_2^* + \beta_4^* \ln(Y)] \quad (3)$$

η_{iig} is the percentage change in intake (q_{ig}) (i: SSB) due to a 1% change in p_i, which should be negative since an increase in price usually results in a decrease in intake or quantity demanded. Note that if the β coefficients vary with sex, age, or region, equation (3) will vary accordingly.

Price index adjustment

We used sugar and fresh fruit price indexes as proxies for SSB and fruit juice prices, respectively. An issue with this approach is that sugar and/or fresh fruit may not account for a major share of the final product price, particularly in higher income countries. In view of this fact, we derived a multiplicative adjustment factor for the sugar and fresh fruit price indexes assuming the following quadratic relationship between the adjustment factor and real per-capita income.

$$adjustment = a + bY + cY^2 \quad (4)$$

We used a calibration method to derive values for a, b, and c. We considered the extreme case (zero income) $Y = 0$ and set $a = 1$. In this instance, equation (4) =1 and the price index value would remained unchanged:

$$adjusted\ price\ index = unadjusted\ price\ index \times 1.$$

Using information on the value-added share of farm products in the U.S. food and beverage sector, as well as qualitative information about food production costs in low-income countries, we obtained the following estimates: $b = 0.0003$ and $c = -0.0000000015$.

We adjusted the sugar and fresh fruit price indexes based on equation (4). Note that the adjustment factor starts at a value of 1 and then increases at a decreasing rate with per-capita income indicating a higher value added at higher income levels. For a country in the lowest income decile with per-capita income (Y) = $1,000, the adjustment factor is 1.3. Assuming an *unadjusted* price index value of 0.70, the *adjusted* price index = 0.91 (0.70 ×1.3). For Switzerland, a high-income country with Y=$50,963, the unadjusted sugar-price index = 0.63. At this income level, the adjustment factor = 12.39, and the adjusted sugar-price index = 7.81 (0.63 ×12.39). The unadjusted and adjusted sugar price indexes across countries are reported in supplemental figure 2.

For peer review only - http://bmjopen.bmj.com/site/about/guidelines.xhtml

References

1. LaFrance JT. Incomplete demand systems and semilogarithmic demand models. Australian J Agr Econ. 1990;34(2):118-31.
2. von Haefen RH. A complete characterization of the linear, log-linear, and semi-log incomplete demand system models. J Agr Resource Econ. 2002;27(2):281-319.
3. Sahn DE. The Effect of Price and Income Changes on Food-Energy Intake in Sri Lanka. Econ Devel Cult Change. 1988;36(2):315-40.
4. Cameron AC, Trivedi PK. Microeconometrics: Methods and Applications. Cambridge: Cambridge University Press; 2005.

For peer review only

For peer review only - http://bmjopen.bmj.com/site/about/guidelines.xhtml

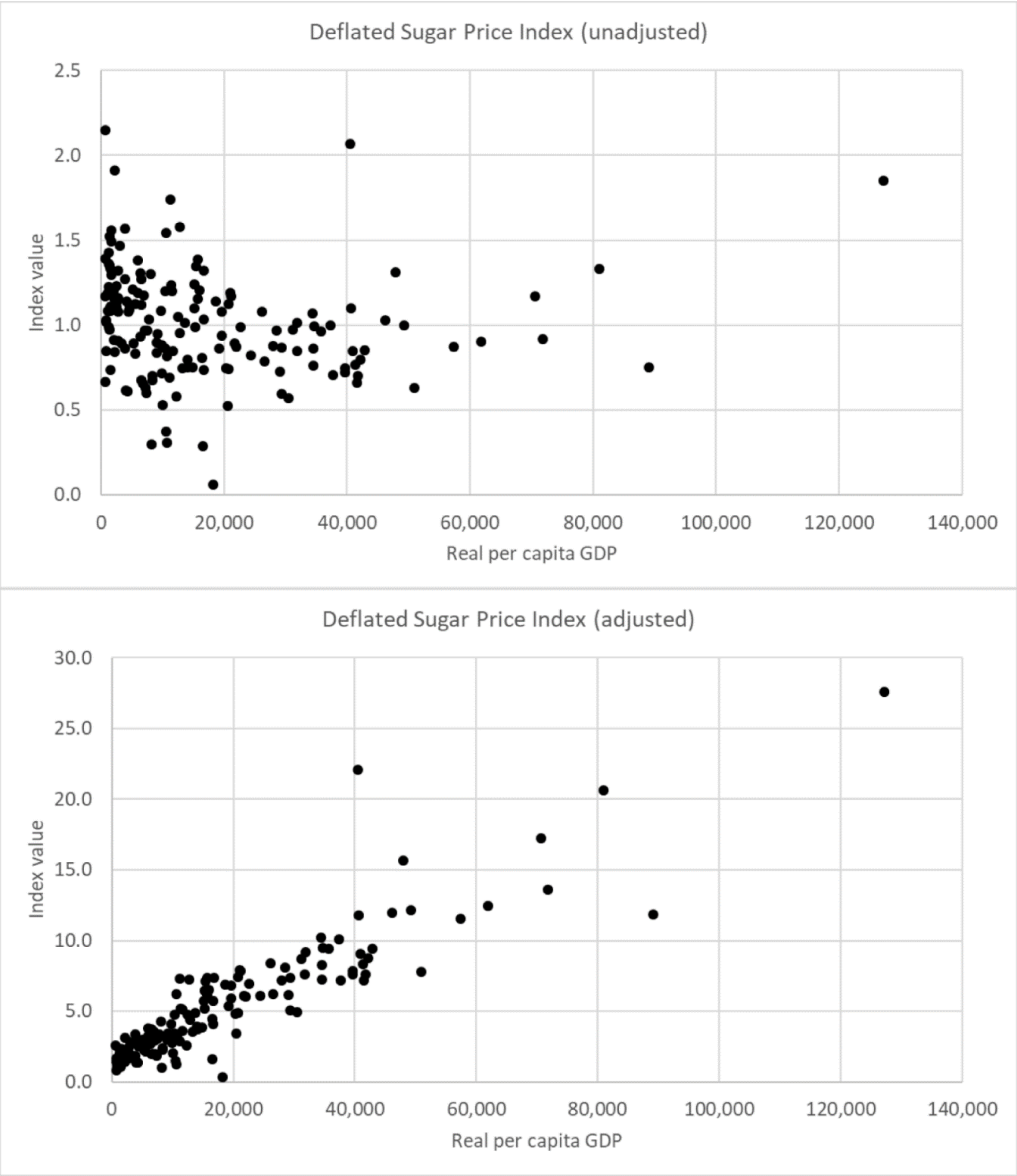

Supplemental Figure 2

Deflated sugar price index: unadjusted and adjusted

Note: prices are deflated by a total food price index to discount differences across countries due to overall food prices.

Source: World Bank International Comparison Program Data. Adjusted price index values are based on author's calculations.

BMJ Open

Global patterns in price elasticities of sugar-sweetened beverage intake and potential effectiveness of tax policy: a cross-sectional study of 164 countries by sex, age, and global-income decile

Journal:	*BMJ Open*
Manuscript ID	bmjopen-2018-026390.R3
Article Type:	Research
Date Submitted by the Author:	21-Jun-2019

Complete List of Authors:	Muhammad, Andrew; University of Tennessee Institute of Agriculture<br>Meade, Birgit; USDA Economic Research Service<br>Marquardt, David; USDA Economic Research Service<br>Mozaffarian, Dariush; Tufts University Friedman School of Nutrition Science and Policy

**Primary Subject Heading**:	Global health
Secondary Subject Heading:	Health economics, Health policy
Keywords:	elasticity, intake, global, prices, sugar-sweetened beverages, taxes

SCHOLARONE™
Manuscripts

Global patterns in price elasticities of sugar-sweetened beverage intake and potential effectiveness of tax policy: a cross-sectional study of 164 countries by sex, age, and global-income decile

Andrew Muhammad,[1] Birgit Meade,[2] David R Marquardt,[2] and Dariush Mozaffarian[3]

(Corresponding author) Andrew Muhammad, University of Tennessee Institute of Agriculture, Department of Agricultural and Resource Economics, 2621 Morgan Circle, 302 Morgan Hall Knoxville, Tennessee 37996-4518, USA

Email: amuhammad@utk.edu

[1]University of Tennessee Institute of Agriculture, Department of Agricultural and Resource Economics, Knoxville, Tennessee, USA

[2]United States Department of Agriculture, Economic Research Service, Market and Trade Economics Division, Washington, D.C., USA

[3]Tufts University, Friedman School of Nutrition Science and Policy, Boston, Massachusetts, USA

Keywords: demand, diet, elasticity, intake, global, prices, sugar-sweetened beverages, taxes

Disclaimer: The findings and conclusions in this publication are those of the author(s) and is not representative of official U.S. Department of Agriculture or U.S. Government determination or policy.

Copyright: The Corresponding Author has the right to grant on behalf of all authors and does grant on behalf of all authors, a worldwide licence (or non-exclusive for government employees) to the Publishers and its licensees in perpetuity, in all forms, formats and media (whether known now or created in the future), to i) publish, reproduce, distribute, display and store the Contribution, ii) translate the Contribution into other languages, create adaptations, reprints, include within collections and create summaries, extracts and/or, abstracts of the Contribution, iii) create any other derivative work(s) based on the Contribution, iv) to exploit all subsidiary rights in the Contribution, v) the inclusion of electronic links from the Contribution to third party

For peer review only - http://bmjopen.bmj.com/site/about/guidelines.xhtml

For peer review only

material where-ever it may be located; and, vi) licence any third party to do any or all of the above.

Contributors: AM conceptualized the study and was responsible for the study design, model estimations, and contributed to the interpretation of results. AM, DM, and BM contributed to the interpretation of results and discussion. DM wrote and edited sections describing the intake data. BM was primarily responsible for the literature review and facilitated the data agreement with the International Comparison Program, World Bank. DM provided the intake data and obtained the funding. DRM and AM were responsible for the visualizations and corresponding text. AM was the primary author, but all authors contributed to writing the manuscript. AM is the manuscript's guarantor.

Data sharing statement: Per capita income data are available in a public, open access repository: World Development Indicators Data Bank, https://databank.worldbank.org/data/reports.aspx?source=world-development-indicators. Intake and price data are available upon request at http://www.globaldietarydatabase.org/ and icp@worldbank.org, respectively.

Transparency declaration: The lead author* affirms that this manuscript is an honest, accurate, and transparent account of the study being reported; that no important aspects of the study have been omitted; and that any discrepancies from the study as planned (and, if relevant, registered) have been explained. *The manuscript's guarantor.

Funding: The Bill & Melinda Gates Foundation (project: Global Dietary Habits among Women, Price and Income Elasticities, and Validity of Food Balance Sheets).

Role of the funding source: The funders had no role in the study design, study conduct, data analysis, data interpretation, or writing of the report. All authors had full access to all the data and take responsibility for the integrity of the data and the accuracy of the analysis. The corresponding author had final responsibility to submit the report for publication.

For peer review only - http://bmjopen.bmj.com/site/about/guidelines.xhtml

Competing interests: We have read and understood BMJ policy on declaration of interests and declare that we have no competing interests.

For peer review only

For peer review only - http://bmjopen.bmj.com/site/about/guidelines.xhtml

Abstract

Objective – To quantify global relationships between sugar-sweetened beverage (SSB) intake and prices and examine the potential effectiveness of tax policy.

Design – SSB intake data by country, age, and sex from the Global Dietary Database were combined with gross domestic product (GDP) and price data from the World Bank International Comparison Program. Intake responsiveness to income and prices was estimated accounting for national income, age, and sex differences.

Setting – 164 countries.

Population – Full adult population in each country.

Main outcome measures – A consumer demand modeling framework was used to estimate the relationship between SSB intake and prices and derive own-price elasticities (measures of percentage changes in intake from a 1% price change) globally by age and sex. We simulated how a 20% tax would impact SSB intake globally. Tax policy outcomes were examined across countries by global income decile for representative age and sex subgroups.

Results – Own-price responsiveness was highest in lowest income countries, ranging from -0.70 (p<0.100) for women, age 50, to -1.91 (p<0.001) for men, age 80. In the highest income countries, responsiveness was as high as -0.49 (p<0.001) (men, age 20), but was mostly insignificant for older adults. Overall, elasticities were strongest (more negative) at the youngest and oldest age groups, and mostly insignificant for middle-aged adults, particularly in middle-income and high-income countries. Sex differences were mostly negligible. Potential intake reductions from a 20% tax in lowest income countries ranged from 14.5% (95% CI: 29.5%, -0.4%) in women, 35 ≤ age < 60, to 24.9% (44.4%, 5.3%) in men, age ≥ 60. Intake reductions decreased with country income overall, and were mostly insignificant for middle-aged adults.

Conclusions – These findings estimate the global price-responsiveness of SSB intake by age and sex, informing ongoing policy discussions on potential effects of taxes.

For peer review only - http://bmjopen.bmj.com/site/about/guidelines.xhtml

Strengths and limitations of this study

- First study to examine SSB intake and taxation in a global context, providing a better understanding of tax-policy effectiveness across the complete spectrum of countries.
- Results quantify the potential variability in influence of price on SSB intake across countries including by age and sex, suggesting that outcomes of SSB taxes may be significantly influenced by age and the income status of countries.
- Being a modeling study, the projected outcomes can only inform how taxes could affect behavior.
- Cross-country analysis of this scope rely on specific data collection initiatives that often do not occur on an annual basis and/or do not provide specific variables; proxy variables are needed when data are not available.

For peer review only

For peer review only - http://bmjopen.bmj.com/site/about/guidelines.xhtml

Introduction

Taxation of sugar-sweetened beverages (SSBs) has received growing attention, given their links to excessive weight gain and increased risk of obesity, type-2 diabetes, and other non-communicable diseases (NCDs).(1-5) Arguably, taxation is not punitive but market normalizing, as the true costs of SSBs due to public health-care expenditures and other societal costs from excessive intake are not reflected in current market prices. Thus, by increasing SSB prices relative to other foods, taxes can play a role in decreasing consumption, lowering societal costs, and improving societal wellbeing.(6, 7) Based on these considerations, a rapidly growing number of countries have implemented or announced national SSB taxes, (8, 9) including Norway in 1981 and Samoa in 1984; Australia, French Polynesia, Fiji, and Nauru between 2000 and 2007; and Finland, Hungary, France, Chile, Mexico, Barbados, St. Helena, and Dominica since 2011. In 2018, the Philippines, the United Kingdom, South Africa, the Republic of Ireland, Peru, and Norway implemented SSB taxes. Colombia and Saudi Arabia have included such taxes in recent proposals, while Bermuda, India and Indonesia are considering similar measures. In the U.S., more than 30 jurisdictions have implemented or attempted to pass SSB taxes since 2016, including San Francisco and Seattle in 2018.(10, 11) Despite their growing acceptance globally, the potential impact of SSB taxation on intake remains uncertain, particularly how it might vary across countries, and by age and sex within countries.

Most studies of SSB taxation have been limited to a small group of countries or focused on a specific country or jurisdiction where taxes have been implemented.(12-17) No study to date has examined SSB consumption and taxation in a global context. In addition, few studies have considered how SSB intake could vary depending on the price of substitute products.(18) Because expert organizations are advocating and governments are considering SSB taxation

For peer review only

For peer review only - http://bmjopen.bmj.com/site/about/guidelines.xhtml

across the globe,(19) examining demand in a global context can provide a better understanding of potential tax-policy effectiveness across the complete spectrum of countries, from most to least developed.

To investigate this issue, we examined SSB intake across 164 countries and estimated how intake differences within and across countries are influenced by the price of SSBs and substitute caloric beverages (fruit juice and milk), as well as other factors such as national income, age, and sex. Based on World Health Organization (WHO) recommendations,(19) we further simulated how SSB intake would respond to a 20% tax (price increase). Tax-policy outcomes were examined across countries by income decile for representative age and sex subgroups.

Methods

Using globally representative intake and pricing data, we implemented a consumer demand modeling framework to examine determinants of SSB intake within and across countries. The modeling framework accounted for age and sex differences and economic determinants such as own price, price of substitutes (fruit juice and milk), and real per capita income at the national level. We also considered the potential for unmeasured region-specific differences, such as taste or other preferences, by including regional binary variables. Model estimates were used to derive SSB own-price elasticities for detailed strata (age, sex, and countries by income decile), and to assess the potential impact of taxes on intake. Accounting for these factors, we report price elasticities of SSB intake (measures of the percentage change in intake from a 1% change in price), which have been a primary means of estimating potential tax-

For peer review only - http://bmjopen.bmj.com/site/about/guidelines.xhtml

policy effectiveness.(20) We also evaluated the variability in tax-policy effectiveness and examined outcomes for select age and sex subgroups and countries by income decile.

Data and sources

Data on SSB intake were derived from the 2010 Global Dietary Database (GDD), a database of global food and nutrient intakes by age (20-80 in 5-year intervals) and sex for 187 countries. The SSB category in the GDD includes intake of all sugar-sweetened beverages, including any beverage with added sugar and ≥ 50 kcal per 8 oz., such as carbonated beverages, sodas, energy drinks, fruit drinks, etc., excluding 100% juices. GDD data collection, statistical methods, data validation, and findings have been described in detail (also see http://www.globaldietarydatabase.org/).(21-25) In brief, GDD data were derived based on national and subnational dietary surveys, informed by additional information from United Nations Food and Agricultural Organization (FAO) food balance sheets data, individual-level surveys from cohort studies, household expenditure surveys when dietary surveys were not available, as well as other data sources such as the WHO Global Infobase and the WHO STEPS database.(25)

For prices, we used global price indices from the 2011 International Comparison Program (ICP) of the World Bank (see supplemental table 1).(26, 27) The ICP is a worldwide statistical initiative that produces price and expenditure data on consumer goods, services, and capital goods. The price indices used in this study are standardized to a common currency, the U.S. dollar in this case. Our choice of price variables was limited by inadequate data on a global scale. For instance, the ICP categories included milk but not SSBs and fruit juice. For SSBs, we used the ICP price index for sugar, which is justified, in part, due to sugar being a defining input.

For peer review only - http://bmjopen.bmj.com/site/about/guidelines.xhtml

Similarly, we used the ICP fresh or chilled fruit price index as a proxy for fruit juice prices. Since sugar or fresh fruit may not be a major share of the final product price, particularly in rich countries, there are limitations to these proxies. In view of this, we adjusted the sugar and fresh fruit price indexes according to national income level using information on the value-added share of farm products in U.S. food and beverage production (https://www.ers.usda.gov/data-products/food-dollar-series.aspx). This procedure resulted in relatively higher prices at higher income levels. Details are in the supplement (see supplementary information, technical appendix).

We divided each price series by an aggregate price level index for *food and nonalcoholic beverages* to adjust for differences in overall food prices across countries. This discounts any price differences across countries due to differences in overall food costs and implicitly accounts for the cross-price effects of food products not in the model.

The current analysis included 164 countries (4,264 stratum observations) having both GDD intake and ICP price data.

For national income, we used 2010 gross domestic product (GDP) data expressed in U.S. dollars per capita from the World Bank Development Indicators Database.(28) To account for differences in currency and purchasing power across economies, we used purchasing power parity (PPP) adjusted GDP. Since PPP-adjusted GDP accounts for inflationary factors across countries, we refer to our income measure as *real* per capita GDP. Income deciles were based on real per capita GDP for the 164 countries in the study.

Model and analysis

For peer review only - http://bmjopen.bmj.com/site/about/guidelines.xhtml

To estimate SSB intake demand, we applied a single-equation framework and used a semi-logarithmic functional form (see supplementary information, technical appendix).(29, 30) Many studies have used a double-log quadratic form.(31) However, a problem with the double-log form is that significant intake differences across subgroups can be lost in log conversions. A semi-log relationship allowed for a better assessment of subgroup effects on intake responsiveness. It has also been shown that semi-log models of demand are consistent with economic theory and contain the necessary information for obtaining, for instance, reliable measures of consumer welfare and the underlying preference structure of consumers.(29) Prior studies have also used a demand-system approach (multi-equation framework), primarily due to the need to account for the adding-up property when using expenditure data (i.e., expenditures on all consumption categories "add up" to total expenditures), which results in the error terms being correlated across categories. Since we are not estimating demand using an expenditure or allocation framework, the adopted approach is acceptable.

We accounted for age, sex, and regional differences by allowing these factors to have a direct effect on intake, as well as an additional effect through income and prices, including a quadratic age term to allow for nonlinear effects and the possibility of optimal responsiveness being between the youngest and oldest subgroups.

We accounted for varying preferences across countries due to factors not related to income or prices by including regional binary variables in the model: Southeast Asia, East Asia, and High Income Asia Pacific (Asia) (13 countries); Central Europe, Eastern Europe, and Central Asia (CEE/C. Asia) (27 countries); Latin America and the Caribbean (LAC) (30 countries); Middle East, North Africa, and South Asia (MENA/S. Asia) (23 countries); Sub-Saharan Africa (SSA) (45 countries); and High Income/Rest of World (HIC) (26 countries). HIC

For peer review only - http://bmjopen.bmj.com/site/about/guidelines.xhtml

was comprised largely of Western, industrialized countries; while not geographically connected, these countries share other similarities. We included several small island countries in this grouping because they were not sufficiently numerous to merit their own regional grouping (see supplemental table 2).

We utilized F-tests to compare a model including all explanatory variables and interaction terms to a series of restricted models and arrived at the final parsimonious model. Least-squares regression treats data independently and does not account for within-country correlations resulting in biased and comparatively small standard errors. Correcting for this, all models were estimated assuming country clusters, that is, independent errors across countries but correlated errors within countries, as well as heteroskedastic-consistent errors.(32) The elasticities reported in the following section were derived using the estimated coefficients from model 3 (final model) (see supplemental table 3).

Given WHO recommendations, we simulated how SSB intake would respond to a 20% tax (price increase).(19) Results were evaluated across countries by income decile for the following demographic subgroups: men and women, age < 35, 35-59, ≥ 60 years. We used probabilistic sensitivity analyses (Monte Carlo simulations) to derive 95% confidence intervals of intake responsiveness to the tax. Confidence intervals were based on the covariance matrix of the estimated coefficients, which accounted for the variability in the own-price relationship and the additional variability due to age, sex, and national income level.

Patient and public involvement

Patients and the public were not involved in the design or planning of the study.

Results

For peer review only - http://bmjopen.bmj.com/site/about/guidelines.xhtml

Global SSB intake

SSB intake levels varied significantly across countries (see supplemental figure 1) and by world region and age (figure 1). LAC had the highest median intake at 311 g/d (men) and 288 g/d (women) – almost four times the intake in SSA, and six times the lowest intake region (Asia). Across age/sex strata globally, the group with the highest median intake was young men, age 20 (209 g/d), followed closely by young women, age 20 (188 g/d). Compared to 20-year olds, median global intake in men and women, age 80, was about 75% lower. Across age and sex strata worldwide, the highest intake level was observed for men, age 20, in Trinidad and Tobago (1,239 g/d), and the lowest intake for women, age 80, in China (6 g/d). A more detailed discussion of global SSB intake by age, sex, and world region is available.(33)

SSB own-price elasticities

Given the variables in the final model, it was more appropriate to derive elasticities across country groups based on income level. We derived and compared SSB own-price elasticities across all strata jointly by age, sex, and global income decile (figure 2 and table 1; also see supplemental table 4). Note that reported values are derived at median intake levels by age and sex subgroup. Thus, observed differences across age, sex, and income decile are solely a function of own-price interactions with sex, age, and income. At any given age, SSB intake became less responsive to price changes with rising income. For instance, in women, age 20, the own-price elasticities ranged from -0.90 ($p<0.001$) for the lowest income decile to -0.47 ($p<0.001$) for the highest income decile. The decline in responsiveness became more pronounced with age. For instance, in men, age 80, the own-price elasticities ranged from -1.91 ($p<0.001$) for the lowest income decile to -0.43 ($p>0.100$) for the highest income decile. The

For peer review only - http://bmjopen.bmj.com/site/about/guidelines.xhtml

For peer review only

influence of age on SSB own-price elasticities varied depending on income status. At lower income levels, elasticities were strongest (became more negative) at older ages; but at middle and higher income levels, there was less influence of age on elasticities. The least responsive group were middle-aged adults, particularly in upper-middle and higher income deciles.

Potential impact of SSB taxes on intake

Potential reductions in median intake from a 20% tax (price increase) were largest for the lowest income decile, ranging from 14.5% (95% CI: -0.4, 29.5) to 24.1% (5.3, 44.4), depending on age and sex (table 2). Across income deciles, reductions varied less in younger adults (age < 35) – for example, ranging from 16.8% (8.6, 25.0) in young men in the lowest income decile to 7.9% (2.2, 13.6) in the highest income decile – than in older adults (e.g., men, age ≥ 60). This is consistent with the much higher baseline SSB intakes among younger adults globally (figure 1), suggesting that such intake will be significantly influenced by taxes regardless of income status. Older men and women (age ≥ 60) in the lowest income decile were estimated to be most influenced by SSB taxes, suggesting a high price-responsiveness to such a luxury in poor nations globally. Insignificant outcomes were mostly observed for middle-aged and older adults in middle and higher income deciles.

Discussion

In this global analysis of SSB intakes and prices, we identified significant price responsiveness in nearly every age, sex, and country income subgroup worldwide. We also identified significant heterogeneity in these potential responses. Price responsiveness was higher in lower income than in wealthier countries, consistent with expectations and the much higher

For peer review only - http://bmjopen.bmj.com/site/about/guidelines.xhtml

relative share of income spent on food and other necessities in low-income countries. Interestingly, the response by age varied by national income. In lower income countries, own-price responsiveness increased with age, but less so in middle and higher income countries.

Finally, our estimates of effects of a 20% tax suggested significant SSB intake reductions across income levels, particularly for young adults. Outcomes for middle-aged adults, and older adults at higher income levels, were not significant.

Strengths and limitations

This study has several strengths, the first being the extensive country coverage. We provide a global snapshot of SSB intake behavior allowing for comparisons within and across most countries. Since past studies have been limited to a single country or a select group of countries, the results of this study inform policy and decision-making beyond the current state of knowledge. Problems associated with poor diets and NCDs occur in both developing and developed countries.(34) A comparative analysis across the complete spectrum of countries can assist international organizations in developing heterogeneous strategies for specific subgroups and countries. Our use of individual intakes by age, sex, and country provides for more accurate representation of dietary behavior. Previous findings based on expenditure data may be limited by differences in expenditures and actual consumption.

Potential limitations should also be considered. First, being a modeling study, the projected outcomes can only inform how taxes could affect behavior. While an intervention study would be more fitting, interventions across 164 countries would not be feasible. Secondly, our analysis was limited by the use of price and income data at the national level. Ideally, our explanatory variables would also be at the subgroup level, reflecting that incomes typically vary

For peer review only - http://bmjopen.bmj.com/site/about/guidelines.xhtml

with age and sex, and different subgroups could face a different set of prices within a country. For instance, in countries where urban populations are relatively young, young adults could face different prices depending on market conditions in urban and rural areas. This limitation is due to the number of countries in our study. Such detailed data is not available for many countries.

While it would be ideal to have a time series of global SSB intake data, unfortunately these data do not exist. However, there is value in examining data at a point-in-time and intake in one demographic group compared to other groups, as well as comparing intake patterns across countries. Our purpose is to inform how demographic subgroups across countries might respond to price signals in form of taxes. There is value in understanding the relative responsiveness which can be gleaned from a cross-country snapshot.

The use of the global sugar prices as a proxy for SSB prices raises questions about the primary relationship of interest (SSB own-price elasticity). For higher income countries where farm production costs are a small share of the final product price, the proxy is less suitable and could result is lower "own-price" responsiveness. Accordingly, we adjusted the price index to account for higher SSB prices relative to sugar prices at higher income levels. The adjustment resulted in a 10- to 15-fold increase in the index value for higher income countries similar to the U.S. For low-income countries, adjusted and unadjusted prices were not that dissimilar (see supplemental figure 2). Using adjusted prices, we found significantly higher own-price responsiveness compared to estimates using unadjusted prices.

Comparison with other studies

Since previous research has mostly focused on higher income countries, primarily the U.S., it is difficult to compare all of our results with earlier findings. Several U.S. based studies

For peer review only - http://bmjopen.bmj.com/site/about/guidelines.xhtml

have considered how SSB consumption would respond to a tax. Given a 10% tax, the projected decrease in SSB sales ranged from 6.7% to 18.2%.(15) These results are greater than our findings for middle-aged and older adults in the highest income decile, but are closer to our findings for young adults (7.3%, women, age < 35, and 7.9%, men, age < 35), albeit we are considering a 20% tax.

Our tax outcomes are due to comparably smaller own-price elasticities. Whereas our own-price elasticity estimates for the highest income countries range from -0.5 to -0.0, meta-analyses of U.S. studies give estimates of -0.8 (-3.2 to -0.13) and -1.1 (-1.3 to -0.9).(16, 35) In a study of Mexico using data before and after implementation of a national soda tax (10%) in 2014, SSB purchases decreased by an average of 6% during the first year of implementation,(12) which is actually comparable to our findings for young adults in middle-income countries. Other studies of Latin American countries using household survey data reported estimates more comparable to our results for lower income countries.(36-38)

The fact that our estimates are relative smaller does not necessarily make them less accurate. Note that past studies have mostly used expenditure data. It has been documented that significant changes in expenditures do not always result in changes in the quantity or quality of food consumption.(39) In fact, studies have found the association between food expenditures and intake to be particularly weak and insufficient for diet and nutrition research.(40) For instance, a recent study of the SSB tax in Berkeley, California, U.S. found significant reductions in consumer spending on SSBs, increased spending on substitute beverages, but insignificant reductions in reported SSB intake.(41) Another issue is that SSBs are less perishable that other foods. When goods have an extended shelf life, individuals can take advantage of price

For peer review only - http://bmjopen.bmj.com/site/about/guidelines.xhtml

discounts, increasing expenditures when prices are low, stock piling for future consumption. Ignoring this fact can result in overestimates of own-price elasticities.(42)

Conclusion

This is the first study to examine SSB consumption and taxation in a global context. Our findings provide a better understanding of the potential effectiveness of taxes across the full spectrum of countries. Overall, we found that the influence of SSB prices on intake significantly depends on the income status of countries. Our results suggest that intake reductions (in percent) could be small or negligible for certain demographics in higher income countries. Although small in percentage terms, actual intake reductions could still be sizeable enough for high-consuming subgroups for taxes to be worth pursuing. For higher income countries, a larger tax or a tax combined with other approaches might be needed to significantly change behavior. For instance, taxes could be combined with media and education campaigns, food labeling, and other interventions.(43) For all adults in lower income countries and young adults globally, our findings indicate that taxes would be particularly effective, which is to be expected since food expenditures account for a greater share of income for these groups making them more sensitive to prices.

For peer review only - http://bmjopen.bmj.com/site/about/guidelines.xhtml

References

1. de Koning L, Malik VS, Kellogg MD, Rimm EB, Willett WC, Hu FB. Sweetened beverage consumption, incident coronary heart disease, and biomarkers of risk in men. Circulation. 2012;125(14):1735-41.

2. Fung TT, Malik V, Rexrode KM, Manson JE, Willett WC, Hu FB. Sweetened beverage consumption and risk of coronary heart disease in women. The American Journal of Clinical Nutrition. 2009;89(4):1037-42.

3. Ludwig DS, Peterson KE, Gortmaker SL. Relation between consumption of sugar-sweetened drinks and childhood obesity: a prospective, observational analysis. Lancet. 2001;357(9255):505-8.

4. Malik VS, Popkin BM, Bray GA, Després J-P, Willett WC, Hu FB. Sugar-sweetened beverages and risk of metabolic syndrome and type 2 diabetes. Diabetes Care. 2010;33(11):2477-83.

5. Mozaffarian D, Hao T, Rimm EB, Willett WC, Hu FB. Changes in diet and lifestyle and long-term weight gain in women and men. N Engl J Med. 2011;364(25):2392-404.

6. Eyles H, Ni Mhurchu C, Nghiem N, Blakely T. Food pricing strategies, population diets, and non-communicable disease: a systematic review of simulation studies. PLoS Med. 2012;9(12):e1001353.

7. World Health Organization. Using Price Policies to Promote Healthier Diets. Copenhagen, Denmark: WHO Regional Office for Europe; 2015.

8. Gustin G. Latest Soda Tax Puts Pressure on Waistlines and the World. National Geographic. 2016 March 17, 2016.

9. Grogger J. Soda Taxes and the Prices of Sodas and Other Drinks: Evidence from Mexico. National Bureau of Economic Research; 2015. Contract No.: NBER Working Paper No. 21197.

10. Paarlberg R, Mozaffarian D, Micha R. Viewpoint: Can U.S. local soda taxes continue to spread? Food Pol. 2017;71:1-7.

11. Backholer K, Vandevijvere S, Blake M, Tseng M. Sugar-sweetened beverage taxes in 2018: a year of reflections and consolidation. Public Health Nutr. 2018;21(18):3291-5.

12. Colchero MA, Popkin BM, Rivera JA, Ng SW. Beverage Purchases from Stores in Mexico Under the Excise Tax on Sugar Sweetened Beverages: Observational Study. BMJ. 2016;352:h6704.

13. Brownell KD, Farley T, Willett WC, Popkin BM, Chaloupka FJ, Thompson JW, et al. The public health and economic benefits of taxing sugar-sweetened beverages. N Engl J Med. 2009;361(16):1599-605.

For peer review only - http://bmjopen.bmj.com/site/about/guidelines.xhtml

14. Nakhimovsky SS, Feigl AB, Avila C, O'Sullivan G, Macgregor-Skinner E, Spranca M. Taxes on sugar-sweetened beverages to reduce overweight and obesity in middle-income countries: a systematic review. PLoS One. 2016;11(9):e0163358.

15. Afshin A, Peñalvo JL, Del Gobbo L, Silva J, Michaelson M, O'Flaherty M, et al. The prospective impact of food pricing on improving dietary consumption: A systematic review and meta-analysis. PLoS One. 2017;12(3):e0172277.

16. Powell LM, Chriqui JF, Khan T, Wada R, Chaloupka FJ. Assessing the potential effectiveness of food and beverage taxes and subsidies for improving public health: a systematic review of prices, demand and body weight outcomes. Obes Rev. 2013;14(2):110-28.

17. Briggs ADM, Mytton OT, Kehlbacher A, Tiffin R, Rayner M, Scarborough P. Overall and income specific effect on prevalence of overweight and obesity of 20% sugar sweetened drink tax in UK: econometric and comparative risk assessment modelling study. BMJ. 2013;347:f6189.

18. Grogger J. Soda taxes and the prices of sodas and other drinks: evidence from Mexico. Amer J Agr Econ. 2017;99(2):481-98.

19. World Health Organization. Fiscal Policies for Diet and the Prevention of Noncommunicable Diseases. Technical Meeting Report. Geneva, Switzerland: World Health Organization; 2016.

20. Mytton OT, Clarke D, Rayner M. Taxing unhealthy food and drinks to improve health. BMJ. 2012;344(7857):e2931.

21. Del Gobbo LC, Khatibzadeh S, Imamura F, Micha R, Shi P, Smith M, et al. Assessing global dietary habits: a comparison of national estimates from the FAO and the Global Dietary Database. Am J Clin Nutr. 2015;101(5):1038-46.

22. Imamura F, Micha R, Khatibzadeh S, Fahimi S, Shi P, Powles J, et al. Dietary quality among men and women in 187 countries in 1990 and 2010: a systematic assessment. Lancet Global Health. 2015;3(3):e132-e42.

23. Micha R, Kalantarian S, Wirojratana P, Byers T, Danaei G, Elmadfa I, et al. Estimating the global and regional burden of suboptimal nutrition on chronic disease: methods and inputs to the analysis. Eur J Clin Nutr. 2012;66(1):119-29.

24. Micha R, Khatibzadeh S, Shi P, Fahimi S, Lim S, Andrews KG, et al. Global, regional, and national consumption levels of dietary fats and oils in 1990 and 2010: a systematic analysis including 266 country-specific nutrition surveys. BMJ. 2014;348:g2272.

25. Khatibzadeh S, Kashaf MS, Micha R, Fahimi S, Shi P, Elmadfa I, et al. A global database of food and nutrient consumption. Bull World Health Organ. 2016;94(12):931-4E.

26. World Bank. Purchasing Power Parities and Real Expenditures of World Economies Washington, D.C.: World Bank Publications; 2014.

For peer review only

For peer review only - http://bmjopen.bmj.com/site/about/guidelines.xhtml

27. World Bank. Purchasing Power Parities and the Real Size of World Economies Washington, D.C.: World Bank Publications; 2015.

28. World Bank. World Development Indicators 2015 [Available from: http://data.worldbank.org/data-catalog/world-development-indicators.

29. LaFrance JT. Incomplete demand systems and semilogarithmic demand models. Australian J Agr Econ. 1990;34(2):118-31.

30. von Haefen RH. A complete characterization of the linear, log-linear, and semi-log incomplete demand system models. J Agr Resource Econ. 2002;27(2):281-319.

31. Sahn DE. The Effect of Price and Income Changes on Food-Energy Intake in Sri Lanka. Econ Devel Cult Change. 1988;36(2):315-40.

32. Cameron AC, Trivedi PK. Microeconometrics: Methods and Applications. Cambridge: Cambridge University Press; 2005.

33. Singh GM, Micha R, Khatibzadeh S, Shi P, Lim S, Andrews KG, et al. Global, regional, and national consumption of sugar-sweetened beverages, fruit juices, and milk: a systematic assessment of beverage intake in 187 countries. PLoS One. 2015;10(8):e0124845.

34. United Nations. Note by the Secretary-General transmitting the report of the Director-General of the World Health Organization on the prevention and control of non-communicable diseases. New York: United Nations; 2013. Report No.: A/68/650 Contract No.: A/68/650.

35. Andreyeva T, Long MW, Brownell KD. The impact of food prices on consumption: a systematic review of research on the price elasticity of demand for food. Am J Public Health. 2010;100(2):216-22.

36. Colchero MA, Salgado JC, Unar-Munguía M, Hernández-Ávila M, Rivera-Dommarco JA. Price elasticity of the demand for sugar sweetened beverages and soft drinks in Mexico. Econ Hum Biol. 2015;19:129-37.

37. Guerrero-López CM, Unar-Munguía M, Colchero MA. Price elasticity of the demand for soft drinks, other sugar-sweetened beverages and energy dense food in Chile. BMC Public Health. 2017;17(1):180.

38. Paraje G. The Effect of Price and Socio-Economic Level on the Consumption of Sugar-Sweetened Beverages (SSB): The Case of Ecuador. PLoS One. 2016;11(3):e0152260.

39. Mark Aguiar, Erik Hurst. Consumption versus Expenditure. J Polit Economy. 2005;113(5):919-48.

40. Monsivais P, Perrigue MM, Adams SL, Drewnowski A. Measuring diet cost at the individual level: a comparison of three methods. Eur J Clin Nutr. 2013;67:1220.

For peer review only

For peer review only - http://bmjopen.bmj.com/site/about/guidelines.xhtml

41. Silver LD, Ng SW, Ryan-Ibarra S, Taillie LS, Induni M, Miles DR, et al. Changes in prices, sales, consumer spending, and beverage consumption one year after a tax on sugar-sweetened beverages in Berkeley, California, US: A before-and-after study. PLoS Med. 2017;14(4):e1002283.

42. Hartmann WR. Intertemporal effects of consumption and their implications for demand elasticity estimates. Quantitative Marketing and Economics. 2006;4(4):325-49.

43. Afshin A, Micha R, Khatibzadeh S, Schmidt LA, Mozaffarian D. Dietary Policies to Reduce Noncommunicable Diseases. In: Brown GW, Yamey G, Wamala S, editors. The Handbook of Global Health Policy. West Sussex, UK: Wiley-Blackwell; 2014. p. 175-93.

For peer review only

For peer review only - http://bmjopen.bmj.com/site/about/guidelines.xhtml

Figure 1 Comparison of mean SSB intakes among adults in age, sex, and country-specific strata across world regions and globally by select age groups. *n* represents the number of age, sex, and country-specific subgroups in each stratum. Boxes represent the median intake Value and interquartile range; error bars represent the minimum and maximum values. Source: Global Dietary Database, 2010

Figure 2 Global SSB own-price elasticities by age, sex, and global income decile. Values are derived at median intake levels by demographic subgroup. Own-price elasticities are based on 1% price changes. Income deciles are based on the national income of the 164 countries included in the study. Each decile is comprised of 16 countries (except the 4 lowest deciles, which are each comprised of 17 countries). The per capita income range (PPP-adjusted in thousand $US) for each decile: (1st) $0.6-$1.5, (2nd) $1.5-$2.7, (3rd) $2.7-$5.3, (4th) $5.5-$8.0, (5th) $8.3-$10.8, (6th) $11.1-$15.2, (7th) $15.3-$20.3, (8th) $20.6-$29.4, (9th) $30.4-$40.9, and (10th) $41.3-$127.2.

For peer review only

For peer review only - http://bmjopen.bmj.com/site/about/guidelines.xhtml

Table 1 Own-price elasticities of SSB intake by age, sex, and global income decile†

Income decile‡	Age 20	Age 30	Age 40	Age 50	Age 60	Age 70	Age 80	Population-weighted average
	Women							
Lowest 10%	-0.90 (0.21)***	-0.80 (0.25)***	-0.78 (0.35)**	-0.70 (0.42)*	-0.78 (0.49)	-1.11 (0.53)**	-1.84 (0.60)***	-0.82 (0.30)***
2nd	-0.83 (0.18)***	-0.71 (0.21)***	-0.65 (0.29)**	-0.54 (0.34)	-0.58 (0.38)	-0.88 (0.41)**	-1.59 (0.46)***	-0.71 (0.25)***
3rd	-0.76 (0.16)***	-0.62 (0.18)***	-0.51 (0.23)**	-0.36 (0.27)	-0.37 (0.29)	-0.65 (0.30)**	-1.33 (0.34)***	-0.59 (0.21)***
4th	-0.70 (0.14)***	-0.54 (0.16)***	-0.40 (0.20)**	-0.22 (0.22)	-0.19 (0.22)	-0.45 (0.21)**	-1.10 (0.24)***	-0.49 (0.17)***
5th	-0.67 (0.14)***	-0.49 (0.15)***	-0.32 (0.18)*	-0.12 (0.19)	-0.07 (0.19)	-0.32 (0.17)*	-0.96 (0.18)***	-0.40 (0.16)**
6th	-0.64 (0.13)***	-0.45 (0.14)***	-0.26 (0.17)	-0.04 (0.18)	0.02 (0.18)	-0.21 (0.15)	-0.84 (0.16)***	-0.33 (0.15)**
7th	-0.60 (0.13)***	-0.41 (0.14)***	-0.20 (0.17)	0.04 (0.19)	0.11 (0.18)	-0.11 (0.15)	-0.72 (0.16)***	-0.27 (0.11)**
8th	-0.57 (0.13)***	-0.36 (0.14)**	-0.13 (0.18)	0.13 (0.20)	0.23 (0.20)	0.02 (0.18)	-0.58 (0.19)***	-0.22 (0.11)**
9th	-0.53 (0.14)***	-0.31 (0.15)**	-0.05 (0.20)	0.23 (0.22)	0.35 (0.24)	0.16 (0.23)	-0.43 (0.24)*	-0.15 (0.06)**
Highest 10%	-0.47 (0.15)***	-0.23 (0.17)	0.06 (0.23)	0.37 (0.27)	0.52 (0.30)	0.35 (0.31)	-0.22 (0.34)	-0.11 (0.07)
	Men							
Lowest 10%	-0.87 (0.19)***	-0.79 (0.23)***	-0.83 (0.32)***	-0.81 (0.39)**	-0.91 (0.45)**	-1.24 (0.50)**	-1.91 (0.55)***	-0.84 (0.30)***
2nd	-0.81 (0.17)***	-0.71 (0.19)***	-0.71 (0.27)***	-0.66 (0.32)**	-0.73 (0.36)**	-1.03 (0.39)***	-1.68 (0.43)***	-0.76 (0.23)***
3rd	-0.75 (0.15)***	-0.63 (0.16)***	-0.59 (0.22)***	-0.50 (0.25)**	-0.53 (0.27)*	-0.81 (0.28)***	-1.44 (0.32)***	-0.59 (0.21)***
4th	-0.69 (0.13)***	-0.56 (0.14)***	-0.48 (0.18)***	-0.36 (0.20)*	-0.36 (0.21)*	-0.62 (0.20)***	-1.24 (0.23)***	-0.54 (0.16)***
5th	-0.66 (0.13)***	-0.51 (0.13)***	-0.41 (0.17)**	-0.27 (0.18)	-0.25 (0.18)	-0.50 (0.16)***	-1.10 (0.19)***	-0.40 (0.16)**
6th	-0.63 (0.12)***	-0.48 (0.13)***	-0.35 (0.16)**	-0.19 (0.17)	-0.17 (0.17)	-0.40 (0.14)***	-0.99 (0.17)***	-0.42 (0.14)***
7th	-0.60 (0.12)***	-0.44 (0.13)***	-0.30 (0.16)*	-0.12 (0.17)	-0.08 (0.17)	-0.31 (0.14)**	-0.89 (0.16)***	-0.28 (0.13)**
8th	-0.57 (0.12)***	-0.40 (0.13)***	-0.23 (0.17)	-0.04 (0.18)	0.02 (0.18)	-0.19 (0.17)	-0.76 (0.19)***	-0.29 (0.12)**
9th	-0.53 (0.13)***	-0.35 (0.14)**	-0.16 (0.18)	0.06 (0.21)	0.14 (0.22)	-0.06 (0.21)	-0.62 (0.23)***	-0.16 (0.09)*
Highest 10%	-0.49 (0.14)***	-0.28 (0.15)*	-0.06 (0.21)	0.19 (0.25)	0.29 (0.28)	0.11 (0.29)	-0.43 (0.31)	-0.16 (0.10)

Values are derived at median intake levels by demographic subgroup. Standard errors are in (parenthesis). Population weights by sex, age, and income status were obtained from the World Development Indicators Data Bank: https://databank.worldbank.org/data/reports.aspx?source=world-development-indicators#.
*p≤0.10; **p≤0.05; ***p≤0.01.
†Price elasticities are based on 1% price changes. For instance, given a 1% SSB price increase in the lowest income countries, intake by women, age 20 falls by 0.90%. ‡Income deciles are based on the national income of the 164 countries included in the study. Each decile is comprised of 16 countries (except the 4 lowest deciles, which are each comprised of 17 countries). The per capita income range (PPP-adjusted in thousand $US) for each decile: (1st) $0.6-$1.5, (2nd) $1.5-$2.7, (3rd) $2.7-$5.3, (4th) $5.5-$8.0, (5th) $8.3-$10.8, (6th) $11.1-$15.2, (7th) $15.3-$20.3, (8th) $20.6-$29.4, (9th) $30.4-$40.9, and (10th) $41.3-$127.2.

For peer review only - http://bmjopen.bmj.com/site/about/guidelines.xhtml

Table 2 Potential impact of a 20% tax (price increase) on SSB intake by age, sex, and global income decile.

Income decile†	Women age < 35	Men age < 35	Women 35 ≤ age < 60	Men 35 ≤ age < 60	Women age ≥ 60	Men age ≥ 60
	Percentage change in intake (95% CI)					
Lowest 10%	-17.1 (-26.1 to -8.1)	-16.8 (-25.0 to -8.6)	-14.5 (-29.5 to 0.4)	-15.9 (-29.5 to -2.2)	-22.3 (-43.2 to -1.4)	-24.9 (-44.4 to -5.3)
2nd	-15.6 (-23.3 to -7.9)	-15.4 (-22.5 to -8.3)	-11.6 (-23.8 to 0.7)	-13.2 (-24.3 to -1.9)	-17.7 (-33.9 to -1.4)	-20.6 (-35.9 to -5.3)
3rd	-14.0 (-20.6 to -7.4)	-14.0 (-20.1 to -7.8)	-8.5 (-18.3 to 1.3)	-10.4 (-19.4 to -1.4)	-13.0 (-24.8 to -1.2)	-16.3 (-27.4 to -5.1)
4th	-12.6 (-18.5 to -6.7)	-12.7 (-18.2 to -7.3)	-5.9 (-14.0 to 2.2)	-8.0 (-15.5 to -0.5)	-9.0 (-17.3 to -0.7)	-12.5 (-20.5 to -4.6)
5th	-11.7 (-17.3 to -6.2)	-11.9 (-17.1 to -6.7)	-4.2 (-11.6 to 3.2)	-6.5 (-13.2 to 0.3)	-6.3 (-12.9 to 0.3)	-10.1 (-16.4 to -3.7)
6th	-11.0 (-16.4 to -5.6)	-11.3 (-16.3 to -6.2)	-2.8 (-9.9 to 4.3)	-5.2 (-11.6 to 1.3)	-4.2 (-10 to 1.7)	-8.1 (-13.7 to -2.4)
7th	-10.3 (-15.7 to -5.0)	-10.6 (-15.6 to -5.7)	-1.4 (-8.5 to 5.6)	-4.0 (-10.4 to 2.5)	-2.1 (-8 to 3.9)	-6.2 (-11.8 to -0.4)
8th	-9.5 (-14.9 to -4.1)	-9.9 (-14.9 to -4.9)	0.2 (-7.3 to 7.6)	-2.5 (-9.2 to 4.3)	0.4 (-6.6 to 7.5)	-3.8 (-10.4 to 2.8)
9th	-8.5 (-14.2 to -2.9)	-9.0 (-14.3 to -3.8)	2.0 (-6.3 to 10.3)	-0.8 (-8.3 to 6.7)	3.2 (-5.8 to 12.3)	-1.2 (-9.6 to 7.2)
Highest 10%	-7.3 (-13.5 to -1.1)	-7.9 (-13.6 to -2.2)	4.5 (-5.4 to 14.3)	1.4 (-7.6 to 10.3)	7.0 (-5.3 to 19.3)	2.2 (-9.1 to 13.6)

Values are reductions from median intake levels for each demographic subgroup.
†Income deciles are based on the national income of the 164 countries included in the study. Each decile is comprised of 16 countries except the 4 lowest deciles, which are each comprised of 17 countries. The per capita income range (PPP-adjusted in thousand $US) for each decile: (1st) $0.6-$1.5, (2nd) $1.5-$2.7, (3rd) $2.7-$5.3, (4th) $5.5-$8.0, (5th) $8.3-$10.8, (6th) $11.1-$15.2, (7th) $15.3-$20.3, (8th) $20.6-$29.4, (9th) $30.4-$40.9, and (10th) $41.3-$127.2.

For peer review only

For peer review only - http://bmjopen.bmj.com/site/about/guidelines.xhtml

<Insert Figure1.png file here>

Figure 1 Comparison of mean SSB intakes among adults in age, sex, and country-specific strata across world regions and globally by select age groups. *n* represents the number of age, sex, and country-specific subgroups in each stratum. Boxes represent the median intake value and interquartile range; error bars represent the minimum and maximum values. Source: Global Dietary Database, 2010

For peer review only

For peer review only - http://bmjopen.bmj.com/site/about/guidelines.xhtml

<Insert Figure2.png file here>

Figure 2 Global SSB own-price elasticities by age, sex, and global income decile. Values are derived at median intake levels by demographic subgroup. Own-price elasticities are based on 1% price changes. Income deciles are based on the national income of the 164 countries included in the study. Each decile is comprised of 16 countries (except the 4 lowest deciles, which are each comprised of 17 countries). The per capita income range (PPP-adjusted in thousand $US) for each decile: (1st) $0.6-$1.5, (2nd) $1.5-$2.7, (3rd) $2.7-$5.3, (4th) $5.5-$8.0, (5th) $8.3-$10.8, (6th) $11.1-$15.2, (7th) $15.3-$20.3, (8th) $20.6-$29.4, (9th) $30.4-$40.9, and (10th) $41.3-$127.2.

For peer review only

For peer review only - http://bmjopen.bmj.com/site/about/guidelines.xhtml

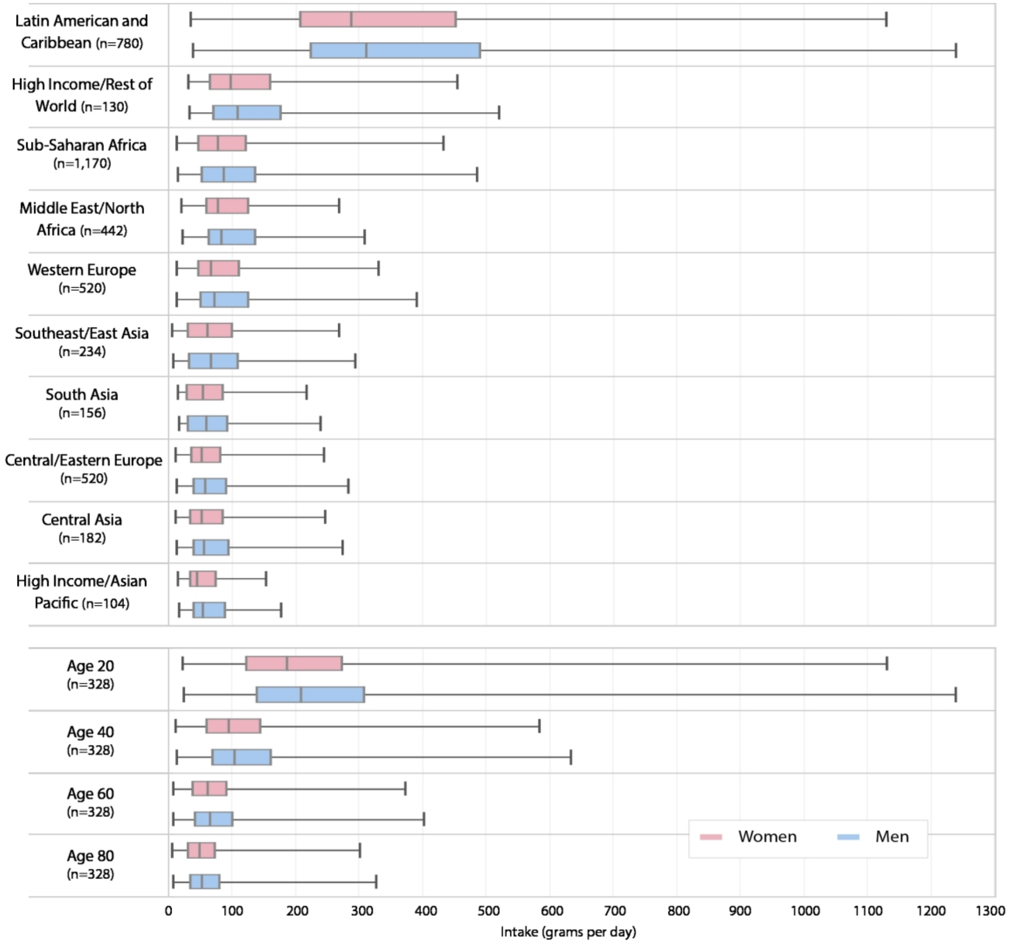

Figure 1 Comparison of mean SSB intakes among adults in age, sex, and country-specific strata across world regions and globally by select age groups. n represents the number of age, sex, and country-specific subgroups in each stratum. Boxes represent the median intake value and interquartile range; error bars represent the minimum and maximum values.
Source: Global Dietary Database, 2010

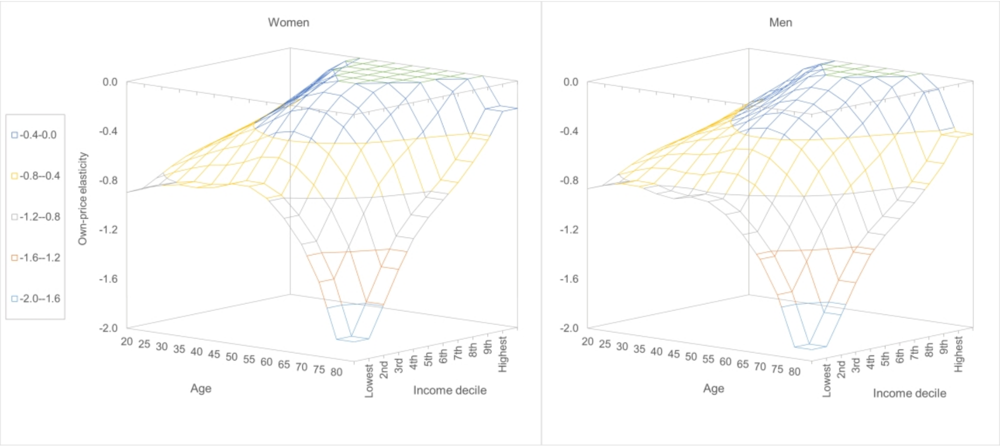

Figure 2 Global SSB own-price elasticities by age, sex, and global income decile. Values are derived at median intake levels by demographic subgroup. Own-price elasticities are based on 1% price changes. Income deciles are based on the national income of the 164 countries included in the study. Each decile is comprised of 16 countries (except the 4 lowest deciles, which are each comprised of 17 countries). The per capita income range (PPP-adjusted in thousand $US) for each decile: (1st) $0.6-$1.5, (2nd) $1.5-$2.7, (3rd) $2.7-$5.3, (4th) $5.5-$8.0, (5th) $8.3-$10.8, (6th) $11.1-$15.2, (7th) $15.3-$20.3, (8th) $20.6-$29.4, (9th) $30.4-$40.9, and (10th) $41.3-$127.2.

Supplementary Information

Global patterns in price elasticities of sugar-sweetened beverage intake and potential effectiveness of tax policy: a cross-sectional study of 164 countries by sex, age, and global-income decile

Andrew Muhammad,[1] Birgit Meade,[2] David R Marquardt,[2] and Dariush Mozaffarian[3]

[1]University of Tennessee Institute of Agriculture, Department of Agricultural and Resource Economics, Knoxville, Tennessee, USA
[2]United States Department of Agriculture, Economic Research Service, Market and Trade Economics Division, Washington, D.C., USA
[3]Tufts University, Friedman School of Nutrition Science and Policy, Boston, Massachusetts, USA

Supplemental Figure 1	Mean SSB intake across countries in adults, age ≥ 20
Supplemental Table 1	Description of ICP food-price categories
Supplemental Table 2	Countries included in study by region (aggregate regions used for estimation)
Supplemental Table 3	Demand model estimates for SSB intake
Supplemental Table 4	Own-price elasticities of SSB intake by age, sex, and global income decile
Technical Appendix	Demand model and methods
Supplemental Figure 2	Deflated sugar price index: unadjusted and adjusted

For peer review only

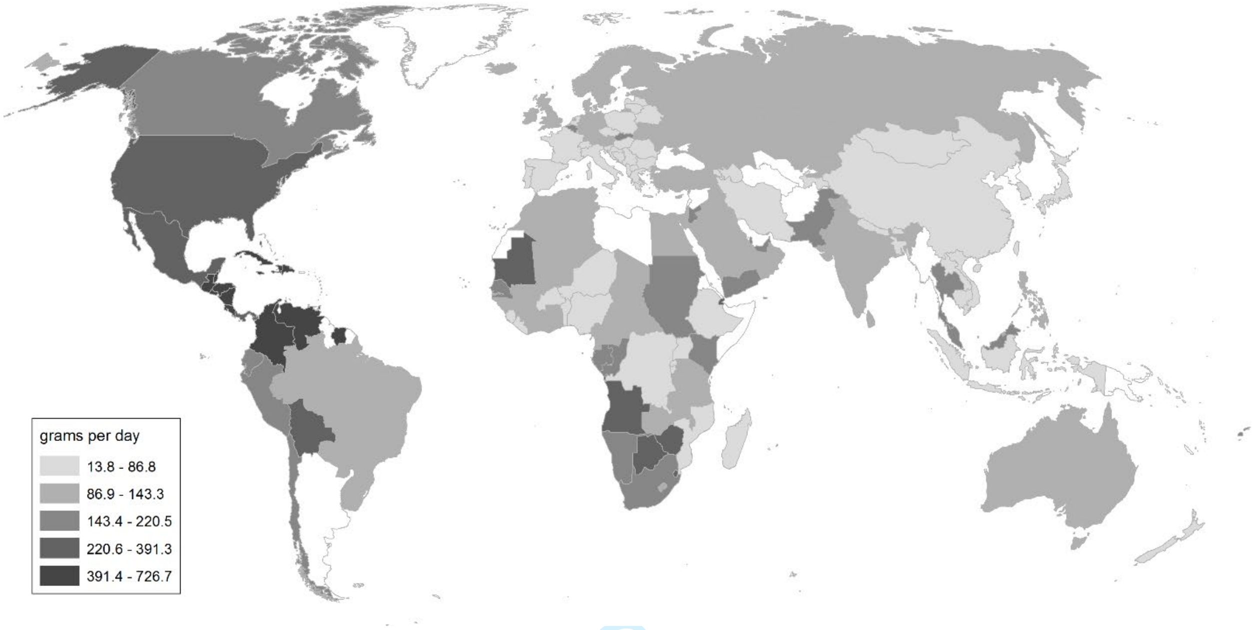

Supplemental Figure 1

Mean SSB intake across countries in adults, age ≥ 20

Map was created using ArcGIS® and ArcMap™ software by Esri. ArcGIS® and ArcMap™ are the intellectual property of Esri and are used herein under license. According to Esri citation guidelines, geodata is redistributable with value-added software applications developed by Esri on a royalty-free basis with proper metadata and copyright attribution to the respective data vendor/vendors. See Esri citation guide for more details: http://help.arcgis.com/en/arcgisdesktop/10.0/help/index.html#//001z00000003000000.htm.

Data source: Global Dietary Database, 2010

Supplemental Table 1

Description of ICP food-price categories

ICP food-price category
• *Fresh or chilled fruit* – All fresh or chilled fruit including melons and water melons; excludes vegetables grown for their fruit such as cucumbers and tomatoes.
• *Fresh milk* – Raw milk; pasteurised or sterilised milk; includes whole and low fat milk; recombined or reconstituted milk; soya milk.
• *Sugar* – Cane or beet sugar, unrefined or refined, powdered, crystallised or in lumps; includes artificial sugar substitutes.

For peer review only

For peer review only - http://bmjopen.bmj.com/site/about/guidelines.xhtml

Supplemental Table 2

Countries included in study by region (aggregate regions used for estimation)

Region	Countries
Southeast Asia, East Asia, and High Income Asia Pacific (Asia)<br>(13 countries)	Brunei Darussalam, Cambodia, China, Indonesia, Japan, South Korea, Laos, Malaysia, Maldives, Philippines, Singapore, Thailand, and Vietnam.
Central Europe, Eastern Europe, and Central Asia (CEE/C. Asia)<br>(27 countries)	Albania, Armenia, Azerbaijan, Belarus, Bosnia and Herzegovina, Bulgaria, Croatia, Czech Republic, Estonia, Georgia, Hungary, Kazakhstan, Kyrgyz Republic, Latvia, Lithuania, Macedonia, Moldova, Mongolia, Montenegro, Poland, Romania, Russia, Serbia, Slovak Republic, Slovenia, Tajikistan, and Ukraine.
Latin America and the Caribbean (LAC)<br>(30 countries)	Antigua and Barbuda, Bahamas, Barbados, Belize, Bolivia, Brazil, Chile, Colombia, Costa Rica, Cuba, Dominica, Dominican Republic, Ecuador, El Salvador, Grenada, Guatemala, Haiti, Honduras, Jamaica, Mexico, Nicaragua, Panama, Paraguay, Peru, St. Lucia, St. Vincent and Grenadines, Suriname, Trinidad and Tobago, Uruguay, and Venezuela.
Middle East, North Africa, and South Asia (MENA/S. Asia)<br>(23 countries)	Algeria, Bahrain, Bangladesh, Bhutan, Egypt, India, Iran, Iraq, Israel, Jordan, Kuwait, Morocco, Nepal, Oman, Pakistan, Qatar, Saudi Arabia, Sri Lanka, Tunisia, Turkey, United Arab Emirates, West Bank and Gaza, and Yemen.
Sub-Saharan Africa (SSA)<br>(45 countries)	Angola, Benin, Botswana, Burkina Faso, Burundi, Cameroon, Cape Verde, Central African Republic, Chad, Comoros, Congo, Côte d'Ivoire, Djibouti, DR Congo, Equatorial Guinea, Ethiopia, Gabon, Gambia, Ghana, Guinea, Guinea-Bissau, Kenya, Lesotho, Liberia, Madagascar, Malawi, Mali, Mauritania, Mauritius, Mozambique, Namibia, Niger, Nigeria, Rwanda, São Tomé and Principe, Senegal, Sierra Leone, South Africa, Sudan, Swaziland, Tanzania, Togo, Uganda, Zambia, and Zimbabwe.
High Income/Rest of World (HIC)<br>(26 countries)	Australia, Austria, Belgium, Canada, Cyprus, Denmark, Fiji, Finland, France, Germany, Greece, Iceland, Ireland, Italy, Luxembourg, Malta, Netherlands, New Zealand, Norway, Portugal, Seychelles, Spain, Sweden, Switzerland, United Kingdom, and United States.

For peer review only

Supplemental Table 3

Demand model estimates for SSB intake

Variable	model 1 estimate (SE)	model 2 estimate (SE)	model 3 (final model) estimate (SE)
constant	436.63 (25.47)***	-784.78 (378.46)**	-1,398.66 (535.82)***
female (F)	-13.36 (0.82)***	-13.36 (0.82)***	28.17 (15.92)*
age	-10.87 (0.74)***	-10.87 (0.74)***	14.06 (11.51)
age^2	0.08 (0.01)***	0.08 (0.01)***	-0.15 (0.09)*
SSA	1.83 (11.47)	35.51 (17.22)**	78.19 (37.85)**
LAC	258.41 (26.38)***	251.12 (28.01)***	546.89 (62.00)***
MENA/S. Asia	-10.05 (10.57)	7.45 (13.56)	21.20 (28.71)
CEE/C. Asia	-32.87 (9.99)***	-18.44 (12.59)	-28.38 (26.74)
Asia	-26.85 (14.66)*	-59.53 (19.50)***	-121.47 (41.21)***
age × SSA			-0.85 (0.42)**
age × LAC			-5.92 (0.69)***
age × MENA/S. Asia			-0.28 (0.32)
age × CEE/C. Asia			0.20 (0.30)
age × Asia			1.24 (0.45)***
$\log(P_s)$		-42.65 (15.23)***	-483.47 (116.99)***
$F \times \log(P_s)$			12.42 (2.33)***
$Age \times \log(P_s)$			9.89 (1.20)**
$Age^2 \times \log(P_s)$			-0.09 (0.01)***
$\log(P_f)$		85.90 (25.27)***	231.34 (83.87)***
$F \times \log(P_f)$			-1.38 (2.77)
$Age \times \log(P_f)$			-4.15 (2.22)*
$Age^2 \times \log(P_f)$			0.02 (0.02)
$\log(P_m)$		53.30 (20.73)***	107.80 (72.51)
$F \times \log(P_m)$			1.57 (2.63)
$Age \times \log(P_m)$			-0.97 (1.98)
$Age^2 \times \log(P_m)$			0.00 (0.02)
$\log(Y)$		145.85 (45.34)***	379.56 (109.52)***
$F \times \log(Y)$			-6.23 (2.17)***
$Age \times \log(Y)$			-3.49 (1.53)**
$Age^2 \times \log(Y)$			0.03 (0.01)***
$\log(Y)^2$		-8.77 (3.04)***	-18.97 (6.48)***
$\log(P_s) \times \log(Y)$			19.87 (10.73)*
Adjusted R^2	0.65	0.70	0.80

Note: Dependent variable is SSB intake in g/d. Standard errors are in (parentheses).
*p≤0.10; **p≤0.05; ***p≤0.01. SSA = Sub-Saharan Africa. LAC = Latin America and the Caribbean. MENA/S. Asia = Middle East, North Africa, and South Asia. CEE/C. Asia = Central Europe, Eastern Europe, and Central Asia. Asia = Asian Pacific, East Asia, and Southeast Asia. The reference region consist of high-income Western countries and a few small island states. P_s = SSB price, P_f = fruit juice price, P_m = milk price. All prices were deflated by a food price index. Y = real per capita income.

For peer review only

For peer review only - http://bmjopen.bmj.com/site/about/guidelines.xhtml

Supplemental Table 4

Own-price elasticities of SSB intake by age, sex, and global income decile

Income decile	Age 20	25	30	35	40	45	50	55	60	65	70	75	80
	Women												
Lowest	-0.90	-0.84	-0.80	-0.77	-0.78	-0.76	-0.70	-0.71	-0.78	-0.92	-1.11	-1.40	-1.84
10%	(0.21)	(0.23)	(0.25)	(0.29)	(0.35)	(0.40)	(0.42)	(0.45)	(0.49)	(0.52)	(0.53)	(0.55)	(0.60)
2nd	-0.83	-0.76	-0.71	-0.67	-0.65	-0.60	-0.54	-0.52	-0.58	-0.70	-0.88	-1.16	-1.59
	(0.18)	(0.19)	(0.21)	(0.24)	(0.29)	(0.33)	(0.34)	(0.36)	(0.38)	(0.41)	(0.41)	(0.43)	(0.46)
3rd	-0.76	-0.69	-0.62	-0.56	-0.51	-0.44	-0.36	-0.33	-0.37	-0.48	-0.65	-0.92	-1.33
	(0.16)	(0.17)	(0.18)	(0.20)	(0.23)	(0.26)	(0.27)	(0.28)	(0.29)	(0.30)	(0.30)	(0.31)	(0.34)
4th	-0.70	-0.62	-0.54	-0.47	-0.40	-0.31	-0.22	-0.17	-0.19	-0.28	-0.45	-0.71	-1.10
	(0.14)	(0.15)	(0.16)	(0.17)	(0.20)	(0.21)	(0.22)	(0.22)	(0.22)	(0.22)	(0.21)	(0.21)	(0.24)
5th	-0.67	-0.58	-0.49	-0.40	-0.32	-0.22	-0.12	-0.07	-0.07	-0.15	-0.32	-0.57	-0.96
	(0.14)	(0.14)	(0.15)	(0.16)	(0.18)	(0.20)	(0.19)	(0.19)	(0.19)	(0.18)	(0.17)	(0.16)	(0.18)
6th	-0.64	-0.54	-0.45	-0.35	-0.26	-0.14	-0.04	0.02	0.02	-0.05	-0.21	-0.46	-0.84
	(0.13)	(0.13)	(0.14)	(0.15)	(0.17)	(0.19)	(0.18)	(0.18)	(0.18)	(0.17)	(0.15)	(0.14)	(0.16)
7th	-0.60	-0.51	-0.41	-0.31	-0.20	-0.07	0.04	0.10	0.11	0.05	-0.11	-0.35	-0.72
	(0.13)	(0.13)	(0.14)	(0.15)	(0.17)	(0.19)	(0.19)	(0.18)	(0.18)	(0.17)	(0.15)	(0.14)	(0.16)
8th	-0.57	-0.46	-0.36	-0.25	-0.13	0.01	0.13	0.20	0.23	0.17	0.02	-0.22	-0.58
	(0.13)	(0.13)	(0.14)	(0.16)	(0.18)	(0.20)	(0.20)	(0.20)	(0.20)	(0.19)	(0.18)	(0.17)	(0.19)
9th	-0.53	-0.42	-0.31	-0.18	-0.05	0.11	0.23	0.32	0.35	0.31	0.16	-0.08	-0.43
	(0.14)	(0.14)	(0.15)	(0.17)	(0.20)	(0.22)	(0.22)	(0.23)	(0.24)	(0.24)	(0.23)	(0.23)	(0.24)
Highest	-0.47	-0.36	-0.23	-0.10	0.06	0.23	0.37	0.47	0.52	0.49	0.35	0.12	-0.22
10%	(0.15)	(0.16)	(0.17)	(0.19)	(0.23)	(0.26)	(0.27)	(0.29)	(0.30)	(0.32)	(0.31)	(0.32)	(0.34)
	Men												
Lowest	-0.87	-0.82	-0.79	-0.79	-0.83	-0.85	-0.81	-0.83	-0.91	-1.06	-1.24	-1.50	-1.91
10%	(0.19)	(0.21)	(0.23)	(0.26)	(0.32)	(0.37)	(0.39)	(0.42)	(0.45)	(0.48)	(0.50)	(0.51)	(0.55)
2nd	-0.81	-0.75	-0.71	-0.69	-0.71	-0.70	-0.66	-0.66	-0.73	-0.85	-1.03	-1.28	-1.68
	(0.17)	(0.18)	(0.19)	(0.22)	(0.27)	(0.31)	(0.32)	(0.34)	(0.36)	(0.38)	(0.39)	(0.4)	(0.43)
3rd	-0.75	-0.68	-0.63	-0.60	-0.59	-0.56	-0.50	-0.48	-0.53	-0.64	-0.81	-1.06	-1.44
	(0.15)	(0.15)	(0.16)	(0.18)	(0.22)	(0.25)	(0.25)	(0.26)	(0.27)	(0.28)	(0.28)	(0.29)	(0.32)
4th	-0.69	-0.62	-0.56	-0.51	-0.48	-0.43	-0.36	-0.33	-0.36	-0.46	-0.62	-0.87	-1.24
	(0.13)	(0.14)	(0.14)	(0.16)	(0.18)	(0.20)	(0.20)	(0.20)	(0.21)	(0.21)	(0.20)	(0.21)	(0.23)
5th	-0.66	-0.58	-0.51	-0.46	-0.41	-0.34	-0.27	-0.23	-0.25	-0.34	-0.50	-0.74	-1.10
	(0.13)	(0.13)	(0.13)	(0.15)	(0.17)	(0.18)	(0.18)	(0.18)	(0.18)	(0.17)	(0.16)	(0.16)	(0.19)
6th	-0.63	-0.55	-0.48	-0.41	-0.35	-0.28	-0.19	-0.15	-0.17	-0.25	-0.40	-0.64	-0.99
	(0.12)	(0.13)	(0.13)	(0.14)	(0.16)	(0.18)	(0.17)	(0.17)	(0.17)	(0.16)	(0.14)	(0.14)	(0.17)
7th	-0.60	-0.52	-0.44	-0.37	-0.30	-0.21	-0.12	-0.07	-0.08	-0.16	-0.31	-0.54	-0.89
	(0.12)	(0.12)	(0.13)	(0.14)	(0.16)	(0.18)	(0.17)	(0.17)	(0.17)	(0.16)	(0.14)	(0.14)	(0.16)
8th	-0.57	-0.48	-0.40	-0.32	-0.23	-0.13	-0.04	0.02	0.02	-0.04	-0.19	-0.42	-0.76
	(0.12)	(0.13)	(0.13)	(0.15)	(0.17)	(0.18)	(0.18)	(0.18)	(0.18)	(0.18)	(0.17)	(0.17)	(0.19)
9th	-0.53	-0.44	-0.35	-0.26	-0.16	-0.04	0.06	0.13	0.14	0.08	-0.06	-0.29	-0.62
	(0.13)	(0.13)	(0.14)	(0.16)	(0.18)	(0.20)	(0.21)	(0.21)	(0.22)	(0.22)	(0.21)	(0.21)	(0.23)
Highest	-0.49	-0.39	-0.28	-0.18	-0.06	0.07	0.19	0.27	0.29	0.25	0.11	-0.11	-0.43
10%	(0.14)	(0.14)	(0.15)	(0.18)	(0.21)	(0.24)	(0.25)	(0.27)	(0.28)	(0.29)	(0.29)	(0.29)	(0.31)

Note: Values are derived at median intake levels by demographic subgroup. Standard errors are in (parentheses). Price elasticities are based on 1% price changes. Income deciles are based on the national income of the 164 countries included in the study. Each decile is comprised of 16 countries except the 4 lowest deciles, which are each comprised of 17 countries. The per capita income range (PPP-adjusted in thousand $US) for each decile: (1st) $0.6-$1.5, (2nd) $1.5-$2.7, (3rd) $2.7-$5.3, (4th) $5.5-$8.0, (5th) $8.3-$10.8, (6th) $11.1-$15.2, (7th) $15.3-$20.3, (8th) $20.6-$29.4, (9th) $30.4-$40.9, and (10th) $41.3-$127.2.

For peer review only - http://bmjopen.bmj.com/site/about/guidelines.xhtml

Technical Appendix

Demand model and methods

To estimate SSB intake demand, we used a semi-logarithmic functional form that has been proven to be consistent with economic theory and rational consumer behavior.(1, 2) We applied a single-equation framework in this study. Prior studies have used a demand-system approach (multi-equation framework), primarily due to the adding-up property when using expenditure data (i.e., expenditures on all consumption categories "add up" to total expenditures), which results in the error terms being correlated across categories. Since this relationship does not exist with individual intakes, particularly when the correspondence between purchases and intakes is not one to one, the adopted approach is acceptable.

Many studies have used a double-log quadratic form.(3) However, a problem with the double-log form is that significant intake differences across subgroups can be lost in log conversions. A semi-log relationship allowed for a better assessment of subgroup effects on intake responsiveness. It has also been shown that semi-log models of demand are consistent with economic theory and contain the necessary information for obtaining, for instance, reliable measures of consumer welfare and the underlying preference structure of consumers.(1)

Let q_{ig} represent SSB intake by subgroup g (g: sex and age), p_i and p_j represent the price of SSBs and related good j, and Y and P represent real per capita income and overall food prices (all in country C). SSB intake demand by subgroup g in country C is specified as follows (C subscripts are omitted for convenience):

$$q_{ig} = \beta_0^* + \beta_1^* \ln(Y) + \beta_2^* \ln\left(\frac{p_i}{P}\right) + \beta_3^* \ln\left(\frac{p_j}{P}\right) + \beta_4^* \left[\ln(Y) \times \ln\left(\frac{p_i}{P}\right)\right] + \beta_5^* \ln(Y)^2 + u_{ig} \quad (1)$$

The β terms are coefficients to be estimated and u_{ig} is a random error term. The price terms (p_i and p_j) are deflated by P to discount price differences due to overall food prices and to implicitly account for the cross-price effects of intake categories other than i and j. Note that the structure of the model allows for the relationship between own-price (p_i) and intake to vary by national income level.

We accounted for age, sex, and regional differences by allowing these factors to have a direct effect on intake, as well as an additional effect through income and prices. Thus, the beta terms (β_k^*) were expanded to account for age, sex, and region interactions.

$$\beta_k^* = f(sex, age, region) \forall k \quad (2)$$

The variable *sex* is a binary (= 1 for women and 0 otherwise) and *age* is a variable ranging from 20 to 80 in 5-year intervals. We also considered age^2 to allow for nonlinear age effects and the possibility of optimal responsiveness between the youngest and oldest subgroup. We accounted for varying preferences across countries due to factors not related to income or prices by including six regional binary variables, including: Southeast Asia, East Asia, and High Income Asia Pacific (Asia) (13 countries); Central Europe, Eastern Europe, and Central Asia (CEE/C. Asia) (27); Latin America and the Caribbean (LAC) (30); Middle East, North Africa, and South Asia (MENA/S. Asia) (23); Sub-Saharan Africa (SSA) (45); and High Income/Rest of World (HIC) (26). HIC was comprised largely of Western, industrialized countries; while not geographically connected, these countries share other similarities. We included several small

For peer review only - http://bmjopen.bmj.com/site/about/guidelines.xhtml

island countries in this grouping because they were not sufficiently numerous to merit their own regional grouping.

We first estimated a model with all possible interactions and then utilized F-tests to compare that model to a series of restricted models and arrived at the final parsimonious model. All models were estimated assuming country clusters, that is, independent errors across countries but correlated errors within countries, as well as heteroskedastic-consistent errors.(4)

Given equation (1), the own-price elasticity is derived as follows:

$$\eta_{iig} = \frac{\%\Delta q_{ig}}{\%\Delta p_i} = \frac{1}{q_{ig}}[\beta_2^* + \beta_4^* \ln(Y)] \tag{3}$$

η_{iig} is the percentage change in intake (q_{ig}) (i: SSB) due to a 1% change in p_i, which should be negative since an increase in price usually results in a decrease in intake or quantity demanded. Note that if the β coefficients vary with sex, age, or region, equation (3) will vary accordingly.

Price index adjustment

We used sugar and fresh fruit price indexes as proxies for SSB and fruit juice prices, respectively. An issue with this approach is that sugar and/or fresh fruit may not account for a major share of the final product price, particularly in higher income countries. In view of this fact, we derived a multiplicative adjustment factor for the sugar and fresh fruit price indexes assuming the following quadratic relationship between the adjustment factor and real per-capita income.

$$adjustment = a + bY + cY^2 \tag{4}$$

We used a calibration method to derive values for a, b, and c. We considered the extreme case (zero income) $Y = 0$ and set $a = 1$. In this instance, equation (4) =1 and the price index value would remained unchanged:

$$adjusted\ price\ index = unadjusted\ price\ index \times 1.$$

Using information on the value-added share of farm products in the U.S. food and beverage sector, as well as qualitative information about food production costs in low-income countries, we obtained the following estimates: $b = 0.0003$ and $c = -0.0000000015$.

We adjusted the sugar and fresh fruit price indexes based on equation (4). Note that the adjustment factor starts at a value of 1 and then increases at a decreasing rate with per-capita income indicating a higher value added at higher income levels. For a country in the lowest income decile with per-capita income (Y) = \$1,000, the adjustment factor is 1.3. Assuming an *unadjusted* price index value of 0.70, the *adjusted* price index = 0.91 (0.70 ×1.3). For Switzerland, a high-income country with Y=\$50,963, the unadjusted sugar-price index = 0.63. At this income level, the adjustment factor = 12.39, and the adjusted sugar-price index = 7.81 (0.63 ×12.39). The unadjusted and adjusted sugar price indexes across countries are reported in supplemental figure 2.

For peer review only - http://bmjopen.bmj.com/site/about/guidelines.xhtml

References

1. LaFrance JT. Incomplete demand systems and semilogarithmic demand models. Australian J Agr Econ. 1990;34(2):118-31.
2. von Haefen RH. A complete characterization of the linear, log-linear, and semi-log incomplete demand system models. J Agr Resource Econ. 2002;27(2):281-319.
3. Sahn DE. The Effect of Price and Income Changes on Food-Energy Intake in Sri Lanka. Econ Devel Cult Change. 1988;36(2):315-40.
4. Cameron AC, Trivedi PK. Microeconometrics: Methods and Applications. Cambridge: Cambridge University Press; 2005.

For peer review only

For peer review only - http://bmjopen.bmj.com/site/about/guidelines.xhtml

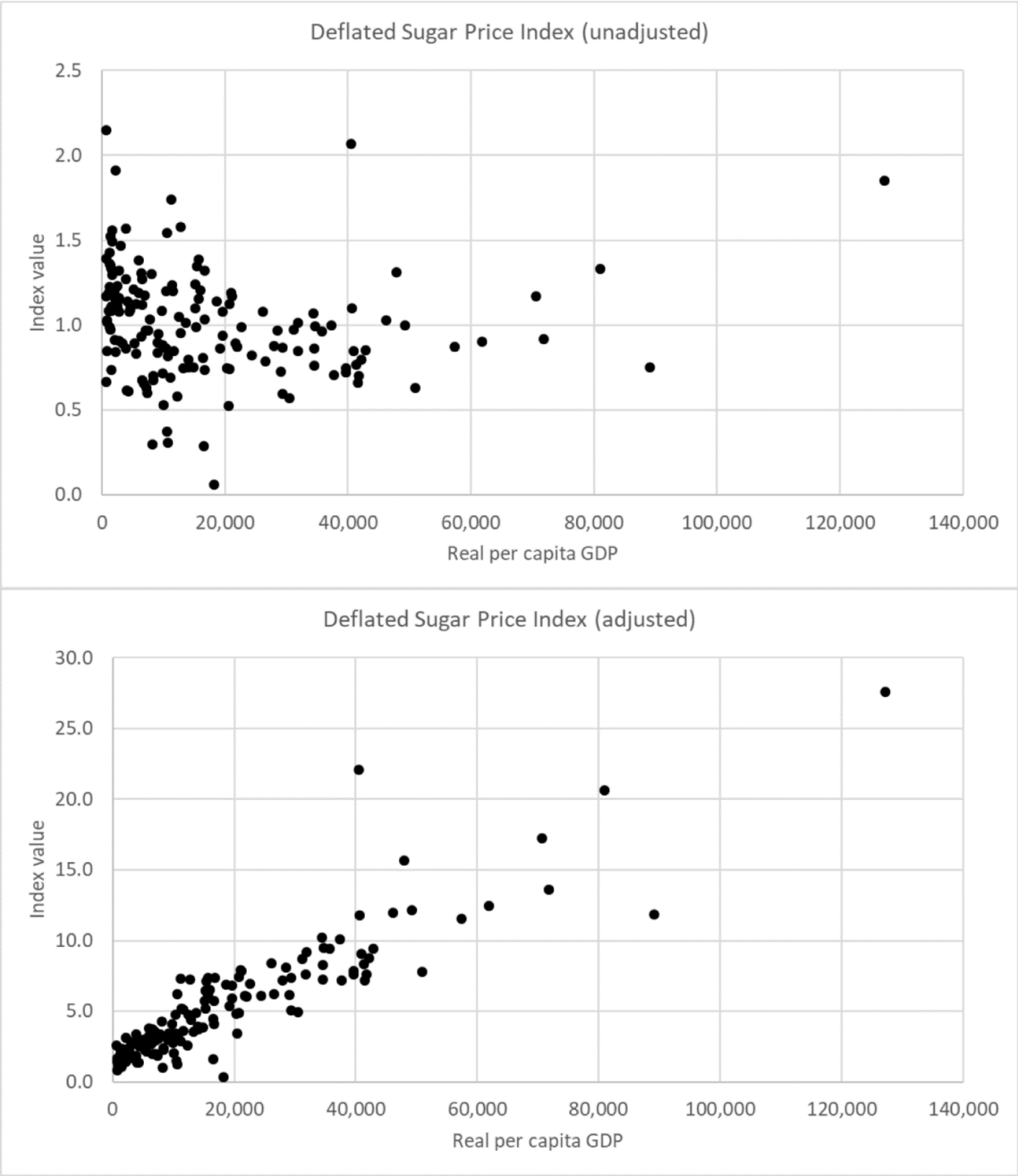

Supplemental Figure 2

Deflated sugar price index: unadjusted and adjusted

Note: prices are deflated by a total food price index to discount differences across countries due to overall food prices.

Source: World Bank International Comparison Program Data. Adjusted price index values are based on author's calculations.
